# Clinical Effectiveness of Ion-Releasing Restorations versus Composite Restorations in Dental Restorations: Systematic Review and Meta-Analysis

**DOI:** 10.3390/dj12060158

**Published:** 2024-05-24

**Authors:** Heber Isac Arbildo-Vega, Fredy Hugo Cruzado-Oliva, Franz Tito Coronel-Zubiate, Sara Antonieta Luján-Valencia, Joan Manuel Meza-Málaga, Rubén Aguirre-Ipenza, Adriana Echevarria-Goche, Eduardo Luján-Urviola, Tania Belú Castillo-Cornock, Katherine Serquen-Olano, Carlos Alberto Farje-Gallardo

**Affiliations:** 1 Faculty of Dentistry, Dentistry School, Universidad San Martín de Porres, Chiclayo 14012, Peru; harbildov@usmp.pe (H.I.A.-V.); tcastilloc@usmp.pe (T.B.C.-C.); kserqueno@usmp.pe (K.S.-O.); 2 Faculty of Human Medicine, Human Medicine School, Universidad San Martín de Porres, Chiclayo 14012, Peru; 3 Faculty of Stomatology, Stomatology School, Universidad Nacional de Trujillo, Trujillo 13001, Peru; fcruzado@unitru.edu.pe; 4 Faculty of Health Sciences, Stomatology School, Universidad Nacional Toribio Rodríguez de Mendoza de Amazonas, Chachapoyas 01001, Peru; carlos.farje@untrm.edu.pe; 5 Postgraduate School, Universidad Católica de Santa María, Arequipa 04013, Peru; slujan@ucsm.edu.pe; 6 Faculty of Dentistry, Dentistry School, Universidad Católica de Santa María, Arequipa 04013, Peru; jmezam@ucsm.edu.pe; 7 Faculty of Medicine, Medicine School, Universidad Católica de Santa María, Arequipa 04013, Peru; 8 Faculty of Health Sciences, Universidad Continental, Lima 15046, Peru; raguirrei@continental.edu.pe; 9 Department of Dentistry, Dentistry School, Norbert Wiener University, Lima 15046, Peru; adriana.echevarria@uwiener.edu.pe; 10 Faculty of Dentistry, Universidad Andina Néstor Cáceres Velásquez, Juliaca 21104, Peru; d02374488@uancv.edu.pe; 11 Faculty of Health Sciences, Stomatology School, Universida Señor de Sipán, Chiclayo 14000, Peru

**Keywords:** ion-releasing restoration, composite resin, glass ionomer cement, review, meta-analysis

## Abstract

Background: To compare the clinical effectiveness of ion-releasing restorations (IRR) vs. composite resin (CR) in dental restorations. Methods: A systematic search was carried out from articles published until January 2024, in the biomedical databases: PubMed, Cochrane Library, Scielo, Scopus, Web of Science and Google Scholar. Randomized clinical trials were included, with a follow-up time greater than or equal to 1 year, without time and language limits and which reported the clinical effect of IRR compared to CR in dental restorations. The RoB 2.0 tool was used to assess the risk of bias of the included studies and the GRADEPro GDT tool was used to assess the quality of evidence and the strength of recommendation of the results. Results: The search yielded a total of 1109 articles. After excluding those that did not meet the selection criteria, 29 articles remained for the quantitative synthesis. The analysis found no statistically significant difference when comparing the dental restorations with IRRs or CRs. Conclusion: The literature reviewed suggests that there are no differences between the IRRs and CRs in dental restorations.

## 1. Introduction

Dentistry is always seeking to raise the quality of restorative interventions, with the intention of guaranteeing patients results that not only last over time, but also exhibit key attributes, such as durability, aesthetics and biocompatibility [1]. The durability and resistance of restorations are essential to face various conditions such as chewing forces and variations in the oral environment [2]. Ensuring long-lasting results is even more crucial in a context where dental caries and other oral conditions continue to be widespread health problems [3]. Aesthetics is a key feature, since the restoration’s appearance is highly valued by our patients [4]. In addition, biocompatibility with surrounding tissues and the influence of this on oral biology contributes to clinical decision making and appropriate material selection [5].

Ion-releasing restorations (IRRs), such as glass ionomers (GICs), are known for their fluoride-releasing properties [6], which are believed to contribute to caries prevention by providing an oral environment that inhibits demineralization of surrounding enamel, and for their biocompatibility with surroundings tissues [7,8,9]. However, marginal adaptation is a crucial point to consider, because its absence could cause secondary problems, and it may not fit the aesthetic expectations of patients [10]. The durability and resistance of IRRs under occlusal loading conditions and other stressors have also shown limitations [11].

Composite resins (CRs) are a popular and effective restorative option, especially in situations where aesthetics are a concern, as they have a wide range of colors and shades that usually closely match the color and natural appearance of the teeth, depending on professional experience in the layering and sculpting technique involved. These CRs usually offer an accurate marginal adaptation after following an appropriate adhesive protocol, avoiding gaps and long-term complications due to their ability to chemically adhere to the tooth through the use of adhesives [12,13,14]. Resistance and durability can be excellent, especially in situations where occlusal forces are not extremely high [14]. However, they are sensitive to moisture, so maintaining a dry working field is crucial for effective adhesion and optimal marginal adaptation [15]. It has also been recognized that they may experience wear due to occlusal contact and other factors [16].

The choice between IRRs or CRs traditionally depends on specific clinical needs, prioritizing either caries prevention and biocompatibility with glass ionomers [17,18], or aesthetics and durability in the case of CRs [1]. Other considerations, such as costs and ease of application, also influence the choice of the most appropriate restorative material for each situation [19].

The purpose of this systematic review and meta-analysis is to determine if there is a difference in clinical effectiveness between IRR materials and CRs in dental restorations, in order to improve dental knowledge and practice based on scientific evidence, offering valuable information to improve the quality of restorative interventions and provide lasting, aesthetic, and biocompatible results that benefit patients.

## 2. Materials and Methods

### 2.1. Protocol

The present review was conducted based on the Preferred Reporting Items for Systematic Reviews and Meta-Analyses Protocols (PRISMA-P) [20] and registered in the Prospective Registry of Systematic Reviews (PROSPERO) [21]. The registry is publicly available under CRD number 42024503374.

The focused question was formulated using the PICO format (population, intervention, outcomes, and results), as detailed below:

- Population: Adults without systematic diseases who received dental restorations.
- Intervention: Restoration with IRRs, which includes all GIC derivatives (RMGIC, HV–GIC, conventional and glass hybrid), polyacid-modified composites (compomer), giomer, and any material declared by the manufacturer to have the ability to release ions.
- Comparison: Restoration with CR.
- Outcomes: Secondary caries or erosion or abfraction, marginal discoloration, marginal adaptation, marginal or tooth integrity, color or translucency, surface texture or luster, surface staining, retention, wear, anatomical form, sensitivity, and state of periodontal tissues.

### 2.2. Focused Question (PICO)

Is there a difference in clinical effectiveness between IRR materials and CR in dental restorations?

### 2.3. Search and Selection of Studies

For the present systematic review, a systematic search was carried out in five electronic databases (PubMed, Cochrane Library, Scopus, Web of Science, and Scielo). Gray literature was also consulted through Google Scholar, OpenGrey, and Proquest. Additionally, the reference lists of included studies were reviewed, all until January 2024, combining keywords and subject titles according to the thesaurus of each database: “ion releasing”, “bioactive resin composite”, “glass ionomer cement”, “high viscosity glass ionomer”, “resin modified glass ionomer”, “glass hybrid”, “polyacid-modified composite”, “compomer”, “resin composite”, “composite resin”, “randomized clinical trial” and “clinical trial”. The search strategies for each of the databases are found in Table 1.

Additionally, further relevant literature was included after a manual search of the reference lists of the final articles.

The search in the electronic database was carried out by two authors (HA and FCO) independently, and the final inclusion decision was made according to the following criteria: Randomized clinical trials (RCTs), with a follow-up time greater than or equal to 1 year, without time and language limits, reporting the clinical effectiveness of IRR and CR in dental restorations (I, II and V Class) using the World Dental Federation (FDI) or the United States Public Health Service Criteria (USPHS) as evaluation criteria. Articles that were prospective studies, unpublished studies, and reported in more than one publication with different follow-up periods were excluded.

### 2.4. Data Extraction

A predefined table was used to extract data from each eligible study, including: author(s), year of publication, study design, country where the study was conducted, number of patients, proportion of male and female patients, age mean and age range, follow-up time, evaluation criteria, study groups, number of patients and teeth restored per study group, type of cavity (according to Black), secondary caries, marginal discoloration, marginal adaptation, marginal integrity, color or translucency, surface texture or luster, surface staining, retention, wear, anatomical form, sensitivity, and periodontal tissues. From each eligible study, two investigators (FCZ and SL) independently extracted information, and all disagreements were resolved by discussion with a third reviewer (JM).

### 2.5. Risk of Bias (RoB) Assessment

The RoB of the included studies was independently assessed by two calibrated authors (RA and AE) (k = 0.98) using the Cochrane Group’s RoB 2.0 tool [21] and all disagreements were resolved by discussion with a third reviewer (EL). According to this tool, clinical trials are evaluated in 5 domains: randomization process, deviations from planned interventions, missing outcome data, outcome measurement, and selection of the results report, later to be classified as high risk of bias, bias with some concerns, or low risk of bias.

### 2.6. Analysis of Results

Data from each study were entered and analyzed in RevMan 5.3 (Cochrane Group, Oxford, UK), using proportions in a random effects model with a 95% confidence interval. Additionally, a GRADE analysis was performed using the guideline development tool (GRADEPro GDT) (McMaster University and Evidence Prime Inc., Hamilton, ON, Canada).

## 3. Results

### 3.1. Selection of Studies

**Figure 1:**
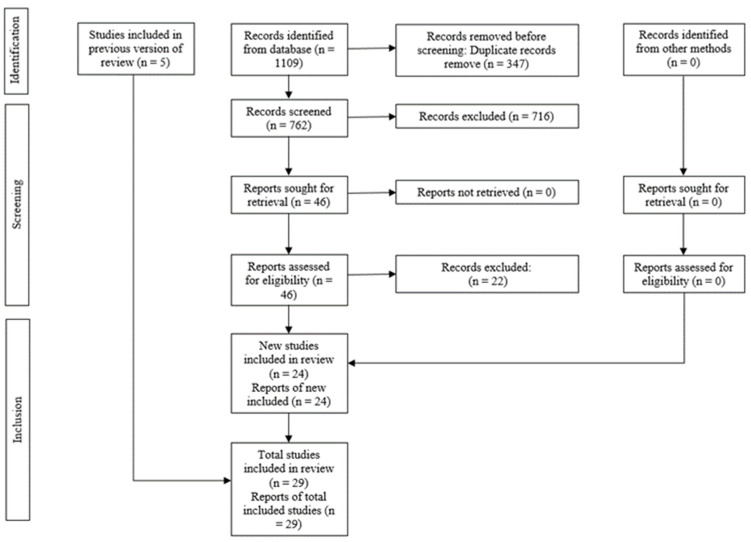
PRISMA diagram showing the process of inclusion and exclusion of studies.

The electronic and manual search strategies yielded a total of 1109 articles, excluding 347 duplicates (Figure 1) and 716 were excluded during title screening, leaving 46 potentially eligible for abstract screening, but 22 articles were excluded and 5 added from other reviews, resulting in 29 RCTs for full-text article screening, and they met the eligibility criteria for qualitative and quantitative synthesis (meta-analysis). The reasons for the exclusion of tudies are found in Table 2.

### 3.2. Characteristics of Included Studies

In total, 29 RCTs [44,45,46,47,48,49,50,51,52,53,54,55,56,57,58,59,60,61,62,63,64,65,66,67,68,69,70,71,72] were included, of which only 1 [50] was parallel. All studies reported that the total number of patients ranged from 10 to 92 and the number of teeth treated ranged from 16 to 180. Sixteen studies [44,47,48,49,50,51,52,54,55,56,61,62,64,65,68,72] reported that the mean age of patients ranged from 25 to 62.2 years, and all studies reported a range of 18 to 92 years in all patients with a follow-up time of between 1 year and 10 years (Table 3).

The countries where the studies were carried out were: Brazil [49,59,60,61,63,66], Croatia [53], Egypt [44,47], Germany [50,70,71], India [45,56,57], Italy [53], Mexico [68], Nigeria [62], Pakistan [46], Romania [58], Serbia [53], Sweeden [55], Turkey [48,51,52,53,54,64,67,69], United Kingdom [65] and the United States [72]. Seven studies [44,47,48,50,53,54,56] mentioned that the evaluation criteria used for the analysis of the teeth were the FDI criteria. Six studies [57,64,65,67,70,71] reported the use of polyacid-modified resin-based composite (PMRC) or compomer, one study [48] used giomer, two studies [53,72] used glass ionomer cement (GIC) or glass hybrid, four studies [47,50,52,54] used high viscosity glass ionomer cement (HVGIC), one study [44] used an ion-releasing restorative (IRR) material and the remaining studies used resin-modified glass ionomer cement (RMGIC) (Table 3).

Twenty-six [44,45,47,48,49,50,51,52,53,54,55,56,58,59,60,61,62,63,64,65,66,67,68,69,71,72], twenty-seven [44,45,47,49,50,51,52,53,54,55,56,57,58,59,60,61,62,63,64,65,66,67,68,69,70,71,72], twenty-one [44,46,47,48,50,51,52,53,54,55,56,57,58,60,61,62,64,65,68,69,72], thirteen [44,45,47,48,49,53,54,59,63,66,67,70,71], seventeen [47,48,49,50,52,54,55,57,58,60,61,64,67,68,69,70,71], sixteen [46,47,48,49,50,52,54,55,57,61,64,67,68,70,71,72], seven [47,48,49,50,53,54,72], twenty-eight [44,45,46,47,48,49,50,51,52,53,54,55,56,57,58,59,60,61,62,63,64,65,66,67,68,69,70,72], nine [47,48,49,50,53,54,61,62,72], twenty [45,47,49,50,52,53,54,55,58,59,60,63,65,66,67,68,69,70,71,72], thirteen [44,47,48,50,51,52,53,54,56,57,61,62,64] and five [47,49,53,54,64] studies reported secondary caries or erosion or abfraction, marginal discoloration, marginal adaptation, marginal or tooth integrity, color or translucency, surface texture or luster, staining surface, retention, wear, anatomical form, sensitivity and state of periodontal tissues, respectively (Table 4).

### 3.3. Risk of Bias Analysis of Studies

**Figure 2:**
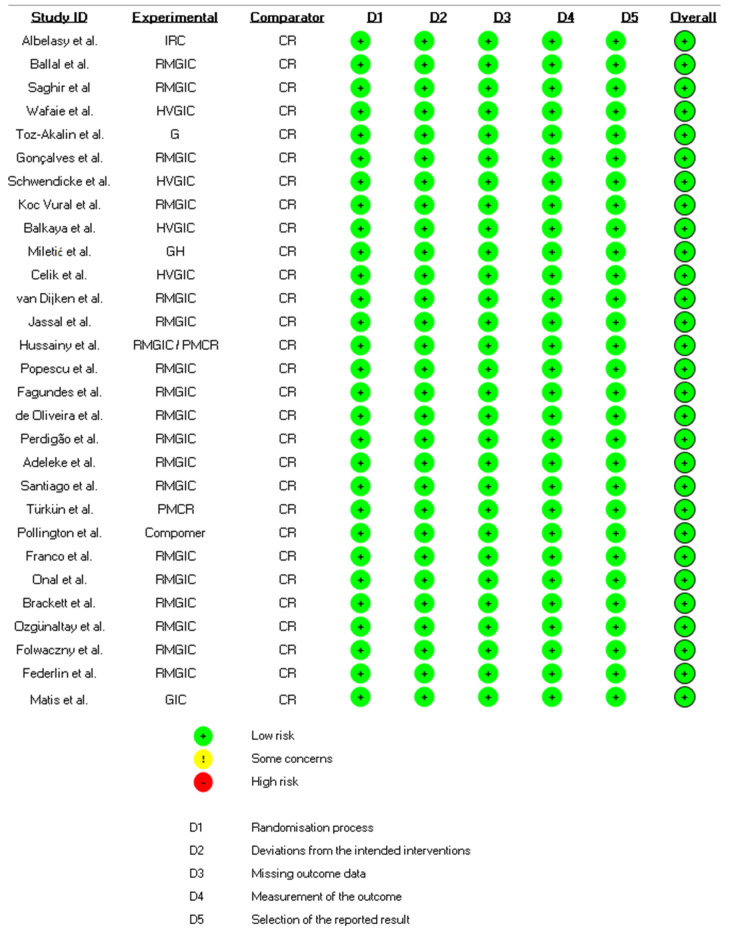
Risk of bias analysis of included studies.

All studies had a low risk of bias (Figure 2).

### 3.4. Synthesis of Results (Meta-Analysis)

The clinical effectiveness of IRR in comparison to CR in terms of absence of secondary caries or erosion or abfraction, absence of marginal discoloration, adequate marginal adaptation, adequate marginal or tooth integrity, adequate color or translucency, proper surface texture or luster, proper surface staining, retention, absence of wear, proper anatomic form, absence of sensitivity and adequate periodontal tissue was determined in twenty-six [44,45,47,48,49,50,51,52,53,54,55,56,58,59,60,61,62,63,64,65,66,67,68,69,71,72], twenty-seven [44,45,47,49,50,51,52,53,54,55,56,57,58,59,60,61,62,63,64,65,66,67,68,69,70,71,72], twenty-one [44,46,47,48,50,51,52,53,54,55,56,57,58,60,61,62,64,65,68,69,72], thirteen [44,45,47,48,49,53,54,59,63,66,67,70,71], seventeen [47,48,49,50,52,54,55,57,58,60,61,64,67,68,69,70,71], sixteen [46,47,48,49,50,52,54,55,57,61,64,67,68,70,71,72], seven [47,48,49,50,53,54,72], twenty-eight [44,45,46,47,48,49,50,51,52,53,54,55,56,57,58,59,60,61,62,63,64,65,66,67,68,69,70,72], nine [47,48,49,50,53,54,61,62,72], twenty [45,47,49,50,52,53,54,55,58,59,60,63,65,66,67,68,69,70,71,72], thirteen [44,47,48,50,51,52,53,54,56,57,61,62,64] and five [47,49,53,54,64] studies, which show that there was no statistically significant difference (*p* > 0.05) for all these clinical parameters (Supplementary Figures S1–S12).

### 3.5. Subgroup Synthesis

The meta-analysis of clinical effectiveness of IRR in comparison to CR in terms of absence of secondary caries or erosion or abfraction, absence of marginal discoloration, adequate marginal adaptation, adequate marginal or tooth integrity, adequate color or translucency, proper surface texture or luster, proper surface staining, retention, absence of wear, proper anatomic form, absence of sensitivity and adequate periodontal tissue, and in relation to the restorative material used, type of cavity, evaluation criteria and follow-up time, showed that there was no statistically significant difference (*p* > 0.05) for all these clinical parameters (Supplementary Figures S13–S60).

### 3.6. GRADE Analysis

When evaluating the included studies, it was observed that there is high certainty in the absence of marginal discoloration, adequate marginal adaptation, adequate marginal or tooth integrity, adequate color or translucency, proper surface staining, absence of wear, absence of sensitivity and adequate periodontal tissues, and there is moderate certainty in the absence of secondary caries or erosion or abfraction, proper anatomic form, proper surface texture or luster and retention (Table 5).

## 4. Discussion

It has been observed that the clinical effectiveness of IRR and CR was similar in the 12 parameters evaluated: secondary caries or erosion or abfraction, marginal adaptation, marginal integrity, color or translucency, surface texture or gloss, surface staining, retention, wear, anatomical shape, sensitivity, marginal discoloration, and periodontal tissue, in restoring carious lesions. To address these aspects, 12 meta-analyses were carried out, each independently evaluating a specific parameter, in relation to the restorative material, considering variables such as the type of cavity (Black’s I, II and V class), the evaluation criterion (FDI and USPHS) and follow-up periods of 12, 24, 36 and 60 months. To determine the general strength of the evidence for each MA, the Grade analysis was performed, revealing that there is high certainty in the parameters of marginal adaptation, marginal integrity, color or translucency, surface staining, wear, sensitivity, marginal discoloration, and periodontal tissue, and there is moderate certainty regarding the parameters of secondary caries or erosion or abfraction, surface texture or gloss, retention, and anatomical shape.

Of the 29 studies analyzed in this systematic review, it is observed that glass ionomer and its derivatives are the most widely reported IRRs in the clinical literature; however, giomer and compomers were less commonly used, possibly due to their status as newer technology in dental restorations.

According to the literature, dental restorations have failure or replacement rates of 60%, with secondary caries being one of the main causes [73]. Secondary caries is considered a complex and polymicrobial dysbiosis, resulting from an imbalance in the demineralization (DEM) and remineralization (REM) process that occurs between restoration and cavity preparation [73]. The margins of restorations can be considered critical areas due to the possible presence of spaces or gaps produced by polymerization contraction, porosity, or fractures. Under these circumstances, biofilm accumulation is facilitated, which increases the degradation of restorations and can lead to the formation of caries lesions [74]. Furthermore, secondary caries is influenced by several factors, the most common being lesion location, patient’s caries risk, age and socioeconomic status, variation in operator skills, and detection methods and criteria [75].

In this review, most of the included studies evaluated secondary caries in Black V class cavity types; however, these restorations are less affected than Black I and II classes, which could influence the low incidence. Secondary caries has been reported to be more common in deep proximal restorations with gingival margins extending beyond the CEJ, with dentin and cementum as the tooth substrate [76]. All included studies are controlled clinical trials where the oral hygiene index of patients is moderate to good; therefore, the risk of cavities is low. Consequently, the risk of secondary caries formation in these patients was considered low, regardless of the material used, which is consistent with the results of other reviews [75,77,78].

However, in people at high risk of dental caries, a frequent decrease in pH is observed, which requires additional sources of ions, such as fluoride, to effectively contribute to the control of the DEM–REM process, as found in IRRs [79]. In another review, this parameter was shown to be more effective in glass ionomers and their derivatives, due to the lack of homogeneity in the included studies [74].

Furthermore, it is associated with polymerization contraction, which can cause the formation of spaces between the restoration and the cavity wall, compromising the marginal integrity of the restoration and leading to the clinical appearance of pigmentation at the margins of the restoration. This marginal discoloration can influence the longevity of the restoration due to marginal microleakage, which leads to deterioration of the restoration. However, currently, this drawback has been solved with various adhesive systems that have the ability to seal the microporosities found between the tooth–restoration interface [56]. This advance could explain the clinical effectiveness of both materials, as has been corroborated in the reviews carried out by Becerra et al. [77] and Albelasy et al. [75].

The results obtained in this review regarding the retention parameter did not show significant differences between the materials evaluated. Most of the included studies focused on Black V class carious lesions, where the lack of macro-mechanical retention is inherent to this type of restoration. Retention is affected by several factors, such as tooth bending, occlusal stress, dentin characteristics, etch pattern, components of bonding agents, and elastic modulus of restorative materials. Therefore, adhesion is the most crucial factor; however, its effectiveness is compromised by its degradation [64,67]. Fortunately, with technological advances, modern adhesive systems have seen significant improvements [66].

Currently, the bonding mechanisms of new materials derived from glass ionomers and composite resins are similar. In addition to their micro-mechanical retention, both have the potential for chemical bonding to the tooth. These interact superficially with the dentin and do not completely dissolve the hydroxyapatite crystals around the collagen, thus allowing chemical bonding [56]. It has been observed that universal adhesives applied using etch and rinse and selective etching modes tend to achieve superior clinical results [80]. However, in a systematic review and meta-analysis, it was found that the retention rate was higher in glass ionomer restorations compared to composite resin restorations, possibly due to the greater number of restorations made with glass ionomer in the included studies [77].

The color or translucency of the restorative material is affected by incomplete polymerization, susceptibility to water sorption, and desiccation [57]. It is also influenced by patient factors, such as oral microflora and the absorption of pigments due to dietary habits [56]. However, with the advent of modern finishing and polishing systems, significant improvement have been observed in preventing discoloration of restorative materials [81].

The anatomical shape is related to the chemical composition, type, and amount of filler, and can affect the degree of wear of the restorations. Reduced filler content may result in greater polishability, gloss or surface texture, but may decrease overall wear resistance. Currently, the composition of materials derived from glass ionomers and CR are similar [57], which could explain the efficiency of these restorative materials. These findings are consistent with the results of the review conducted by Bezerra et al. [77].

Postoperative sensitivity has been attributed to a variety of factors, including operative trauma, depth of injury, desiccation, microleakage, etc. However, the time spent and effort invested in the proper application of the placement technique of restorative materials, together with the clinically proven properties, minimize the hydrostatic movement of the dentinal fluid. This phenomenon could explain the absence of postoperative sensitivity observed in both materials during the follow-up periods of 12 to 60 months [47].

The observation of similar results between IRR and CR across a wide range of clinical parameters underscores the potential of both materials in the dental restorative field. However, the significant heterogeneity among the studies, including differences in design, treated populations, and techniques used, reminds us of the need for caution in interpreting these findings. The observed variability suggests that the applicability of the results may vary depending on the specific clinical context, highlighting the importance of considering individual patient circumstances in clinical decision making.

In light of this reality, there is a pressing need for future research that is not only methodologically rigorous and standardized but also focused on reducing heterogeneity to strengthen the available evidence. This need emphasizes the importance of interdisciplinary collaboration among dentists, researchers, and material manufacturers, promoting the development and continuous improvement of restorative materials. By addressing these challenges and expanding our understanding of the clinical effectiveness of IRR and CR, we can move towards the common goal of improving the quality of restorative interventions and more effectively meeting the changing needs of patients and professionals in dentistry.

## 5. Conclusions

According to the findings of the present review, there are no differences when restoring teeth with CRs or IRRs. However, these results cannot be considered conclusive due to the high heterogeneity of the included studies in some clinical parameters, and the moderate strength of the clinical practice recommendations presented in some clinical parameters of the included studies.

## Figures and Tables

**Table 1:** Database search strategy.

Database	Search Strategy	Number of Study
Pubmed	((“ion releasing”) OR (“bioactive resin composite”) OR (“glass ionomer cement”) OR (“high viscosity glass ionomer”) OR (“resin modified glass ionomer”) OR (“glass hybrid”) OR (“polyacid-modified composite”) OR (“compomer”)) AND ((“resin composite”) OR (“composite resin”)) AND ((“randomized clinical trial”) OR (“clinical trial”))	174
Cochrane Library	#1 MeSH descriptor: [Glass Ionomer Cements] explode all trees #2 MeSH descriptor: [Compomers] explode all trees #3 (“ion releasing”) OR (“bioactive resin composite”) OR (“glass ionomer cement”) OR (“high viscosity glass ionomer”) OR (“resin modified glass ionomer”) OR (“glass hybrid”) OR (“polyacid-modified composite”) OR (“compomer”) (Word variations have been searched) #4 #1 OR #2 OR #3 #5 MeSH descriptor: [Composite Resins] explode all trees #6 (“Composite resin”) OR (“Resin composite”) (Word variations have been searched) #7 #5 OR #6 #8 MeSH descriptor: [Clinical Trial] explode all trees #9 (“randomized clinical trial”) OR (“clinical trial”) (Word variations have been searched) #10 #8 OR #9 #11 #4 AND #7 AND #10	183
Scielo	((“ion releasing”) OR (“bioactive resin composite”) OR (“glass ionomer cement”) OR (“high viscosity glass ionomer”) OR (“resin modified glass ionomer”) OR (“glass hybrid”) OR (“polyacid-modified composite”) OR (“compomer”)) AND ((“resin composite”) OR (“composite resin”)) AND ((“randomized clinical trial”) OR (“clinical trial”))	3
Scopus	(TITLE-ABS-KEY ((“ion releasing”) OR (“bioactive resin composite”) OR (“glass ionomer cement”) OR (“high-viscosity glass ionomer”) OR (“resin-modified glass ionomer”) OR (“glass hybrid”) OR (“polyacid-modified composite”) OR (“compomer”)) AND TITLE-ABS-KEY (((“resin composite”) OR (“composite resin”))) AND TITLE-ABS-KEY ((“randomized clinical trial”) OR (“clinical trial”))) AND (LIMIT-TO (DOCTYPE, “ar”))	494
Web of Science	(TS = (“ion releasing”) OR TS = (“bioactive resin composite”) OR TS = (“glass ionomer cement”) OR TS = (“high viscosity glass ionomer”) OR TS = (“resin modified glass ionomer”) OR TS = (“glass hybrid”) OR TS = (“polyacid-modified composite”) OR TS = (“compomer”)) AND (TS = (“resin composite”) OR TS = (“composite resin”)) AND (TS = (“randomized clinical trial”) OR TS = (“clinical trial”))	80
Google Scholar	allintitle: “ion releasing” OR “bioactive resin composite” OR “glass ionomer cement” OR “high viscosity glass ionomer” OR “resin modified glass ionomer” OR “glass hybrid” OR “polyacid modified composite” OR “compomer” OR “resin composite” OR “composite resin” “clinical trial”-“systematic review”-“in vitro”-“review”	156
Open Gray	((“ion releasing”) OR (“bioactive resin composite”) OR (“glass ionomer cement”) OR (“high viscosity glass ionomer”) OR (“resin modified glass ionomer”) OR (“glass hybrid”) OR (“polyacid-modified composite”) OR (“compomer”)) AND ((“resin composite”) OR (“composite resin”)) AND ((“randomized clinical trial”) OR (“clinical trial”))	0
Proquest	(“ion releasing” OR “bioactive resin composite” OR “glass ionomer cement” OR “high viscosity glass ionomer” OR “resin modified glass ionomer” OR “glass hybrid” OR “polyacid-modified composite” OR “compomer”) AND (“resin composite” OR “composite resin”) AND (“randomized clinical trial” OR “clinical trial”) NOT (“systematic review” OR “in vitro” OR “review”)	19

**Table 2:** Reason for exclusion of studies.

Author	Reason for Exclusion
Gonçalves et al. [22]	Study with data reported in another publication with different follow-up period
Shinohara et al. [23]	
Balkaya et al. [24]	
Çelik et al. [25]	
Brackett et al. [26]	
Folwaczny et al. [27]	
Koubi et al. [28]	Non-randomized clinical trials
Gallo et al. [29]	
Wucher et al. [30]	
Powell et al. [31]	
Smales et al. [32]	
Burgess et al. [33]	The full text was not found
Neo et al. [34]	
van Dijken et al. [35]	
Wilkie et al. [36]	
Lidums et al. [37]	
Kaurich et al. [38]	
Osborne et al. [39]	
Gupta et al. [40]	Studies with different evaluation criteria
Isler et al. [41]	
van Dijken et al. [42]	
Burrow et al. [43]	

**Table 3:** Characteristics of included studies.

Authors	Year	Study Design	Country	Number of Patients (Male/Female)	Average Age (Range)	Follow-Up	Evaluation Criteria	Groups	Number of Patients per Group	Number of Teeth per Group	Class (Black)
Albelasy et al. [44]	2024	RCT cross-over	Egypt	32 (10/22)	29.4 ± 8 (>18)	2 years	FDI	IRC	32	32	I and II
								IRC	32	32	
								CR	32	32	
Ballal et al. [45]	2023	RCT cross-over	India	40	(18–60)	1 year	USPHS	RMGIC	40	40	V
								CR	40	40	
Saghir et al. [46]	2023	RCT cross-over	Pakistan	30	(30–60)	1 year	USPHS	RMGIC	30	30	V
								FCR	30	30	
Wafaie et al. [47]	2023	RCT cross-over	Egypt	40 (26/14)	25 (20–40)	5 years	FDI	HVGIC	40	40	II
								HVGIC	40	40	
								HVGIC	40	40	
								CR	40	40	
Toz-Akalin et al. [48]	2023	RCT cross-over	Turkey	35 (18/17)	29 ± 9 (18–47)	2 years	FDI	G	35	35	I and II
								CR	35	35	
Gonçalves et al. [49]	2021	RCT cross-over	Brazil	50 (34/16)	61 (38–92)	3 years	USPHS	RMGIC	50	50	V
								RMGIC + EDTA	50	50	
								CR	50	50	
								CR + SE	50	50	
Schwendicke et al. [50]	2021	RCT parallel	Germany	88 (45/43)	62.2 ± 5.7 (50–70)	3 years	FDI	HVGIC	43	81	V
								CR	45	88	
Koc Vural et al. [51]	2021	RCT cross-over	Turkey	33 (10/23)	52.69 ± 9.7 (37–89)	3 years	USPHS	RMGIC	33	55	V
								CR	33	55	
Balkaya et al. [52]	2020	RCT cross-over	Turkey	54 (23/31)	22 (20–32)	2 years	USPHS	HVGIC	54	34	II
								CR	54	37	
								CR	54	38	
Miletić et al. [53]	2020	RCT cross-over	Croatia, Italy, Turkey and Serbia	180 (64/116)	(>18)	2 years	FDI	GH	180	180	II
								CR	180	180	
Celik et al. [54]	2019	RCT cross-over	Turkey	22 (11/11)	47.8 (34–63)	3 years	FDI	HVGIC	22	67	V
								CR	22	67	
van Dijken et al. [55]	2019	RCT cross-over	Sweeden	67 (38/29)	58.3 (37–86)	1 year	USPHS	RMGIC	67	82	I and II
								CR	67	82	
Jassal et al. [56]	2018	RCT cross-over	India	56 (44/12)	54 (>18)	1.5 years	FDI	RMGIC	56	98	V
								CR + P-SEA	56	98	
								CR + A-SEA	56	98	
Hussainy et al. [57]	2018	RCT cross-over	India	NR	(18–65)	1 year	USPHS	RMGIC	NR	33	V
								PMRC	NR	34	
								CR	NR	34	
Popescu et al. [58]	2016	RCT cross-over	Romania	45	(25–65)	2 years	USPHS	RMGIC	45	73	V
								CR	45	74	
								RMGIC + CR	45	73	
Fagundes et al. [59]	2014	RCT cross-over	Brazil	30	(18–50)	7 years	USPHS	RMGIC	30	35	V
								CR	30	35	
de Oliveira et al. [60]	2012	RCT cross-over	Brazil	10 (3/7)	(36–55)	1 year	USPHS	RMGIC	10	40	V
								RMGIC + AS	10	43	
								CR	10	41	
Perdigão et al. [61]	2012	RCT cross-over	Brazil	33	48.7 (30–79)	1 year	USPHS	RMGIC	33	31	V
								RMGIC	33	30	
								CR	33	31	
Adeleke et al. [62]	2012	RCT cross-over	Nigeria	44 (32/12)	52 ± 12 (25–74)	1 year	USPHS	RMGIC	44	170	V
								CR	44	168	
Santiago et al. [63]	2010	RCT cross-over	Brazil	30	(18–50)	2 years	USPHS	RMGIC	30	35	V
								CR	30	35	
Türkün et al. [64]	2008	RCT cross-over	Turkey	24 (12/12)	44 (25–54)	2 years	USPHS	PMRC	24	50	V
								CR	24	50	
Pollington et al. [65]	2008	RCT cross-over	United Kingdom	30	54	3 years	USPHS	Compomer	30	30	V
								CR	30	30	
Franco et al. [66]	2006	RCT cross-over	Brazil	30	(18–50)	5 years	USPHS	RMGIC	30	35	V
								CR	30	35	
Onal et al. [67]	2005	RCT cross-over	Turkey	30	(27–64)	2 years	USPHS	RMGIC	30	24	V
								PMRC	30	38	
								PMRC	30	46	
								CR	30	22	
Brackett et al. [68]	2003	RCT cross-over	Mexico	24	47 (28–73)	2 years	USPHS	RMGIC	24	37	V
								CR	24	37	
Ozgünaltay et al. [69]	2002	RCT cross-over	Turkey	24	(40–65)	3 years	USPHS	RMGIC	24	50	V
								CR	24	50	
Folwaczny et al. [70]	2001	RCT cross-over	Germany	37	(26–67)	3 years	USPHS	RMGIC	37	51	V
								RMGIC	37	31	
								CR	37	36	
								PMRC	37	79	
Federlin et al. [71]	1998	RCT cross-over	Germany	11 (5/6)	(30–77)	1 year	USPHS	RMGIC	11	16	V
								PMRC	11	16	
								CR	11	16	
Matis et al. [72]	1996	RCT cross-over	United State	30 (18/12)	58 (29–76)	10 years	USPHS	GIC-IF	30	30	V
								GIC-DF	30	30	
								GIC	30	30	
								CR	30	30	

RCT = Randomized clinical trial; USPHS = United States Public Health Service; FDI = World Dental Federation; IRCs = Ion-releasing composites; RMGIC = Resin-modified glass-ionomer cement; CR = Composite resin; FCR = Flowable composite resin; SE = Selective enamel; EDTA = Ethylene diamine tetra acetic acid; HVGIC = Highly viscous glass-ionomer cement; P-SEA = Passive self-etch adhesive; A-SEA = Active self-etch adhesive; AS = Adhesive system; PMRC = Polyacid-modified resin-based composite; GIC-IF = Glass-ionomer cement immediate finished; GIC-DF = Glass-ionomer cement delay finished; NR = Not reported.

**Table 4:** Characteristics of included studies.

Authors	Year	Groups	Absence of Secondary Caries, Erosion or Abfraction	Absence of Marginal Discoloration	Adequate Marginal Adaptation	Adequate Marginal or Tooth Integrity	Adequate Color or Translucency	Proper Surface Texture or Luster	Proper Surface Staining	Retention	Absence of Wear	Proper Anatomic Form	Absence of Sensibility	Adequate Periodontal Tissue
Albelasy et al. [44]	2024	IRC	6 m: 31/31 1 y: 29/29 2 y: 27/27	6 m: 31/31 1 y: 29/29 2 y: 27/27	6 m: 31/31 1 y: 29/29 2 y: 27/27	6 m: 31/31 1 y: 29/29 2 y: 27/27	NR	NR	NR	6 m: 31/31 1 y: 29/29 2 y: 27/27	NR	NR	6 m: 31/31 1 y: 29/29 2 y: 27/27	NR
		IRC	6 m: 31/31 1 y: 29/29 2 y: 27/27	6 m: 31/31 1 y: 29/29 2 y: 27/27	6 m: 31/31 1 y: 29/29 2 y: 27/27	6 m: 31/31 1 y: 29/29 2 y: 27/27	NR	NR	NR	6 m: 31/31 1 y: 29/29 2 y: 26/27	NR	NR	6 m: 31/31 1 y: 29/29 2 y: 27/27	NR
		CR	6 m: 31/31 1 y: 29/29 2 y: 27/27	6 m: 31/31 1 y: 29/29 2 y: 27/27	6 m: 31/31 1 y: 29/29 2 y: 27/27	6 m: 31/31 1 y: 29/29 2 y: 27/27	NR	NR	NR	6 m: 31/31 1 y: 29/29 2 y: 27/27	NR	NR	6 m: 31/31 1 y: 29/29 2 y: 27/27	NR
Ballal et al. [45]	2023	RMGIC	1 m: 40/40 6 m: 40/40 1 y: 40/40	1 m: 39/40 6 m: 32/40 1 y: 24/40	NR	1 m: 39/40 6 m: 29/40 1 y: 23/40	NR	NR	NR	1 m: 39/40 6 m: 32/40 1 y: 24/40	NR	1 m: 39/40 6 m: 30/40 1 y: 22/40	NR	NR
		CR	1 m: 40/40 6 m: 40/40 1 y: 40/40	1 m: 40/40 6 m: 39/40 1 y: 33/40	NR	1 m: 40/40 6 m: 39/40 1 y: 31/40	NR	NR	NR	1 m: 40/40 6 m: 39/40 1 y: 33/40	NR	1 m: 40/40 6 m: 39/40 1 y: 32/40	NR	NR
Saghir et al. [46]	2023	RMGIC	NR	NR	1 y: 23/30	NR	NR	1 y: 25/30	NR	1 y: 28/30	NR	NR	NR	NR
		FCR	NR	NR	1 y: 21/30	NR	NR	1 y: 18/30	NR	1 y: 19/30	NR	NR	NR	NR
Wafaie et al. [47]	2023	HVGIC	1 y: 40/40 3 y: 38/38 5 y: 38/38	1 y: 40/40 3 y: 38/38 5 y: 38/38	1 y: 40/40 3 y: 38/38 5 y: 38/38	1 y: 40/40 3 y: 38/38 5 y: 38/38	1 y: 40/40 3 y: 38/38 5 y: 38/38	1 y: 40/40 3 y: 38/38 5 y: 38/38	1 y: 40/40 3 y: 38/38 5 y: 38/38	1 y: 40/40 3 y: 38/39 5 y: 38/38	1 y: 40/40 3 y: 38/38 5 y: 38/38	1 y: 40/40 3 y: 38/38 5 y: 38/38	1 y: 40/40 3 y: 38/38 5 y: 38/38	1 y: 40/40 3 y: 38/38 5 y: 38/38
		HVGIC	1 y: 40/40 3 y: 39/39 5 y: 37/37	1 y: 40/40 3 y: 39/39 5 y: 37/37	1 y: 40/40 3 y: 39/39 5 y: 37/37	1 y: 40/40 3 y: 39/39 5 y: 39/39	1 y: 40/40 3 y: 39/39 5 y: 37/37	1 y: 40/40 3 y: 39/39 5 y: 37/37	1 y: 40/40 3 y: 39/39 5 y: 37/37	1 y: 40/40 3 y: 39/39 5 y: 37/39	1 y: 40/40 3 y: 39/39 5 y: 37/37	1 y: 40/40 3 y: 39/39 5 y: 37/37	1 y: 40/40 3 y: 39/39 5 y: 39/39	1 y: 40/40 3 y: 39/39 5 y: 39/39
		HVGIC	1 y: 40/40 3 y: 38/38 5 y: 38/38	1 y: 40/40 3 y: 38/38 5 y: 38/38	1 y: 40/40 3 y: 38/38 5 y: 37/38	1 y: 40/40 3 y: 39/39 5 y: 38/38	1 y: 40/40 3 y: 38/38 5 y: 38/38	1 y: 40/40 3 y: 38/38 5 y: 38/38	1 y: 40/40 3 y: 38/38 5 y: 38/38	1 y: 40/40 3 y: 38/39 5 y: 38/38	1 y: 40/40 3 y: 38/38 5 y: 38/38	1 y: 40/40 3 y: 38/38 5 y: 37/38	1 y: 40/40 3 y: 39/39 5 y: 38/38	1 y: 40/40 3 y: 39/39 5 y: 38/38
		CR	1 y: 40/40 3 y: 39/39 5 y: 39/39	1 y: 40/40 3 y: 39/39 5 y: 39/39	1 y: 40/40 3 y: 39/39 5 y: 39/39	1 y: 40/40 3 y: 39/39 5 y: 39/39	1 y: 40/40 3 y: 39/39 5 y: 39/39	1 y: 40/40 3 y: 39/39 5 y: 39/39	1 y: 40/40 3 y: 39/39 5 y: 39/39	1 y: 40/40 3 y: 39/39 5 y: 39/39	1 y: 40/40 3 y: 39/39 5 y: 39/39	1 y: 40/40 3 y: 39/39 5 y: 39/39	1 y: 40/40 3 y: 39/39 5 y: 39/39	1 y: 40/40 3 y: 39/39 5 y: 39/39
Toz-Akalin et al. [48]	2023	G	2 y: 28/28		2 y: 28/28	2 y: 28/28	2 y: 28/28	2 y: 28/28	2 y: 28/28	2 y: 28/29	2 y: 28/28	NR	2 y: 28/28	NR
		CR	2 y: 29/29		2 y: 29/29	2 y: 29/29	2 y: 29/29	2 y: 29/29	2 y: 29/29	2 y: 29/29	2 y: 29/29	NR	2 y: 29/29	NR
Gonçalves et al. [49]	2021	RMGIC	1 y: 48/48 2 y: 47/47 3 y: 42/42	1 y: 45/48 2 y: 42/47 3 y: 29/42	NR	1 y: 44/48 2 y: 38/47 3 y: 30/42	1 y: 33/48 2 y: 33/47 3 y: 27/42	1 y: 47/48 2 y: 43/47 3 y: 34/42	1 y: 48/48 2 y: 46/47 3 y: 40/42	1 y: 48/49 2 y: 47/48 3 y: 42/43	1 y: 48/48 2 y: 46/47 3 y: 39/42	1 y: 46/48 2 y: 45/47 3 y: 40/42	NR	1 y: 48/48 2 y: 47/47 3 y: 42/42
		RMGIC + EDTA	1 y: 49/49 2 y: 47/47 3 y: 40/40	1 y: 48/49 2 y: 41/47 3 y: 30/40	NR	1 y: 44/49 2 y: 37/47 3 y: 30/40	1 y: 38/49 2 y: 35/47 3 y: 32/40	1 y: 47/49 2 y: 42/47 3 y: 32/40	1 y: 49/49 2 y: 47/47 3 y: 40/40	1 y: 49/49 2 y: 47/48 3 y: 40/42	1 y: 49/49 2 y: 47/47 3 y: 38/40	1 y: 46/49 2 y: 44/47 3 y: 38/40	NR	1 y: 49/49 2 y: 47/47 3 y: 40/40
		CR	1 y: 47/47 2 y: 44/44 3 y: 37/37	1 y: 41/47 2 y: 35/44 3 y: 25/37	NR	1 y: 38/47 2 y: 35/44 3 y: 25/37	1 y: 36/47 2 y: 36/44 3 y: 29/37	1 y: 45/47 2 y: 43/44 3 y: 35/37	1 y: 45/47 2 y: 43/44 3 y: 35/37	1 y: 47/49 2 y: 44/48 3 y: 37/42	1 y: 47/47 2 y: 44/44 3 y: 36/37	1 y: 46/47 2 y: 43/44 3 y: 36/37	NR	1 y: 46/47 2 y: 43/44 3 y: 36/37
		CR + SE	1 y: 48/48 2 y: 47/47 3 y: 41/41	1 y: 43/48 2 y: 37/47 3 y: 25/41	NR	1 y: 43/48 2 y: 40/47 3 y: 30/41	1 y: 38/48 2 y: 38/47 3 y: 32/41	1 y: 43/48 2 y: 43/47 3 y: 36/41	1 y: 47/48 2 y: 45/47 3 y: 39/41	1 y: 48/49 2 y: 47/48 3 y: 41/42	1 y: 48/48 2 y: 46/47 3 y: 38/41	1 y: 48/48 2 y: 47/47 3 y: 41/41	NR	1 y: 48/48 2 y: 47/47 3 y: 41/41
Schwendicke et al. [50]	2021	HVGIC	1.5 y: 67/67 3y: 41/41	1.5 y: 67/67 3y: 41/41	1.5 y: 65/67 3y: 40/41	NR	1.5 y: 67/67 3y: 41/41	1.5 y: 67/67 3y: 41/41	1.5 y: 67/67 3y: 41/41	1.5 y: 64/77 3y: 39/41	1.5 y: 67/67 3y: 41/41	1.5 y: 66/67 3y: 41/41	1.5 y: 67/67 3y: 41/41	NR
		CR	1.5 y: 70/70 3y: 49/49	1.5 y: 70/70 3y: 49/49	1.5 y: 70/70 3y: 49/49	NR	1.5 y: 70/70 3y: 49/49	1.5 y: 70/70 3y: 49/49	1.5 y: 70/70 3y: 49/49	1.5 y: 70/77 3y: 49/49	1.5 y: 70/70 3y: 49/49	1.5 y: 70/70 3y: 49/49	1.5 y: 70/70 3y: 49/49	NR
Koc Vural et al. [51]	2021	RMGIC	6 m: 54/54 1 y: 52/52 1.5 y: 48/48 2 y: 47/47 3 y: 47/47	6 m: 53/54 1 y: 51/52 1.5 y: 33/48 2 y: 31/47 3 y: 31/47	6 m: 54/54 1 y: 52/52 1.5 y: 48/48 2 y: 47/47 3 y: 47/47	NR	NR	NR	NR	6 m: 54/55 1 y: 52/53 1.5 y: 48/51 2 y: 47/51 3 y: 47/51	NR	NR	6 m: 54/54 1 y: 52/52 1.5 y: 48/48 2 y: 47/47 3 y: 47/47	NR
		CR	6 m: 55/55 1 y: 52/52 1.5 y: 43/43 2 y: 43/43 3 y: 43/43	6 m: 55/55 1 y: 52/52 1.5 y: 40/43 2 y: 40/43 3 y: 40/43	6 m: 55/55 1 y: 52/52 1.5 y: 43/43 2 y: 43/43 3 y: 43/43	NR	NR	NR	NR	6 m: 55/55 1 y: 52/53 1.5 y: 43/51 2 y: 43/51 3 y: 43/51	NR	NR	6 m: 55/55 1 y: 52/52 1.5 y: 43/43 2 y: 43/43 3 y: 43/43	NR
Balkaya et al. [52]	2020	HVGIC	1 y: 32/32 2 y: 21/21	1 y: 32/32 2 y: 20/21	1 y: 30/32 2 y: 20/21	NR	1 y: 6/32 2 y: 15/21	1 y: 31/32 2 y: 19/21	NR	1 y: 24/32 2 y: 15/21	NR	1 y: 30/32 2 y: 20/21	1 y: 32/32 2 y: 21/21	NR
		CR	1 y: 35/35 2 y: 32/32	1 y: 35/35 2 y: 32/32	1 y: 35/35 2 y: 32/32	NR	1 y: 35/35 2 y: 32/32	1 y: 35/35 2 y: 32/32	NR	1 y: 35/35 2 y: 32/32	NR	1 y: 35/35 2 y: 32/32	1 y: 35/35 2 y: 32/32	NR
		CR	1 y: 36/36 2 y: 31/31	1 y: 36/36 2 y: 31/31	1 y: 36/36 2 y: 31/31	NR	1 y: 36/36 2 y: 31/31	1 y: 36/36 2 y: 31/31	NR	1 y: 36/36 2 y: 31/31	NR	1 y: 36/36 2 y: 31/31	1 y: 36/36 2 y: 31/31	NR
Miletić et al. [53]	2020	GH	1 y: 162/162 2 y: 143/143	1 y: 161/161 2 y: 142/142	1 y: 161/161 2 y: 142/142	1 y: 162/162 2 y: 143/143	NR	NR	1 y: 162/162 2 y: 143/143	1 y: 162/162 2 y: 143/143	1 y: 162/162 2 y: 143/143	1 y: 162/162 2 y: 143/143	1 y: 162/162 2 y: 143/143	1 y: 162/162 2 y: 144/144
		CR	1 y: 162/162 2 y: 143/143	1 y: 161/161 2 y: 142/142	1 y: 161/161 2 y: 142/142	1 y: 162/162 2 y: 143/143	NR	NR	1 y: 162/162 2 y: 143/143	1 y: 161/162 2 y: 141/143	1 y: 162/162 2 y: 143/143	1 y: 162/162 2 y: 143/143	1 y: 162/162 2 y: 143/143	1 y: 162/162 2 y: 144/144
Celik et al. [54]	2019	HVGIC	6 m: 66/66 1 y: 63/63 2 y: 52/52 3 y: 47/47	6 m: 66/66 1 y: 63/63 2 y: 52/52 3 y: 47/47	6 m: 66/66 1 y: 63/63 2 y: 52/52 3 y: 47/47	6 m: 66/66 1 y: 63/63 2 y: 52/52 3 y: 47/47	6 m: 66/66 1 y: 63/63 2 y: 52/52 3 y: 47/47	6 m: 66/66 1 y: 63/63 2 y: 52/52 3 y: 47/47	6 m: 66/66 1 y: 63/63 2 y: 52/52 3 y: 47/47	6 m: 66/66 1 y: 63/63 2 y: 52/53 3 y: 47/49	6 m: 66/66 1 y: 63/63 2 y: 52/52 3 y: 47/47	6 m: 66/66 1 y: 63/63 2 y: 52/52 3 y: 47/47	6 m: 66/66 1 y: 63/63 2 y: 52/52 3 y: 47/47	6 m: 66/66 1 y: 63/63 2 y: 52/52 3 y: 47/47
		CR	6 m: 67/67 1 y: 67/67 2 y: 57/57 3 y: 54/54	6 m: 67/67 1 y: 67/67 2 y: 57/57 3 y: 54/54	6 m: 67/67 1 y: 67/67 2 y: 57/57 3 y: 54/54	6 m: 67/67 1 y: 67/67 2 y: 57/57 3 y: 54/54	6 m: 67/67 1 y: 67/67 2 y: 57/57 3 y: 54/54	6 m: 67/67 1 y: 67/67 2 y: 57/57 3 y: 54/54	6 m: 67/67 1 y: 67/67 2 y: 57/57 3 y: 54/54	6 m: 67/67 1 y: 67/67 2 y: 57/57 3 y: 54/54	6 m: 67/67 1 y: 67/67 2 y: 57/57 3 y: 54/54	6 m: 67/67 1 y: 67/67 2 y: 57/57 3 y: 54/54	6 m: 67/67 1 y: 67/67 2 y: 57/57 3 y: 54/54	6 m: 67/67 1 y: 67/67 2 y: 55/57 3 y: 52/54
van Dijken et al. [55]	2019	RMGIC	1 y: 3/82	1 y: 82/82	1 y: 74/82	NR	1 y: 77/82	1 y: 82/82	NR	1 y: 62/82	NR	1 y: 74/82	NR	NR
		CR	1 y: 82/82	2 y: 82/82	2 y: 81/82	NR	2 y: 82/82	2 y: 82/82	NR	2 y: 80/82	NR	2 y: 81/82	NR	NR
Jassal et al. [56]	2018	RMGIC	6 m: 95/95 1 y: 90/90 1.5 y: 90/90	6 m: 95/95 1 y: 90/90 1.5 y: 90/90	6 m: 95/95 1 y: 90/90 1.5 y: 90/90	NR	NR	NR	NR	6 m: 95/98 1 y: 90/98 1.5 y: 90/98	NR	NR	6 m: 95/95 1 y: 90/90 1.5 y: 90/90	NR
		CR + P-SEA	6 m: 93/93 1 y: 88/88 1.5 y: 86/86	6 m: 92/93 1 y: 87/88 1.5 y: 85/86	6 m: 92/93 1 y: 87/88 1.5 y: 85/86	NR	NR	NR	NR	6 m: 93/98 1 y: 88/98 1.5 y: 86/98	NR	NR	6 m: 93/93 1 y: 88/88 1.5 y: 86/86	NR
		CR + A-SEA	6 m: 96/96 1 y: 93/93 1.5 y: 92/92	6 m: 96/96 1 y: 93/93 1.5 y: 92/92	6 m: 96/96 1 y: 93/93 1.5 y: 92/92	NR	NR	NR	NR	6 m: 96/98 1 y: 93/98 1.5 y: 92/98	NR	NR	6 m: 96/96 1 y: 93/93 1.5 y: 92/92	NR
Hussainy et al. [57]	2018	RMGIC	NR	6 m: 33/33 1 y: 32/33	6 m: 33/33 1 y: 32/33	NR	6 m: 33/33 1 y: 32/33	6 m: 33/33 1 y: 32/33	NR	6 m: 33/33 1 y: 32/33	NR	NR	6 m: 33/33 1 y: 32/33	NR
		PMRC	NR	6 m: 33/34 1 y: 33/34	6 m: 33/34 1 y: 33/34	NR	6 m: 33/34 1 y: 33/34	6 m: 33/34 1 y: 33/34	NR	6 m: 33/34 1 y: 33/34	NR	NR	6 m: 33/34 1 y: 33/34	NR
		CR	NR	6 m: 32/34 1 y: 32/34	6 m: 32/34 1 y: 32/34	NR	6 m: 32/34 1 y: 32/34	6 m: 32/34 1 y: 32/34	NR	6 m: 32/34 1 y: 32/34	NR	NR	6 m: 32/34 1 y: 32/34	NR
Popescu et al. [58]	2016	RMGIC	6 m: 73/73 1 y: 57/57 1.5 y: 57/57 2 y: 57/57	6 m: 73/73 1 y: 49/57 1.5 y: 38/57 2 y: 32/57	6 m: 73/73 1 y: 49/57 1.5 y: 39/57 2 y: 32/57	NR	6 m: 41/73 1 y: 32/57 1.5 y: 32/57 2 y: 50/57	NR	NR	6 m: 73/73 1 y: 57/57 1.5 y: 57/57 2 y: 54/57	NR	6 m: 73/73 1 y: 57/57 1.5 y: 57/57 2 y: 57/57	NR	NR
		CR	6 m: 74/74 1 y: 58/58 1.5 y: 57/57 2 y: 53/53	6 m: 74/74 1 y: 52/58 1.5 y: 37/57 2 y: 31/53	6 m: 74/74 1 y: 52/58 1.5 y: 39/57 2 y: 30/53	NR	6 m: 74/74 1 y: 57/58 1.5 y: 51/57 2 y: 44/53	NR	NR	6 m: 74/74 1 y: 58/58 1.5 y: 57/58 2 y: 53/57	NR	6 m: 74/74 1 y: 58/58 1.5 y: 57/57 2 y: 53/53	NR	NR
		RMGIC + CR	6 m: 73/73 1 y: 57/57 1.5 y: 54/54 2 y: 48/48	6 m: 73/73 1 y: 54/57 1.5 y: 39/54 2 y: 30/48	6 m: 73/73 1 y: 54/57 1.5 y: 38/54 2 y: 28/48	NR	6 m: 73/73 1 y: 55/57 1.5 y: 45/54 2 y: 37/48	NR	NR	6 m: 73/73 1 y: 57/57 1.5 y: 54/57 2 y: 48/57	NR	6 m: 73/73 1 y: 57/57 1.5 y: 54/54 2 y: 48/48	NR	NR
Fagundes et al. [59]	2014	RMGIC	6 m: 34/34 1 y: 35/35 2 y: 33/33 5 y: 27/27 7 y: 23/23	6 m: 34/34 1 y: 35/35 2 y: 33/33 5 y: 27/27 7 y: 23/23	NR	6 m: 34/34 1 y: 35/35 2 y: 33/33 5 y: 23/27 7 y: 20/23	NR	NR	NR	6 m: 34/34 1 y: 35/35 2 y: 33/33 5 y: 27/28 7 y: 23/26	NR	6 m: 34/34 1 y: 35/35 2 y: 33/33 5 y: 23/27 7 y: 21/23	NR	NR
		CR	6 m: 30/30 1 y: 30/30 2 y: 26/26 5 y: 15/17 7 y: 12/13	6 m: 30/30 1 y: 30/30 2 y: 26/26 5 y: 17/17 7 y: 13/13	NR	6 m: 30/30 1 y: 30/30 2 y: 26/26 5 y: 13/17 7 y: 9/13	NR	NR	NR	6 m: 30/34 1 y: 30/35 2 y: 26/33 5 y: 17/27 7 y: 13/25	NR	6 m: 29/30 1 y: 29/30 2 y: 25/26 5 y: 15/17 7 y: 12/13	NR	NR
de Oliveira et al. [60]	2012	RMGIC	1 y: 38/38	1 y: 38/38	1 y: 38/38	NR	1 y: 38/38	NR	NR	1 y: 38/40	NR	1 y: 38/38	NR	NR
		RMGIC + AS	1 y: 43/43	1 y: 43/43	1 y: 43/43	NR	1 y: 43/43	NR	NR	1 y: 43/43	NR	1 y: 43/43	NR	NR
		CR	1 y: 41/41	1 y: 41/41	1 y: 41/41	NR	1 y: 41/41	NR	NR	1 y: 41/41	NR	1 y: 41/41	NR	NR
Perdigão et al. [61]	2012	RMGIC	6 m: 28/28 1 y: 26/26	6 m: 28/28 1 y: 25/26	6 m: 26/28 1 y: 26/26	NR	6 m: 28/28 1 y: 26/26	6 m: 16/28 1 y: 11/26	NR	6 m: 28/28 1 y: 26/26	6 m: 28/28 1 y: 26/26	NR	6 m: 28/28 1 y: 25/26	NR
		N-RMGIC	6 m: 27/27 1 y: 25/25	6 m: 26/27 1 y: 15/25	6 m: 21/27 1 y: 17/25	NR	6 m: 16/27 1 y: 15/25	6 m: 24/27 1 y: 23/25	NR	6 m: 27/27 1 y: 25/25	6 m: 26/27 1 y: 24/25	NR	6 m: 27/27 1 y: 24/25	NR
		CR	6 m: 27/29 1 y: 25/27	6 m: 25/29 1 y: 22/27	6 m: 23/29 1 y: 23/27	NR	6 m: 25/29 1 y: 22/27	6 m: 26/29 1 y: 25/27	NR	6 m: 27/29 1 y: 25/27	6 m: 27/29 1 y: 25/27	NR	6 m: 27/29 1 y: 24/27	NR
Adeleke et al. [62]	2012	RMGIC	6 m: 154/154 1 y: 117/117	6 m: 136/136 1 y: 130/131	6 m: 136/136 1 y: 131/131	NR	NR	NR	NR	6 m: 136/148 1 y: 131/144	6 m: 136/136 1 y: 131/131	NR	6 m: 136/136 1 y: 131/131	NR
		CR	6 m: 86/86 1 y: 72/72	6 m: 115/115 1 y: 105/106	6 m: 115/115 1 y: 105/106	NR	NR	NR	NR	6 m: 115/147 1 y: 106/143	6 m: 115/115 1 y: 106/106	NR	6 m: 115/115 1 y: 106/106	NR
Santiago et al. [63]	2010	RMGIC	6 m: 35/35 1 y: 35/35 2 y: 33/33	6 m: 35/35 1 y: 35/35 2 y: 33/33	NR	6 m: 35/35 1 y: 35/35 2 y: 33/33	NR	NR	NR	6 m: 35/35 1 y: 35/35 2 y: 33/33	NR	6 m: 35/35 1 y: 35/35 2 y: 33/33	NR	NR
		CR	6 m: 31/31 1 y: 30/30 2 y: 26/26	6 m: 31/31 1 y: 30/30 2 y: 26/26	NR	6 m: 31/31 1 y: 30/30 2 y: 26/26	NR	NR	NR	6 m: 31/35 1 y: 30/35 2 y: 26/33	NR	6 m: 31/31 1 y: 29/30 2 y: 25/26	NR	NR
Türkün et al. [64]	2008	PMRC	6 m: 50/50 1 y: 50/50 2 y: 50/50	6 m: 50/50 1 y: 50/50 2 y: 50/50	6 m: 50/50 1 y: 50/50 2 y: 50/50	NR	6 m: 50/50 1 y: 50/50 2 y: 50/50	6 m: 50/50 1 y: 50/50 2 y: 50/50	NR	6 m: 50/50 1 y: 50/50 2 y: 48/50	NR	NR	6 m: 50/50 1 y: 50/50 2 y: 50/50	6 m: 50/50 1 y: 50/50 2 y: 50/50
		CR	6 m: 50/50 1 y: 50/50 2 y: 50/50	6 m: 50/50 1 y: 50/50 2 y: 50/50	6 m: 50/50 1 y: 50/50 2 y: 50/50	NR	6 m: 48/50 1 y: 48/50 2 y: 47/50	6 m: 50/50 1 y: 50/50 2 y: 50/50	NR	6 m: 50/50 1 y: 50/50 2 y: 50/50	NR	NR	6 m: 50/50 1 y: 50/50 2 y: 50/50	6 m: 50/50 1 y: 50/50 2 y: 50/50
Pollington et al. [65]	2008	C	6 m: 30/30 1 y: 30/30 3 y: 30/30	6 m: 29/30 1 y: 28/30 3 y: 28/30	6 m: 26/30 1 y: 25/30 3 y: 24/30	NR	NR	NR	NR	6 m: 28/30 1 y: 27/30 3 y: 26/30	NR	6 m: 27/30 1 y: 27/30 3 y: 27/30	NR	NR
		CR	6 m: 30/30 1 y: 30/30 3 y: 30/30	6 m: 29/30 1 y: 29/30 3 y: 28/30	6 m: 28/30 1 y: 27/30 3 y: 25/30	NR	NR	NR	NR	6 m: 28/30 1 y: 28/30 3 y: 26/30	NR	6 m: 30/30 1 y: 29/30 3 y: 29/30	NR	NR
Franco et al. [66]	2006	RMGIC	1 y: 35/35 2 y: 33/33 5 y: 27/27	1 y: 35/35 2 y: 33/33 5 y: 27/27	NR	1 y: 35/35 2 y: 33/33 5 y: 23/27	NR	NR	NR	1 y: 35/35 2 y: 33/33 5 y: 27/28	NR	1 y: 35/35 2 y: 33/33 5 y: 23/27	NR	NR
		CR	1 y: 30/30 2 y: 26/26 5 y: 15/17	1 y: 30/30 2 y: 26/26 5 y: 17/17	NR	1 y: 30/30 2 y: 26/26 5 y: 13/17	NR	NR	NR	1 y: 30/35 2 y: 26/33 5 y: 27/33	NR	1 y: 29/30 2 y: 25/26 5 y: 15/17	NR	NR
Onal et al. [67]	2005	RMGIC	1 y: 24/24 2 y: 24/24	1 y: 24/24 2 y: 24/24	NR	1 y: 24/24 2 y: 24/24	1 y: 24/24 2 y: 24/24	1 y: 24/24 2 y: 24/24	NR	1 y: 24/24 2 y: 24/24	NR	1 y: 24/24 2 y: 24/24	NR	NR
		PMRC	1 y: 32/32 2 y: 21/21	1 y: 32/32 2 y: 21/21	NR	1 y: 32/32 2 y: 21/21	1 y: 32/32 2 y: 21/21	1 y: 32/32 2 y: 21/21	NR	1 y: 32/38 2 y: 21/32	NR	1 y: 32/32 2 y: 21/21	NR	NR
		PMRC	1 y: 38/38 2 y: 26/26	1 y: 38/38 2 y: 26/26	NR	1 y: 38/38 2 y: 26/26	1 y: 38/38 2 y: 26/26	1 y: 38/38 2 y: 26/26	NR	1 y: 38/46 2 y: 26/38	NR	1 y: 38/38 2 y: 26/26	NR	NR
		CR	1 y: 18/18 2 y: 13/13	1 y: 18/18 2 y: 13/13	NR	1 y: 18/18 2 y: 13/13	1 y: 18/18 2 y: 13/13	1 y: 18/18 2 y: 13/13	NR	1 y: 18/22 2 y: 13/18	NR	1 y: 18/18 2 y: 13/13	NR	NR
Brackett et al. [68]	2003	RMGIC	6 m: 31/31 1 y: 30/30 1.5 y: 30/30 2 y: 26/26	6 m: 31/31 1 y: 30/30 1.5 y: 30/30 2 y: 26/26	6 m: 31/31 1 y: 30/30 1.5 y: 30/30 2 y: 26/26	NR	6 m: 31/31 1 y: 30/30 1.5 y: 30/30 2 y: 26/26	6 m: 31/31 1 y: 30/30 1.5 y: 30/30 2 y: 26/26	NR	6 m: 31/32 1 y: 30/31 1.5 y: 30/31 2 y: 26/27	NR	6 m: 31/31 1 y: 30/30 1.5 y: 30/30 2 y: 26/26	NR	NR
		CR	6 m: 28/28 1 y: 26/26 1.5 y: 26/26 2 y: 22/22	6 m: 28/28 1 y: 26/26 1.5 y: 26/26 2 y: 22/22	6 m: 28/28 1 y: 26/26 1.5 y: 26/26 2 y: 22/22	NR	6 m: 28/28 1 y: 26/26 1.5 y: 26/26 2 y: 22/22	6 m: 28/28 1 y: 26/26 1.5 y: 26/26 2 y: 22/22	NR	6 m: 28/32 1 y: 26/31 1.5 y: 26/31 2 y: 22/27	NR	6 m: 28/28 1 y: 26/26 1.5 y: 26/26 2 y: 22/22	NR	NR
Ozgünaltay et al. [69]	2002	RMGIC	6 m: 48/48 1 y: 48/48 2 y: 44/44 3 y: 44/44	6 m: 48/48 1 y: 48/48 2 y: 44/44 3 y: 44/44	6 m: 48/48 1 y: 48/48 2 y: 44/44 3 y: 44/44	NR	6 m: 48/48 1 y: 48/48 2 y: 44/44 3 y: 44/44	NR	NR	6 m: 48/48 1 y: 48/48 2 y: 44/45 3 y: 44/45	NR	6 m: 48/48 1 y: 48/48 2 y: 44/44 3 y: 44/44	NR	NR
		CR	6 m: 45/45 1 y: 45/45 2 y: 40/40 3 y: 40/40	6 m: 45/45 1 y: 45/45 2 y: 40/40 3 y: 40/40	6 m: 45/45 1 y: 45/45 2 y: 40/40 3 y: 40/40	NR	6 m: 45/45 1 y: 45/45 2 y: 40/40 3 y: 40/40	NR	NR	6 m: 45/45 1 y: 45/45 2 y: 40/42 3 y: 40/42	NR	6 m: 45/45 1 y: 45/45 2 y: 40/40 3 y: 40/40	NR	NR
Folwaczny et al. [70]	2001	RMGIC	NR	3 y: 28/31	NR	3 y: 24/31	3 y: 31/31	3 y: 31/31	NR	3 y: 31/33	NR	3 y: 29/31	NR	NR
		RMGIC	NR	3 y: 19/23	NR	3 y: 17/23	3 y: 23/23	3 y: 23/23	NR	3 y: 23/26	NR	3 y: 14/23	NR	NR
		CR	NR	3 y: 20/22	NR	3 y: 20/22	3 y: 22/22	3 y: 22/22	NR	3 y: 22/23	NR	3 y: 22/22	NR	NR
		PMRC	NR	3 y: 40/43	NR	3 y: 36/43	3 y: 43/43	3 y: 43/43	NR	3 y: 43/48	NR	3 y: 40/43	NR	NR
Federlin et al. [71]	1998	RMGIC	1 y: 15/15	1 y: 15/15	NR	1 y: 15/15	1 y: 15/15	1 y: 15/15	NR	NR	NR	1 y: 15/15	NR	NR
		PMRC	1 y: 15/15	1 y: 15/15	NR	1 y: 15/15	1 y: 15/15	1 y: 15/15	NR	NR	NR	1 y: 15/15	NR	NR
		CR	1 y: 15/15	1 y: 15/15	NR	1 y: 15/15	1 y: 15/15	1 y: 15/15	NR	NR	NR	1 y: 15/15	NR	NR
Matis et al. [72]	1996	GIC-IF	10 y: 15/15	10 y: 15/15	10 y: 13/15	NR	NR	10 y: 15/15	10 y: 14/15	6 m: 29/29 1 y: 29/29 3 y: 27/30 5 y: 27/30 10 y: 15/18	10 y: 14/15	10 y: 13/15	NR	NR
		GIC-DF	10 y: 14/14	10 y: 14/14	10 y: 12/14	NR	NR	10 y: 14/14	10 y: 14/14	6 m: 29/29 1 y: 29/29 3 y: 29/30 5 y: 28/30 10 y: 14/18	10 y: 12/14	10 y: 14/14	NR	NR
		GIC	10 y: 12/12	10 y: 12/12	10 y: 10/12	NR	NR	10 y: 12/12	10 y: 11/12	6 m: 28/29 1 y: 28/29 3 y: 28/30 5 y: 28/30 10 y: 12/18	10 y: 9/12	10 y: 8/12	NR	NR
		CR	10 y: 3/3	10 y: 3/3	10 y: 2/3	NR	NR	10 y: 3/3	10 y: 3/3	6 m: 22/29 1 y: 20/29 3 y: 15/30 5 y: 13/30 10 y: 3/18	10 y: 3/3	10 y: 3/3	NR	NR

IRC = Ion-releasing composites; RMGIC = Resin-modified glass-ionomer cement; CR = Composite resin; FCR = Flowable composite resin; C = Compomer; SE = Selective enamel; EDTA = Ethylene diamine tetra acetic acid; HVGIC = Highly viscous glass-ionomer cement; P-SEA = Passive self-etch adhesive; A-SEA = Active self-etch adhesive; AS = Adhesive system; PMRC = Polyacid-modified resin-based composite; GIC-IF = Glass-ionomer cement immediate finished; GIC-DF = Glass-ionomer cement delay finished; m = months; y = year(s); NR = Not reported.

**Table 5:** GRADE analysis of included studies.

Certainty Assessment							N° of Patients		Effect		Certainty
N° of Studies	Study Design	Risk of Bias	Inconsistency	Indirectness	Imprecision	Other Considerations	IRR	CR	Relative (95% CI)	Absolute (95% CI)	
Absence of secondary caries or erosion or abfraction (follow-up: range 1 year to 10 years)											
26	randomized trials	not serious	serious	not serious	not serious	none	1088/1167 (93.2%)	1083/1089 (99.4%)	RR 1.00 (0.97 to 1.04)	0 fewer per 1000 (from 30 fewer to 40 more)	⨁⨁⨁◯ Moderate
Absence of marginal discoloration (follow-up: range 1 year to 10 years)											
27	randomized trials	not serious	not serious	not serious	not serious	none	1128/1208 (93.4%)	1093/1149 (95.1%)	RR 1.00 (0.99 to 1.01)	0 fewer per 1000 (from 10 fewer to 10 more)	⨁⨁⨁⨁ High
Adequate marginal adaptation (follow-up: range 1 year to 10 years)											
21	randomized trials	not serious	not serious	not serious	not serious	none	988/1039 (95.1%)	979/1025 (95.5%)	RR 1.00 (0.99 to 1.01)	0 fewer per 1000 (from 10 fewer to 10 more)	⨁⨁⨁⨁ High
Adequate marginal or tooth integrity (follow-up: range 1 year to 5 years)											
13	randomized trials	not serious	not serious	not serious	not serious	none	468/511 (91.6%)	442/475 (93.1%)	RR 1.00 (0.99 to 1.01)	0 fewer per 1000 (from 9 fewer to 9 more)	⨁⨁⨁⨁ High
Adequate color or translucency (follow-up: range 1 year to 5 years)											
17	randomized trials	not serious	not serious	not serious	not serious	None	600/634 (94.6%)	612/639 (95.8%)	RR 1.00 (0.98 to 1.02)	0 more per 1000 (from 19 fewer to 19 more)	⨁⨁⨁⨁ High
Proper surface texture or luster (follow-up: range 1 year to 10 years)											
16	randomized trials	not serious	serious	not serious	not serious	none	506/537 (94.2%)	520/538 (96.7%)	RR 0.99 (0.97 to 1.02)	10 fewer per 1000 (from 29 fewer to 19 more)	⨁⨁⨁◯ Moderate
Proper surface staining (follow-up: range 2 years to 10 years)											
7	randomized trials	not serious	not serious	not serious	not serious	none	347/350 (99.1%)	352/354 (99.4%)	RR 1.00 (0.99 to 1.01)	0 fewer per 1000 (from 10 fewer to 10 more)	⨁⨁⨁⨁ High
Retention (follow-up: range 1 year to 10 years)											
28	randomized trials	not serious	serious	not serious	not serious	none	1195/1300 (91.9%)	1174/1317 (89.1%)	RR 1.02 (0.98 to 1.06)	9 more per 1000 (from 18 fewer to 53 more)	⨁⨁⨁◯ Moderate
Absence of wear (follow-up: range 1 year to 10 years)											
9	randomized trials	not serious	not serious	not serious	not serious	none	501/507 (98.8%)	484/487 (99.4%)	RR 1.00 (0.99 to 1.01)	0 fewer per 1000 (from 10 fewer to 10 more)	⨁⨁⨁⨁ High
Proper anatomic form (follow-up: range 1 year to 10 years)											
20	randomized trials	not serious	serious	not serious	not serious	none	752/805 (93.4%)	756/771 (98.1%)	RR 0.98 (0.95 to 1.01)	20 fewer per 1000 (from 49 fewer to 10 more)	⨁⨁⨁◯ Moderate
Absence of sensibility (follow-up: range 1 years to 5 years)											
13	randomized trials	not serious	not serious	not serious	not serious	none	721/723 (99.7%)	720/725 (99.3%)	RR 1.00 (0.99 to 1.01)	0 fewer per 1000 (from 10 fewer to 10 more)	⨁⨁⨁⨁ High
Adequate periodontal tissue (follow-up: range 2 years to 5 years)											
5	randomized trials	not serious	not serious	not serious	not serious	none	322/322 (100.0%)	321/324 (99.1%)	RR 1.00 (0.99 to 1.01)	0 more per 1000 (from 10 fewer to 10 more)	⨁⨁⨁⨁ High

IRRs = Ion-releasing restorations; CR = Composite resin; CI = Confidence interval; RR = Risk ratio

## Supplementary Materials

The following are available online at https://www.mdpi.com/article/10.3390/dj12060158/s1, The meta-analyses of the data extracted from the included studies is presented. The data involved the following 12 clinical parameters: absence of secondary caries or erosion or abfraction, absence of marginal discoloration, adequate marginal adaptation, adequate marginal or tooth integrity, adequate color or translucency, proper surface texture or luster, proper surface staining, retention, absence of wear, proper anatomic form, absence of sensibility and adequate periodontal tissue (Figures S1–S60). The results are presented as forest plots; where: CR = composite resin; RMGIC = Resin-modified glass-ionomer cement; HVGIC = Highly viscous glass-ionomer cement; PMRC = Polyacid-modified resin-based composite; FDI = World Dental Federation criteria; USPHS = United States Public Health Service Criteria; CI = confidence interval; df = degrees of freedom; M-H = Mantel-Haenszel; I^2^ = Higgins I^2^ test; Chi^2^ = Chi square test.
