# Supplementary material for: Clinical Effectiveness of Ion-Releasing Restorations versus Composite Restorations in Dental Restorations: Systematic Review and Meta-Analysis
Source: Dent J (Basel). 2024 May 24;12(6):158. doi: 10.3390/dj12060158 (PMC11203382; doi:10.3390/dj12060158)

Supplementary Materials

Figure S1. Absence of secondary caries or erosion or abfraction between IRR and CR in dental restorations

Study or Subgroup	Ion-releasing restorative material Events	Total	Composite resin Events	Total	Weight	Risk Ratio M-H, Random, 95% CI
Adeleke et al. 2012	117	117	72	72	5.0%	1.00 [0.98, 1.02]
Albelasy et al. 2024	27	27	27	27	4.2%	1.00 [0.93, 1.07]
Balkaya et al. 2020	21	21	32	32	4.0%	1.00 [0.93, 1.08]
Ballal et al. 2023	40	40	40	40	4.6%	1.00 [0.95, 1.05]
Brackett et al. 2003	26	26	22	22	4.0%	1.00 [0.92, 1.08]
Celik et al. 2019	47	47	54	54	4.8%	1.00 [0.96, 1.04]
de Oliveira et al. 2012	38	38	41	41	4.6%	1.00 [0.95, 1.05]
Fagundes et al. 2014	23	23	12	13	1.9%	1.10 [0.91, 1.33]
Federlin et al. 1998	15	15	15	15	3.0%	1.00 [0.88, 1.13]
Franco et al. 2006	27	27	15	17	1.9%	1.14 [0.94, 1.38]
Gonçalves et al. 2021	42	42	37	37	4.6%	1.00 [0.95, 1.05]
Jassal et al. 2018	90	90	92	92	5.0%	1.00 [0.98, 1.02]
Koc Vural et al. 2021	47	47	43	43	4.7%	1.00 [0.96, 1.04]
Matis et al. 1996	12	12	3	3	0.6%	1.00 [0.68, 1.47]
Miletić et al. 2020	143	143	143	143	5.1%	1.00 [0.99, 1.01]
Onal et al. 2005	24	24	13	13	3.2%	1.00 [0.89, 1.12]
Ozgünaltay et al. 2002	44	44	40	40	4.7%	1.00 [0.96, 1.05]
Perdigão et al. 2012	26	26	25	27	2.9%	1.08 [0.95, 1.22]
Pollington et al. 2008	30	30	30	30	4.3%	1.00 [0.94, 1.07]
Popescu et al. 2016	57	57	53	53	4.8%	1.00 [0.97, 1.04]
Santiago et al. 2010	33	33	26	26	4.3%	1.00 [0.94, 1.07]
Schwendicke et al. 2021	41	41	49	49	4.7%	1.00 [0.96, 1.04]
Toz-Akalin et al. 2023	28	28	28	29	3.6%	1.03 [0.94, 1.14]
Türkün et al. 2008	50	50	50	50	4.8%	1.00 [0.96, 1.04]
van Dijken et al. 2019	3	82	82	82	0.1%	0.04 [0.02, 0.12]
Wafaie et al. 2023	37	37	39	39	4.6%	1.00 [0.95, 1.05]
**Total (95% CI)**		**1167**		**1089**	**100.0%**	**1.00 [0.97, 1.04]**
Total events	1088		1083			

Heterogeneity: Tau² = 0.01; Chi² = 322.94, df = 25 (P < 0.00001); I² = 92%
Test for overall effect: Z = 0.26 (P = 0.80)

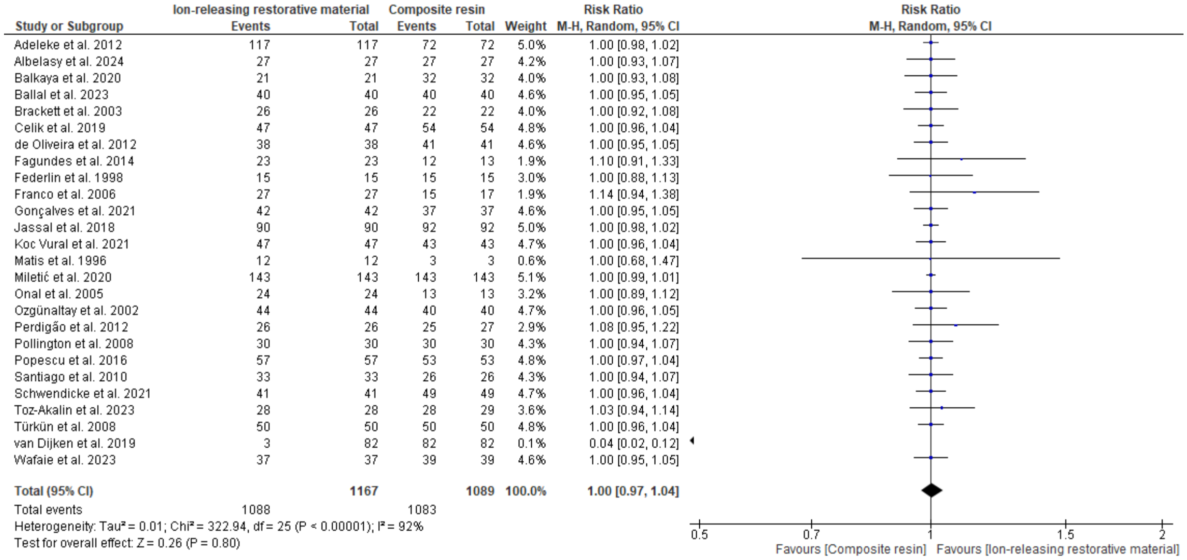

Figure S2. Absence of marginal discoloration between IRR and CR in dental restorations

Study or Subgroup	Ion-releasing restorative material Events	Total	Composite resin Events	Total	Weight	Risk Ratio M-H, Random, 95% CI
Adeleke et al. 2012	130	131	105	106	10.9%	1.00 [0.98, 1.03]
Albelasy et al. 2024	27	27	27	27	2.7%	1.00 [0.93, 1.07]
Balkaya et al. 2020	20	21	32	32	1.0%	0.95 [0.84, 1.07]
Ballal et al. 2023	24	40	33	40	0.2%	0.73 [0.54, 0.97]
Brackett et al. 2003	26	26	22	22	2.2%	1.00 [0.92, 1.08]
Celik et al. 2019	47	47	54	54	6.7%	1.00 [0.96, 1.04]
de Oliveira et al. 2012	38	38	41	41	4.8%	1.00 [0.95, 1.05]
Fagundes et al. 2014	23	23	13	13	1.1%	1.00 [0.89, 1.12]
Federlin et al. 1998	15	15	15	15	1.0%	1.00 [0.88, 1.13]
Folwaczny et al. 2001	19	23	20	22	0.3%	0.91 [0.72, 1.14]
Franco et al. 2006	27	27	17	17	1.7%	1.00 [0.91, 1.10]
Gonçalves et al. 2021	29	42	25	37	0.2%	1.02 [0.76, 1.38]
Hussainy et al. 2018	32	33	32	34	1.4%	1.03 [0.93, 1.14]
Jassal et al. 2018	90	90	92	92	11.9%	1.00 [0.98, 1.02]
Koc Vural et al. 2021	31	47	40	43	0.3%	0.71 [0.57, 0.88]
Matis et al. 1996	12	12	3	3	0.1%	1.00 [0.68, 1.47]
Miletić et al. 2020	142	142	142	142	14.9%	1.00 [0.99, 1.01]
Onal et al. 2005	24	24	13	13	1.1%	1.00 [0.89, 1.12]
Ozgünaltay et al. 2002	44	44	40	40	5.3%	1.00 [0.96, 1.05]
Perdigão et al. 2012	25	26	22	27	0.4%	1.18 [0.97, 1.43]
Pollington et al. 2008	28	30	28	30	0.8%	1.00 [0.87, 1.14]
Popescu et al. 2016	32	57	31	53	0.2%	0.96 [0.70, 1.33]
Santiago et al. 2010	33	33	26	26	3.0%	1.00 [0.94, 1.07]
Schwendicke et al. 2021	41	41	49	49	5.7%	1.00 [0.96, 1.04]
Türkün et al. 2008	50	50	50	50	6.7%	1.00 [0.96, 1.04]
van Dijken et al. 2019	82	82	82	82	11.0%	1.00 [0.98, 1.02]
Wafaie et al. 2023	37	37	39	39	4.6%	1.00 [0.95, 1.05]
**Total (95% CI)**		**1208**		**1149**	**100.0%**	**1.00 [0.99, 1.01]**
Total events	1128		1093			

Heterogeneity: Tau² = 0.00; Chi² = 37.12, df = 26 (P = 0.07); I² = 30%
Test for overall effect: Z = 0.20 (P = 0.84)

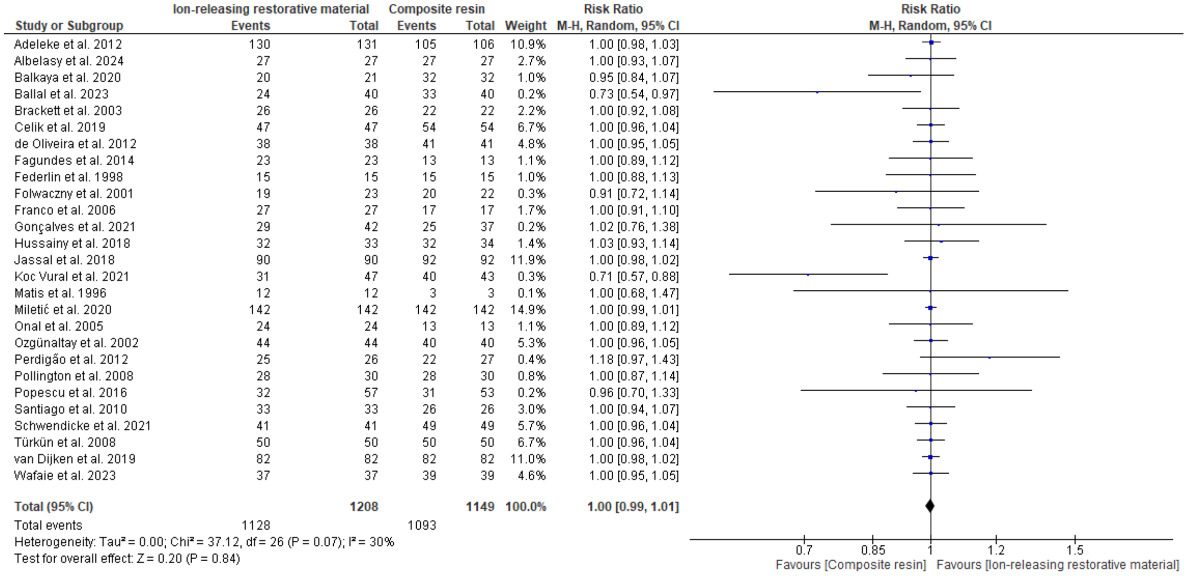

Figure S3. Adequate of marginal adaptation between IRR and CR in dental restorations

Study or Subgroup	Ion-releasing restorative material Events	Ion-releasing restorative material Total	Composite resin Events	Composite resin Total	Weight	Risk Ratio M-H, Random, 95% CI
Adeleke et al. 2012	131	131	105	106	11.9%	1.01 [0.99, 1.04]
Albelasy et al. 2024	27	27	27	27	1.5%	1.00 [0.93, 1.07]
Balkaya et al. 2020	20	21	32	32	0.5%	0.95 [0.84, 1.07]
Brackett et al. 2003	26	26	22	22	1.2%	1.00 [0.92, 1.08]
Celik et al. 2019	47	47	54	54	5.0%	1.00 [0.96, 1.04]
de Oliveira et al. 2012	38	38	41	41	3.1%	1.00 [0.95, 1.05]
Hussainy et al. 2018	32	33	32	34	0.7%	1.03 [0.93, 1.14]
Jassal et al. 2018	90	90	92	92	16.2%	1.00 [0.98, 1.02]
Koc Vural et al. 2021	47	47	43	43	4.0%	1.00 [0.96, 1.04]
Matis et al. 1996	10	12	2	3	0.0%	1.25 [0.54, 2.89]
Miletić et al. 2020	142	142	142	142	39.3%	1.00 [0.99, 1.01]
Ozgünaltay et al. 2002	44	44	40	40	3.5%	1.00 [0.96, 1.05]
Perdigão et al. 2012	26	26	23	27	0.3%	1.17 [0.99, 1.39]
Pollington et al. 2008	24	30	25	30	0.1%	0.96 [0.76, 1.22]
Popescu et al. 2016	32	57	30	53	0.1%	0.99 [0.71, 1.38]
Saghir et al. 2023	23	30	21	30	0.1%	1.10 [0.81, 1.49]
Schwendicke et al. 2021	40	41	49	49	1.8%	0.97 [0.91, 1.04]
Toz-Akalin et al. 2023	28	28	29	29	1.6%	1.00 [0.94, 1.07]
Türkün et al. 2008	50	50	50	50	5.0%	1.00 [0.96, 1.04]
van Dijken et al. 2019	74	82	81	82	1.3%	0.91 [0.85, 0.98]
Wafaie et al. 2023	37	37	39	39	2.9%	1.00 [0.95, 1.05]
**Total (95% CI)**		**1039**		**1025**	**100.0%**	**1.00 [0.99, 1.01]**
Total events	988		979			

Heterogeneity: Tau² = 0.00; Chi² = 12.15, df = 20 (P = 0.91); I² = 0%
Test for overall effect: Z = 0.01 (P = 0.99)

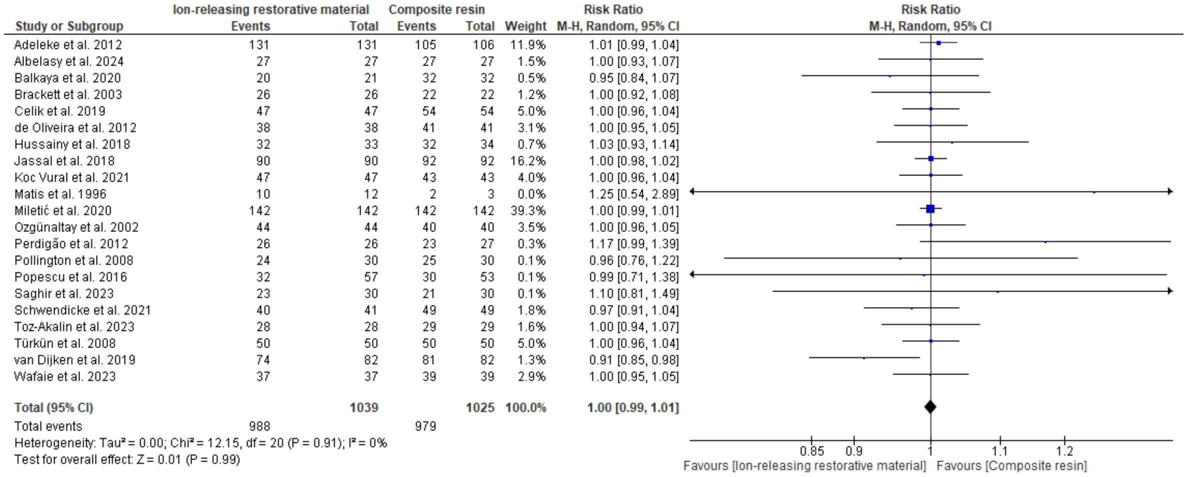

Figure S4. Adequate marginal or tooth integrity between IRR and CR in dental restorations

Study or Subgroup	Ion-releasing restorative material Events	Ion-releasing restorative material Total	Composite resin Events	Composite resin Total	Weight	Risk Ratio M-H, Random, 95% CI
Albelasy et al. 2024	26	27	27	27	1.4%	0.96 [0.87, 1.07]
Ballal et al. 2023	23	40	31	40	0.1%	0.74 [0.54, 1.02]
Celik et al. 2019	47	47	54	54	9.4%	1.00 [0.96, 1.04]
Fagundes et al. 2014	20	23	9	13	0.1%	1.26 [0.85, 1.87]
Federlin et al. 1998	15	15	13	15	0.3%	1.15 [0.91, 1.44]
Folwaczny et al. 2001	17	23	20	22	0.2%	0.81 [0.62, 1.07]
Franco et al. 2006	23	27	13	17	0.1%	1.11 [0.82, 1.51]
Gonçalves et al. 2021	30	42	25	37	0.2%	1.06 [0.79, 1.42]
Miletić et al. 2020	143	143	143	143	75.1%	1.00 [0.99, 1.01]
Onal et al. 2005	24	24	13	13	1.1%	1.00 [0.89, 1.12]
Santiago et al. 2010	33	33	26	26	3.2%	1.00 [0.94, 1.07]
Toz-Akalin et al. 2023	28	28	29	29	3.1%	1.00 [0.94, 1.07]
Wafaie et al. 2023	39	39	39	39	5.7%	1.00 [0.95, 1.05]
**Total (95% CI)**		**511**		**475**	**100.0%**	**1.00 [0.99, 1.01]**
Total events	468		442			

Heterogeneity: Tau² = 0.00; Chi² = 11.91, df = 12 (P = 0.45); I² = 0%
Test for overall effect: Z = 0.08 (P = 0.94)

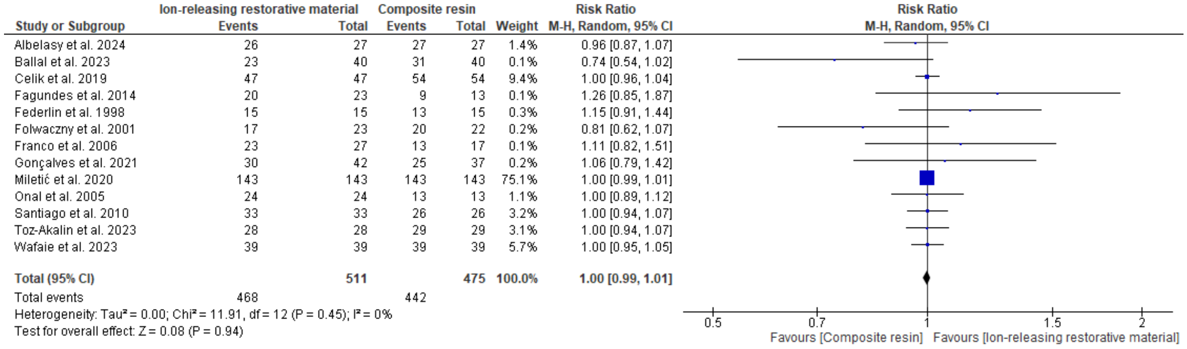

Figure S5. Adequate color or translucency between IRR and CR in dental restorations

Study or Subgroup	Ion-releasing restorative material Events	Ion-releasing restorative material Total	Composite resin Events	Composite resin Total	Weight	Risk Ratio M-H, Random, 95% CI
Balkaya et al. 2020	15	21	32	32	0.5%	0.72 [0.54, 0.94]
Brackett et al. 2003	26	26	22	22	5.1%	1.00 [0.92, 1.08]
Celik et al. 2019	47	47	54	54	14.0%	1.00 [0.96, 1.04]
de Oliveira et al. 2012	38	38	41	41	10.6%	1.00 [0.95, 1.05]
Federlin et al. 1998	15	15	15	15	2.3%	1.00 [0.88, 1.13]
Folwaczny et al. 2001	23	23	22	22	4.7%	1.00 [0.92, 1.09]
Gonçalves et al. 2021	27	42	29	37	0.5%	0.82 [0.62, 1.09]
Hussainy et al. 2018	32	33	32	34	3.3%	1.03 [0.93, 1.14]
Onal et al. 2005	24	24	13	13	2.7%	1.00 [0.89, 1.12]
Ozgünaltay et al. 2002	44	44	40	40	11.4%	1.00 [0.96, 1.05]
Perdigão et al. 2012	26	26	22	27	1.0%	1.22 [1.01, 1.48]
Popescu et al. 2016	50	57	44	53	1.5%	1.06 [0.90, 1.23]
Schwendicke et al. 2021	41	41	49	49	12.3%	1.00 [0.96, 1.04]
Toz-Akalin et al. 2023	28	28	29	29	6.8%	1.00 [0.94, 1.07]
Türkün et al. 2008	50	50	47	50	5.2%	1.06 [0.98, 1.15]
van Dijken et al. 2019	77	82	82	82	8.0%	0.94 [0.88, 1.00]
Wafaie et al. 2023	37	37	39	39	10.1%	1.00 [0.95, 1.05]
**Total (95% CI)**		**634**		**639**	**100.0%**	**1.00 [0.98, 1.02]**
Total events	600		612			

Heterogeneity: Tau² = 0.00; Chi² = 20.50, df = 16 (P = 0.20); I² = 22%
Test for overall effect: Z = 0.06 (P = 0.95)

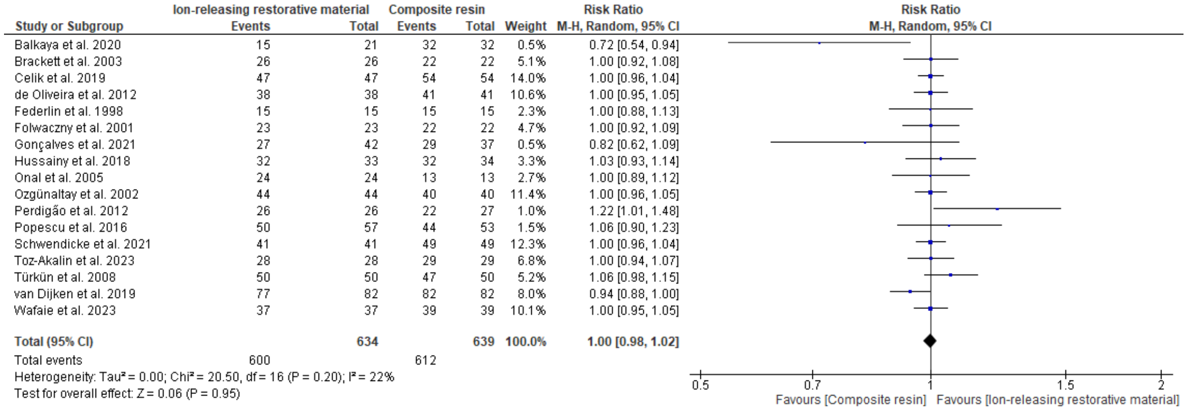

Figure S6. Proper surface texture or luster between IRR and CR in dental restorations

Study or Subgroup	Ion-releasing restorative material Events	Total	Composite resin Events	Total	Weight	Risk Ratio M-H, Random, 95% CI
Balkaya et al. 2020	19	21	32	32	2.4%	0.90 [0.77, 1.05]
Brackett et al. 2003	26	26	22	22	6.6%	1.00 [0.92, 1.08]
Celik et al. 2019	47	47	54	54	12.3%	1.00 [0.96, 1.04]
Federlin et al. 1998	15	15	15	15	3.5%	1.00 [0.88, 1.13]
Folwaczny et al. 2001	23	23	22	22	6.2%	1.00 [0.92, 1.09]
Gonçalves et al. 2021	34	42	35	37	2.2%	0.86 [0.73, 1.01]
Hussainy et al. 2018	32	33	32	34	4.7%	1.03 [0.93, 1.14]
Matis et al. 1996	12	12	3	3	0.5%	1.00 [0.68, 1.47]
Onal et al. 2005	24	24	13	13	4.0%	1.00 [0.89, 1.12]
Perdigão et al. 2012	11	26	25	27	0.3%	0.46 [0.29, 0.72]
Saghir et al. 2023	25	30	18	30	0.6%	1.39 [1.00, 1.94]
Schwendicke et al. 2021	41	41	49	49	11.5%	1.00 [0.96, 1.04]
Toz-Akalin et al. 2023	28	28	29	29	8.0%	1.00 [0.94, 1.07]
Türkün et al. 2008	50	50	50	50	12.3%	1.00 [0.96, 1.04]
van Dijken et al. 2019	82	82	82	82	14.7%	1.00 [0.98, 1.02]
Wafaie et al. 2023	37	37	39	39	10.3%	1.00 [0.95, 1.05]
**Total (95% CI)**		**537**		**538**	**100.0%**	**0.99 [0.97, 1.02]**
Total events	506		520			

Heterogeneity: Tau² = 0.00; Chi² = 31.43, df = 15 (P = 0.008); I² = 52%
Test for overall effect: Z = 0.38 (P = 0.70)

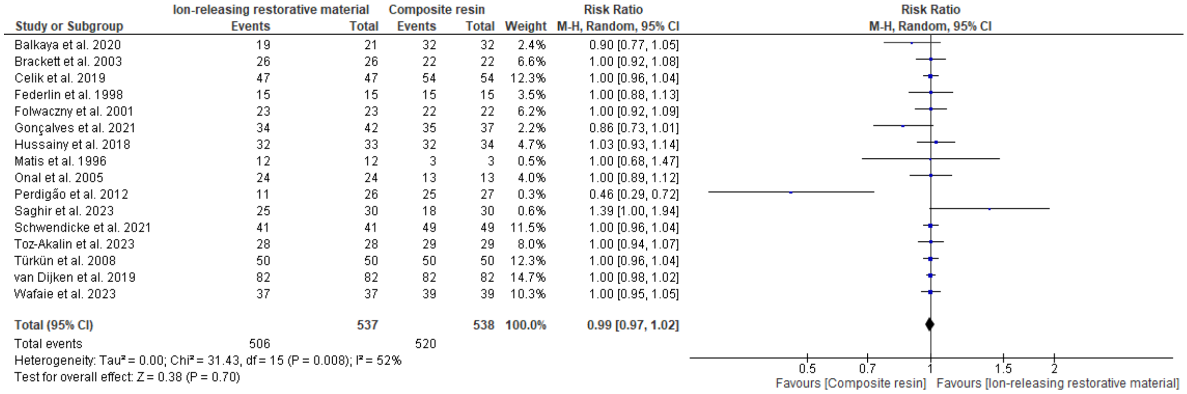

Figure S7. Proper surface staining between IRR and CR in dental restorations

Study or Subgroup	Ion-releasing restorative material Events	Total	Composite resin Events	Total	Weight	Risk Ratio M-H, Random, 95% CI
Celik et al. 2019	47	47	54	54	9.2%	1.00 [0.96, 1.04]
Gonçalves et al. 2021	40	42	35	37	1.3%	1.01 [0.91, 1.12]
Matis et al. 1996	11	12	3	3	0.1%	1.01 [0.66, 1.54]
Miletić et al. 2020	143	143	143	143	73.7%	1.00 [0.99, 1.01]
Schwendicke et al. 2021	41	41	49	49	7.3%	1.00 [0.96, 1.04]
Toz-Akalin et al. 2023	28	28	29	29	3.0%	1.00 [0.94, 1.07]
Wafaie et al. 2023	37	37	39	39	5.3%	1.00 [0.95, 1.05]
**Total (95% CI)**		**350**		**354**	**100.0%**	**1.00 [0.99, 1.01]**
Total events	347		352			

Heterogeneity: Tau² = 0.00; Chi² = 0.04, df = 6 (P = 1.00); I² = 0%
Test for overall effect: Z = 0.02 (P = 0.99)

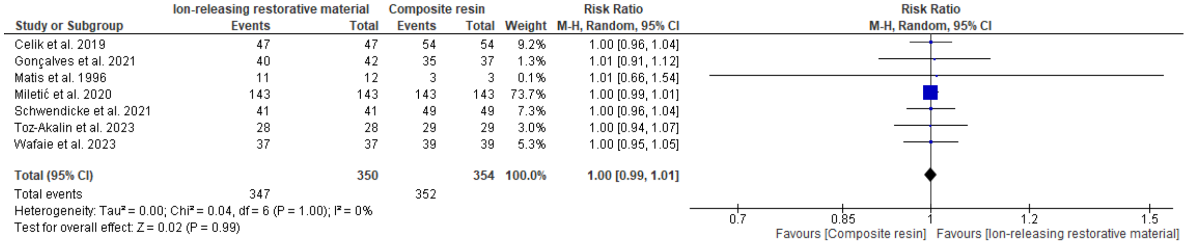

Figure S8. Retention between IRR and CR in dental restorations

Study or Subgroup	Ion-releasing restorative material Events	Total	Composite resin Events	Total	Weight	Risk Ratio M-H, Random, 95% CI
Adeleke et al. 2012	131	144	106	143	4.3%	1.23 [1.10, 1.37]
Albelasy et al. 2024	26	27	27	27	4.5%	0.96 [0.87, 1.07]
Balkaya et al. 2020	15	21	32	32	1.6%	0.72 [0.54, 0.94]
Ballal et al. 2023	24	40	33	40	1.5%	0.73 [0.54, 0.97]
Brackett et al. 2003	26	27	22	27	2.5%	1.18 [0.97, 1.44]
Celik et al. 2019	47	49	54	54	5.3%	0.96 [0.90, 1.03]
de Oliveira et al. 2012	38	40	41	41	4.9%	0.95 [0.87, 1.03]
Fagundes et al. 2014	23	26	13	25	0.9%	1.70 [1.14, 2.54]
Folwaczny et al. 2001	23	26	22	23	3.1%	0.92 [0.79, 1.09]
Franco et al. 2006	27	28	27	33	2.8%	1.18 [0.99, 1.41]
Gonçalves et al. 2021	42	43	37	42	4.0%	1.11 [0.98, 1.25]
Hussainy et al. 2018	32	33	32	34	4.4%	1.03 [0.93, 1.14]
Jassal et al. 2018	90	98	92	98	5.1%	0.98 [0.91, 1.06]
Koc Vural et al. 2021	47	51	43	51	3.5%	1.09 [0.95, 1.26]
Matis et al. 1996	12	18	3	18	0.1%	4.00 [1.35, 11.82]
Miletić et al. 2020	143	143	141	143	6.2%	1.01 [0.99, 1.04]
Onal et al. 2005	24	24	13	18	1.4%	1.38 [1.03, 1.85]
Ozgünaltay et al. 2002	44	45	40	42	5.0%	1.03 [0.95, 1.11]
Perdigão et al. 2012	26	26	25	27	3.9%	1.08 [0.95, 1.22]
Pollington et al. 2008	26	30	26	30	2.5%	1.00 [0.82, 1.22]
Popescu et al. 2016	54	57	53	57	4.7%	1.02 [0.93, 1.12]
Saghir et al. 2023	28	30	19	30	1.5%	1.47 [1.10, 1.97]
Santiago et al. 2010	33	33	26	33	2.7%	1.26 [1.05, 1.52]
Schwendicke et al. 2021	39	41	49	49	5.0%	0.95 [0.88, 1.03]
Toz-Akalin et al. 2023	28	29	29	29	4.7%	0.97 [0.88, 1.06]
Türkün et al. 2008	48	50	50	50	5.3%	0.96 [0.90, 1.03]
van Dijken et al. 2019	62	82	80	82	3.8%	0.78 [0.68, 0.88]
Wafaie et al. 2023	37	39	39	39	4.8%	0.95 [0.87, 1.04]
**Total (95% CI)**		**1300**		**1317**	**100.0%**	**1.02 [0.98, 1.06]**
Total events	1195		1174			

Heterogeneity: Tau² = 0.01; Chi² = 103.38, df = 27 (P < 0.00001); I² = 74%
Test for overall effect: Z = 0.82 (P = 0.41)

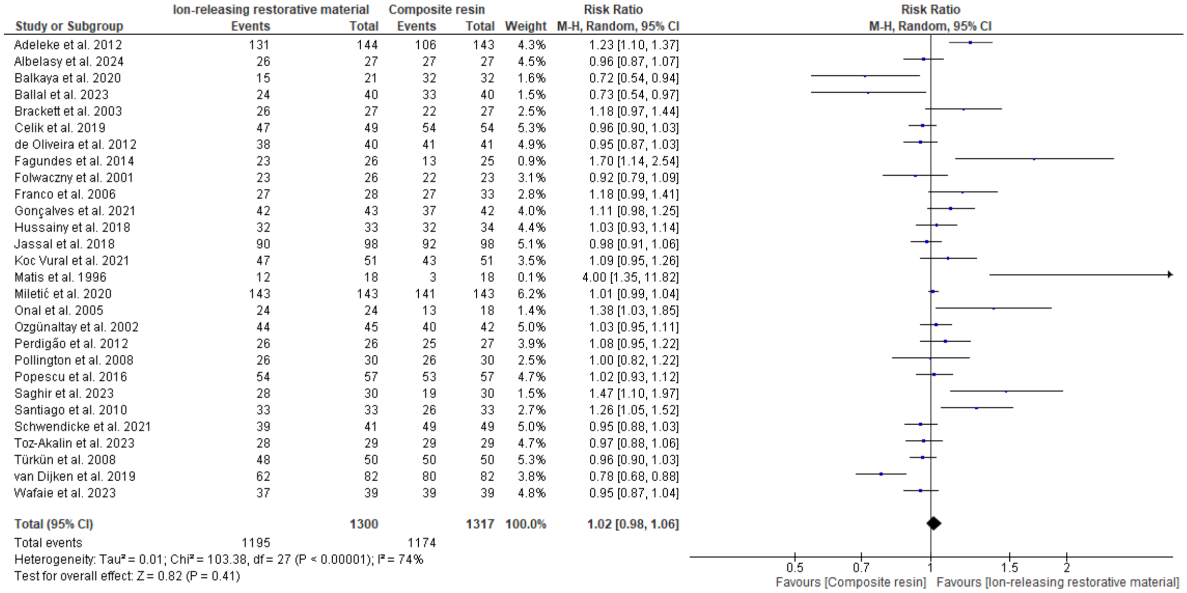

Figure S9. Absence of wear between IRR and CR in dental restorations

	Ion-releasing restorative material		Composite resin			Risk Ratio
Study or Subgroup	Events	Total	Events	Total	Weight	M-H, Random, 95% CI
Adeleke et al. 2012	131	131	106	106	32.7%	1.00 [0.98, 1.02]
Celik et al. 2019	47	47	54	54	6.2%	1.00 [0.96, 1.04]
Gonçalves et al. 2021	39	42	36	37	0.9%	0.95 [0.86, 1.05]
Matis et al. 1996	9	12	3	3	0.0%	0.84 [0.51, 1.37]
Miletić et al. 2020	143	143	143	143	49.1%	1.00 [0.99, 1.01]
Perdigão et al. 2012	26	26	25	27	0.6%	1.08 [0.95, 1.22]
Schwendicke et al. 2021	41	41	49	49	4.9%	1.00 [0.96, 1.04]
Toz-Akalin et al. 2023	28	28	29	29	2.0%	1.00 [0.94, 1.07]
Wafaie et al. 2023	37	37	39	39	3.6%	1.00 [0.95, 1.05]
**Total (95% CI)**		**507**		**487**	**100.0%**	**1.00 [0.99, 1.01]**
Total events	501		484			

Heterogeneity: Tau² = 0.00; Chi² = 2.76, df = 8 (P = 0.95); I² = 0%
Test for overall effect: Z = 0.02 (P = 0.99)

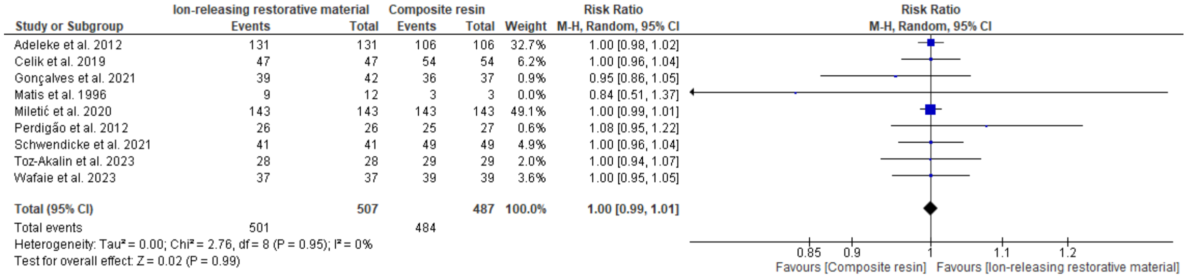

Figure S10. Proper anatomic form between IRR and CR in dental restorations

	Ion-releasing restorative material		Composite resin			Risk Ratio
Study or Subgroup	Events	Total	Events	Total	Weight	M-H, Random, 95% CI
Balkaya et al. 2020	20	21	32	32	4.0%	0.95 [0.84, 1.07]
Ballal et al. 2023	22	40	32	40	0.9%	0.69 [0.50, 0.95]
Brackett et al. 2003	26	26	22	22	6.0%	1.00 [0.92, 1.08]
Celik et al. 2019	45	47	54	54	6.5%	0.96 [0.89, 1.03]
de Oliveira et al. 2012	38	38	41	41	7.8%	1.00 [0.95, 1.05]
Fagundes et al. 2014	21	23	12	13	2.0%	0.99 [0.81, 1.21]
Federlin et al. 1998	15	15	15	15	3.9%	1.00 [0.88, 1.13]
Folwaczny et al. 2001	14	23	22	22	0.8%	0.62 [0.44, 0.86]
Franco et al. 2006	23	27	15	17	1.5%	0.97 [0.76, 1.22]
Gonçalves et al. 2021	40	42	36	37	5.6%	0.98 [0.90, 1.07]
Matis et al. 1996	8	12	3	3	0.3%	0.75 [0.43, 1.28]
Miletić et al. 2020	143	143	143	143	9.5%	1.00 [0.99, 1.01]
Onal et al. 2005	24	24	13	13	4.2%	1.00 [0.89, 1.12]
Ozgünaltay et al. 2002	44	44	40	40	8.0%	1.00 [0.96, 1.05]
Pollington et al. 2008	27	30	29	30	3.4%	0.93 [0.81, 1.07]
Popescu et al. 2016	57	57	53	53	8.6%	1.00 [0.97, 1.04]
Santiago et al. 2010	33	33	25	26	4.9%	1.04 [0.94, 1.15]
Schwendicke et al. 2021	41	41	49	49	8.2%	1.00 [0.96, 1.04]
van Dijken et al. 2019	74	82	81	82	6.2%	0.91 [0.85, 0.98]
Wafaie et al. 2023	37	37	39	39	7.7%	1.00 [0.95, 1.05]
**Total (95% CI)**		**805**		**771**	**100.0%**	**0.98 [0.95, 1.01]**
Total events	752		756			

Heterogeneity: Tau² = 0.00; Chi² = 78.25, df = 19 (P < 0.00001); I² = 76%
Test for overall effect: Z = 1.33 (P = 0.18)

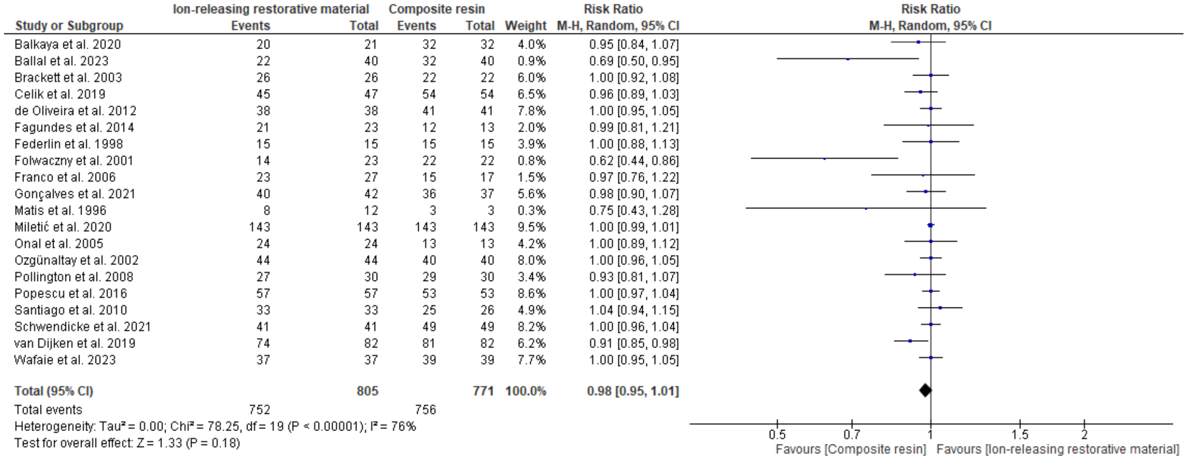

Figure S11. Absence of sensibility between IRR and CR in dental restorations

	Ion-releasing restorative material		Composite resin			Risk Ratio
Study or Subgroup	Events	Total	Events	Total	Weight	M-H, Random, 95% CI
Adeleke et al. 2012	131	131	106	106	24.3%	1.00 [0.98, 1.02]
Albelasy et al. 2024	27	27	27	27	1.4%	1.00 [0.93, 1.07]
Balkaya et al. 2020	21	21	32	32	1.2%	1.00 [0.93, 1.08]
Celik et al. 2019	47	47	54	54	4.6%	1.00 [0.96, 1.04]
Hussainy et al. 2018	32	33	32	34	0.6%	1.03 [0.93, 1.14]
Jassal et al. 2018	90	90	92	92	14.9%	1.00 [0.98, 1.02]
Koc Vural et al. 2021	47	47	43	43	3.7%	1.00 [0.96, 1.04]
Miletić et al. 2020	143	143	143	143	36.6%	1.00 [0.99, 1.01]
Perdigão et al. 2012	25	26	24	27	0.3%	1.08 [0.93, 1.26]
Schwendicke et al. 2021	41	41	49	49	3.6%	1.00 [0.96, 1.04]
Toz-Akalin et al. 2023	28	28	29	29	1.5%	1.00 [0.94, 1.07]
Türkün et al. 2008	50	50	50	50	4.6%	1.00 [0.96, 1.04]
Wafaie et al. 2023	39	39	39	39	2.8%	1.00 [0.95, 1.05]
**Total (95% CI)**		**723**		**725**	**100.0%**	**1.00 [0.99, 1.01]**
Total events	721		720			

Heterogeneity: Tau² = 0.00; Chi² = 2.03, df = 12 (P = 1.00); I² = 0%
Test for overall effect: Z = 0.10 (P = 0.92)

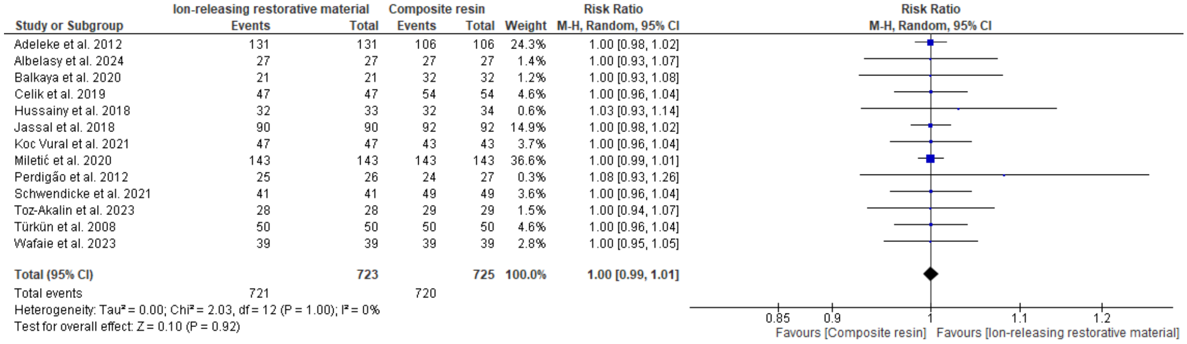

Figure S12. Adequate periodontal tissue between IRR and CR in dental restorations

	Ion-releasing restorative material		Composite resin			Risk Ratio
Study or Subgroup	Events	Total	Events	Total	Weight	M-H, Random, 95% CI
Celik et al. 2019	47	47	52	54	3.4%	1.04 [0.97, 1.11]
Gonçalves et al. 2021	42	42	36	37	2.8%	1.03 [0.96, 1.11]
Miletić et al. 2020	144	144	144	144	78.3%	1.00 [0.99, 1.01]
Türkün et al. 2008	50	50	50	50	9.6%	1.00 [0.96, 1.04]
Wafaie et al. 2023	39	39	39	39	5.9%	1.00 [0.95, 1.05]
**Total (95% CI)**		**322**		**324**	**100.0%**	**1.00 [0.99, 1.01]**
Total events	322		321			

Heterogeneity: Tau² = 0.00; Chi² = 3.00, df = 4 (P = 0.56); I² = 0%
Test for overall effect: Z = 0.33 (P = 0.74)

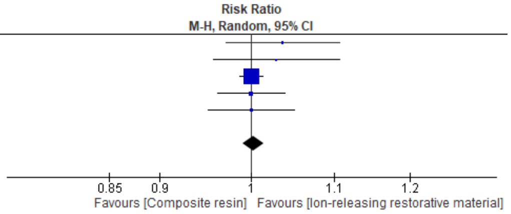

Figure S13. Absence of secondary caries or erosion or abfraction between IRR and CR in dental restorations by restorative material

	Ion-releasing restorative material		Composite resin			Risk Ratio
Study or Subgroup	Events	Total	Events	Total	Weight	M-H, Random, 95% CI
**3.1.1 RMGIC vs CR**						
Adeleke et al. 2012	117	117	72	72	5.1%	1.00 [0.98, 1.02]
Ballal et al. 2023	40	40	40	40	4.7%	1.00 [0.95, 1.05]
Brackett et al. 2003	26	26	22	22	4.1%	1.00 [0.92, 1.08]
de Oliveira et al. 2012	38	38	41	41	4.7%	1.00 [0.95, 1.05]
Federlin et al. 1998	15	15	15	15	3.1%	1.00 [0.88, 1.13]
Franco et al. 2006	27	27	15	17	2.0%	1.14 [0.94, 1.38]
Gonçalves et al. 2021	42	42	37	37	4.7%	1.00 [0.95, 1.05]
Jassal et al. 2018	90	90	92	92	5.1%	1.00 [0.98, 1.02]
Koc Vural et al. 2021	47	47	43	43	4.8%	1.00 [0.96, 1.04]
Onal et al. 2005	24	24	13	13	3.3%	1.00 [0.89, 1.12]
Ozgünaltay et al. 2002	44	44	40	40	4.7%	1.00 [0.96, 1.05]
Perdigão et al. 2012	26	26	25	27	3.1%	1.08 [0.95, 1.22]
Popescu et al. 2016	57	57	53	53	4.9%	1.00 [0.97, 1.04]
Santiago et al. 2010	33	33	26	26	4.4%	1.00 [0.94, 1.07]
van Dijken et al. 2019	3	82	82	82	0.1%	0.04 [0.02, 0.12]
**Subtotal (95% CI)**		**708**		**620**	**58.8%**	**1.00 [0.92, 1.07]**
Total events	629		616			
Heterogeneity: Tau² = 0.02; Chi² = 481.33, df = 14 (P < 0.00001); I² = 97%						
Test for overall effect: Z = 0.11 (P = 0.91)						
**3.1.2 HVGIC vs CR**						
Balkaya et al. 2020	21	21	32	32	4.1%	1.00 [0.93, 1.08]
Celik et al. 2019	47	47	54	54	4.9%	1.00 [0.96, 1.04]
Fagundes et al. 2014	23	23	12	13	2.0%	1.10 [0.91, 1.33]
Miletić et al. 2020	143	143	143	143	5.1%	1.00 [0.99, 1.01]
Schwendicke et al. 2021	41	41	49	49	4.8%	1.00 [0.96, 1.04]
Wafaie et al. 2023	37	37	39	39	4.7%	1.00 [0.95, 1.05]
**Subtotal (95% CI)**		**312**		**330**	**25.6%**	**1.00 [0.99, 1.01]**
Total events	312		329			
Heterogeneity: Tau² = 0.00; Chi² = 1.43, df = 5 (P = 0.92); I² = 0%						
Test for overall effect: Z = 0.06 (P = 0.95)						
**3.1.3 PMCR vs CR**						
Federlin et al. 1998	15	15	15	15	3.1%	1.00 [0.88, 1.13]
Onal et al. 2005	21	21	13	13	3.2%	1.00 [0.89, 1.13]
Pollington et al. 2008	30	30	30	30	4.4%	1.00 [0.94, 1.07]
Türkün et al. 2008	50	50	50	50	4.9%	1.00 [0.96, 1.04]
**Subtotal (95% CI)**		**116**		**108**	**15.6%**	**1.00 [0.97, 1.03]**
Total events	116		108			
Heterogeneity: Tau² = 0.00; Chi² = 0.00, df = 3 (P = 1.00); I² = 0%						
Test for overall effect: Z = 0.00 (P = 1.00)						
**Total (95% CI)**		**1136**		**1058**	**100.0%**	**1.00 [0.97, 1.04]**
Total events	1057		1053			
Heterogeneity: Tau² = 0.01; Chi² = 340.85, df = 24 (P < 0.00001); I² = 93%						
Test for overall effect: Z = 0.18 (P = 0.85)						
Test for subgroup differences: Chi² = 0.01, df = 2 (P = 0.99), I² = 0%						

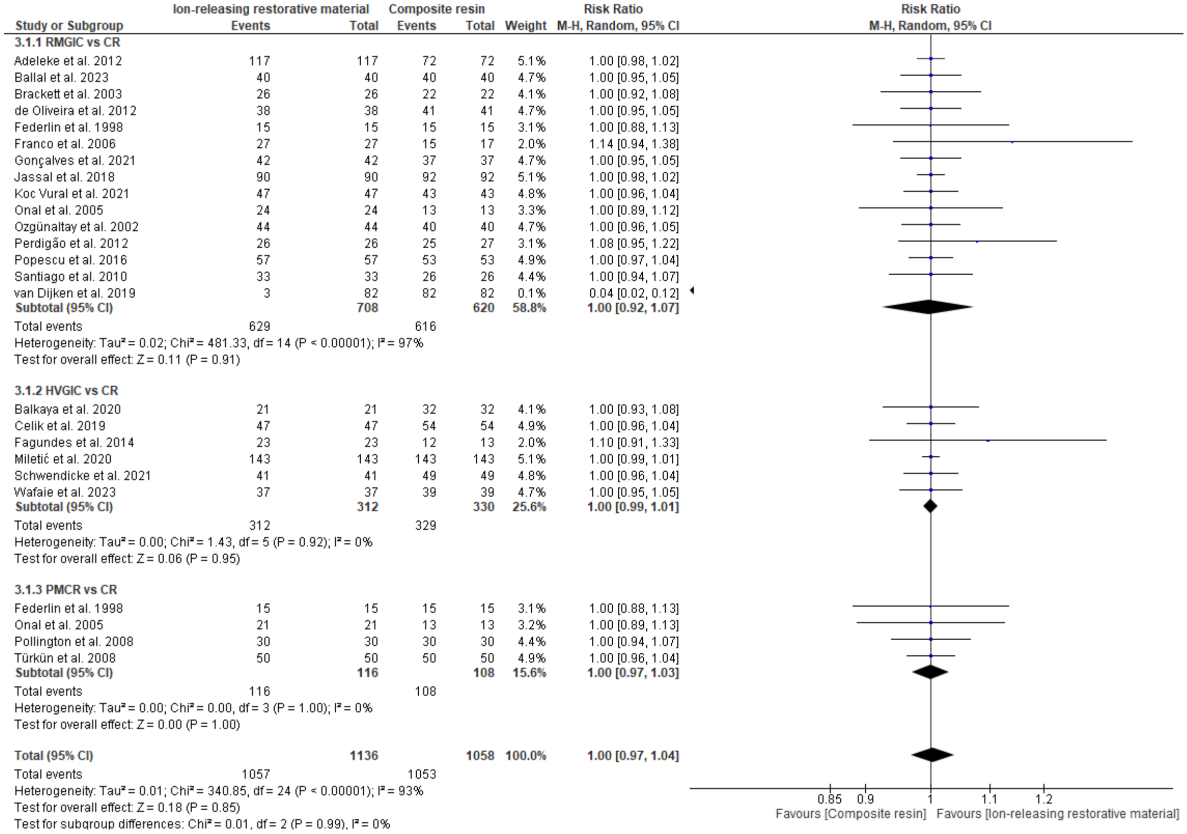

Figure S14. Absence of marginal discoloration between IRR and CR in dental restorations by restorative material

Study or Subgroup	Ion-releasing restorative material Events	Total	Composite resin Events	Total	Weight	Risk Ratio M-H, Random, 95% CI
**3.2.1 RMGIC vs CR**						
Adeleke et al. 2012	130	131	105	106	11.7%	1.00 [0.98, 1.03]
Ballal et al. 2023	24	40	33	40	0.1%	0.73 [0.54, 0.97]
Brackett et al. 2003	26	26	22	22	1.7%	1.00 [0.92, 1.08]
de Oliveira et al. 2012	38	38	41	41	4.2%	1.00 [0.95, 1.05]
Federlin et al. 1998	15	15	15	15	0.7%	1.00 [0.88, 1.13]
Folwaczny et al. 2001	19	23	20	22	0.2%	0.91 [0.72, 1.14]
Franco et al. 2006	27	27	17	17	1.3%	1.00 [0.91, 1.10]
Gonçalves et al. 2021	29	42	25	37	0.1%	1.02 [0.76, 1.38]
Hussainy et al. 2018	32	33	32	34	1.1%	1.03 [0.93, 1.14]
Jassal et al. 2018	90	90	92	92	13.2%	1.00 [0.98, 1.02]
Koc Vural et al. 2021	31	47	40	43	0.2%	0.71 [0.57, 0.88]
Onal et al. 2005	24	24	13	13	0.9%	1.00 [0.89, 1.12]
Ozgünaltay et al. 2002	44	44	40	40	4.6%	1.00 [0.96, 1.05]
Perdigão et al. 2012	25	26	22	27	0.3%	1.18 [0.97, 1.43]
Popescu et al. 2016	32	57	31	53	0.1%	0.96 [0.70, 1.33]
Santiago et al. 2010	33	33	26	26	2.4%	1.00 [0.94, 1.07]
van Dijken et al. 2019	82	82	82	82	11.8%	1.00 [0.98, 1.02]
**Subtotal (95% CI)**		**778**		**710**	**54.6%**	**1.00 [0.97, 1.02]**
Total events	701		656			
Heterogeneity: Tau² = 0.00; Chi² = 38.95, df = 16 (P = 0.001); I² = 59%						
Test for overall effect: Z = 0.28 (P = 0.78)						
**3.2.2 HVGIC vs CR**						
Balkaya et al. 2020	20	21	32	32	0.8%	0.95 [0.84, 1.07]
Celik et al. 2019	47	47	54	54	6.1%	1.00 [0.96, 1.04]
Fagundes et al. 2014	23	23	13	13	0.8%	1.00 [0.89, 1.12]
Miletić et al. 2020	142	142	142	142	18.8%	1.00 [0.99, 1.01]
Schwendicke et al. 2021	41	41	49	49	5.1%	1.00 [0.96, 1.04]
Wafaie et al. 2023	37	37	39	39	3.9%	1.00 [0.95, 1.05]
**Subtotal (95% CI)**		**311**		**329**	**35.5%**	**1.00 [0.99, 1.01]**
Total events	310		329			
Heterogeneity: Tau² = 0.00; Chi² = 1.20, df = 5 (P = 0.94); I² = 0%						
Test for overall effect: Z = 0.09 (P = 0.93)						
**3.2.3 PMCR vs CR**						
Federlin et al. 1998	15	15	15	15	0.7%	1.00 [0.88, 1.13]
Folwaczny et al. 2001	40	43	20	22	0.5%	1.02 [0.88, 1.20]
Hussainy et al. 2018	33	34	32	34	1.1%	1.03 [0.93, 1.14]
Onal et al. 2005	21	21	13	13	0.8%	1.00 [0.89, 1.13]
Pollington et al. 2008	28	30	28	30	0.6%	1.00 [0.87, 1.14]
Türkün et al. 2008	50	50	50	50	6.1%	1.00 [0.96, 1.04]
**Subtotal (95% CI)**		**193**		**164**	**9.8%**	**1.00 [0.97, 1.04]**
Total events	187		158			
Heterogeneity: Tau² = 0.00; Chi² = 0.49, df = 5 (P = 0.99); I² = 0%						
Test for overall effect: Z = 0.24 (P = 0.81)						
**Total (95% CI)**		**1282**		**1203**	**100.0%**	**1.00 [0.99, 1.01]**
Total events	1198		1143			
Heterogeneity: Tau² = 0.00; Chi² = 33.59, df = 28 (P = 0.21); I² = 17%						
Test for overall effect: Z = 0.08 (P = 0.93)						
Test for subgroup differences: Chi² = 0.13, df = 2 (P = 0.94), I² = 0%						

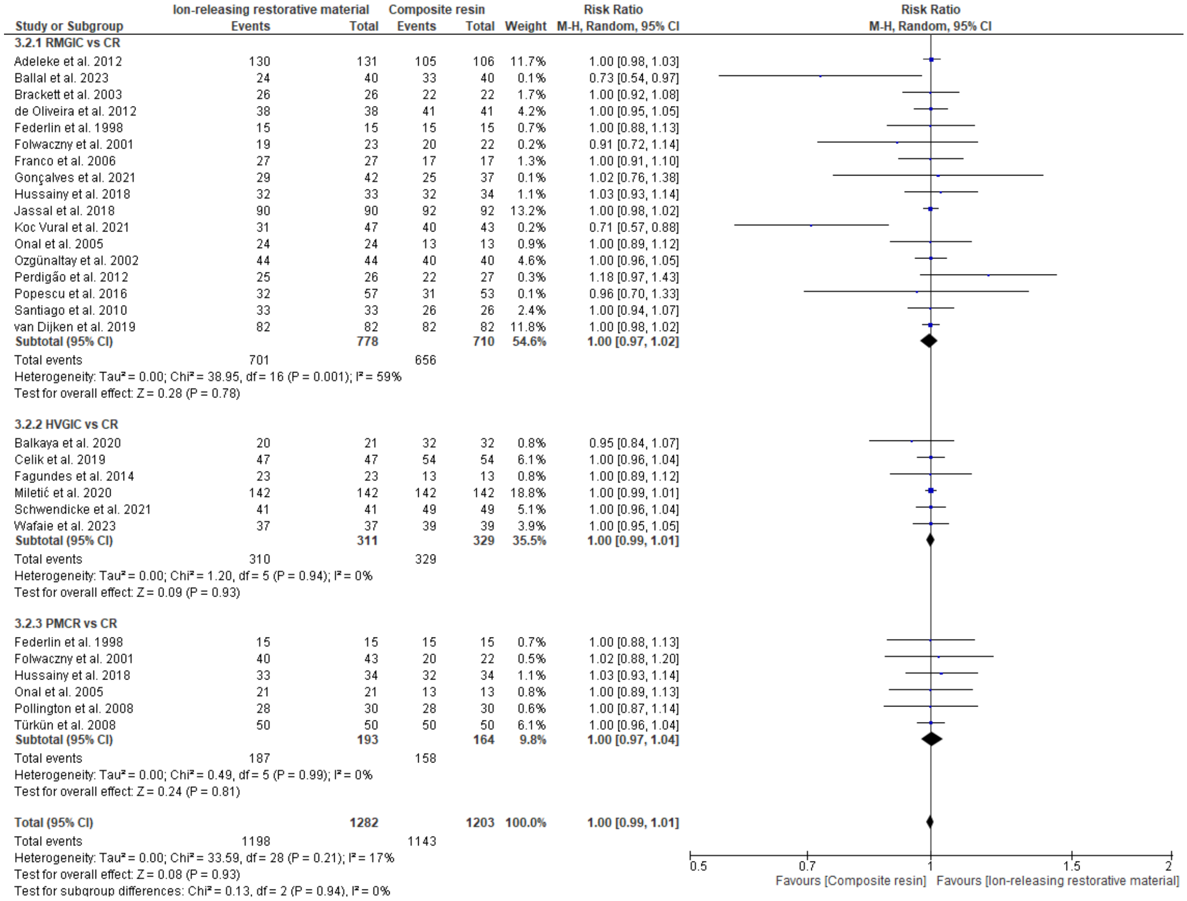

Figure S15. Adequate of marginal adaptation between IRR and CR in dental restorations by restorative material

Study or Subgroup	Ion-releasing restorative material Events	Total	Composite resin Events	Total	Weight	Risk Ratio M-H, Random, 95% CI
**3.3.1 RMGIC vs CR**						
Adeleke et al. 2012	131	131	105	106	12.2%	1.01 [0.99, 1.04]
Brackett et al. 2003	26	26	22	22	1.2%	1.00 [0.92, 1.08]
de Oliveira et al. 2012	38	38	41	41	3.2%	1.00 [0.95, 1.05]
Hussainy et al. 2018	32	33	32	34	0.7%	1.03 [0.93, 1.14]
Jassal et al. 2018	90	90	92	92	16.6%	1.00 [0.98, 1.02]
Koc Vural et al. 2021	47	47	43	43	4.1%	1.00 [0.96, 1.04]
Ozgünaltay et al. 2002	44	44	40	40	3.6%	1.00 [0.96, 1.05]
Perdigão et al. 2012	26	26	23	27	0.3%	1.17 [0.99, 1.39]
Popescu et al. 2016	32	57	30	53	0.1%	0.99 [0.71, 1.38]
Saghir et al. 2023	23	30	21	30	0.1%	1.10 [0.81, 1.49]
van Dijken et al. 2019	74	82	81	82	1.3%	0.91 [0.85, 0.98]
**Subtotal (95% CI)**		**604**		**570**	**43.4%**	**1.00 [0.99, 1.02]**
Total events	563		530			
Heterogeneity: Tau² = 0.00; Chi² = 10.07, df = 10 (P = 0.43); I² = 1%						
Test for overall effect: Z = 0.24 (P = 0.81)						
**3.3.2 HVGIC vs CR**						
Balkaya et al. 2020	20	21	32	32	0.5%	0.95 [0.84, 1.07]
Celik et al. 2019	47	47	54	54	5.1%	1.00 [0.96, 1.04]
Miletić et al. 2020	142	142	142	142	40.3%	1.00 [0.99, 1.01]
Schwendicke et al. 2021	40	41	49	49	1.8%	0.97 [0.91, 1.04]
Wafaie et al. 2023	37	37	39	39	3.0%	1.00 [0.95, 1.05]
**Subtotal (95% CI)**		**288**		**316**	**50.7%**	**1.00 [0.99, 1.01]**
Total events	286		316			
Heterogeneity: Tau² = 0.00; Chi² = 2.65, df = 4 (P = 0.62); I² = 0%						
Test for overall effect: Z = 0.24 (P = 0.81)						
**3.3.3 PMCR vs CR**						
Hussainy et al. 2018	33	34	32	34	0.7%	1.03 [0.93, 1.14]
Pollington et al. 2008	24	30	25	30	0.1%	0.96 [0.76, 1.22]
Türkün et al. 2008	50	50	50	50	5.1%	1.00 [0.96, 1.04]
**Subtotal (95% CI)**		**114**		**114**	**5.9%**	**1.00 [0.97, 1.04]**
Total events	107		107			
Heterogeneity: Tau² = 0.00; Chi² = 0.46, df = 2 (P = 0.80); I² = 0%						
Test for overall effect: Z = 0.16 (P = 0.88)						
**Total (95% CI)**		**1006**		**1000**	**100.0%**	**1.00 [0.99, 1.01]**
Total events	956		953			
Heterogeneity: Tau² = 0.00; Chi² = 12.23, df = 18 (P = 0.84); I² = 0%						
Test for overall effect: Z = 0.03 (P = 0.98)						
Test for subgroup differences: Chi² = 0.14, df = 2 (P = 0.93), I² = 0%						

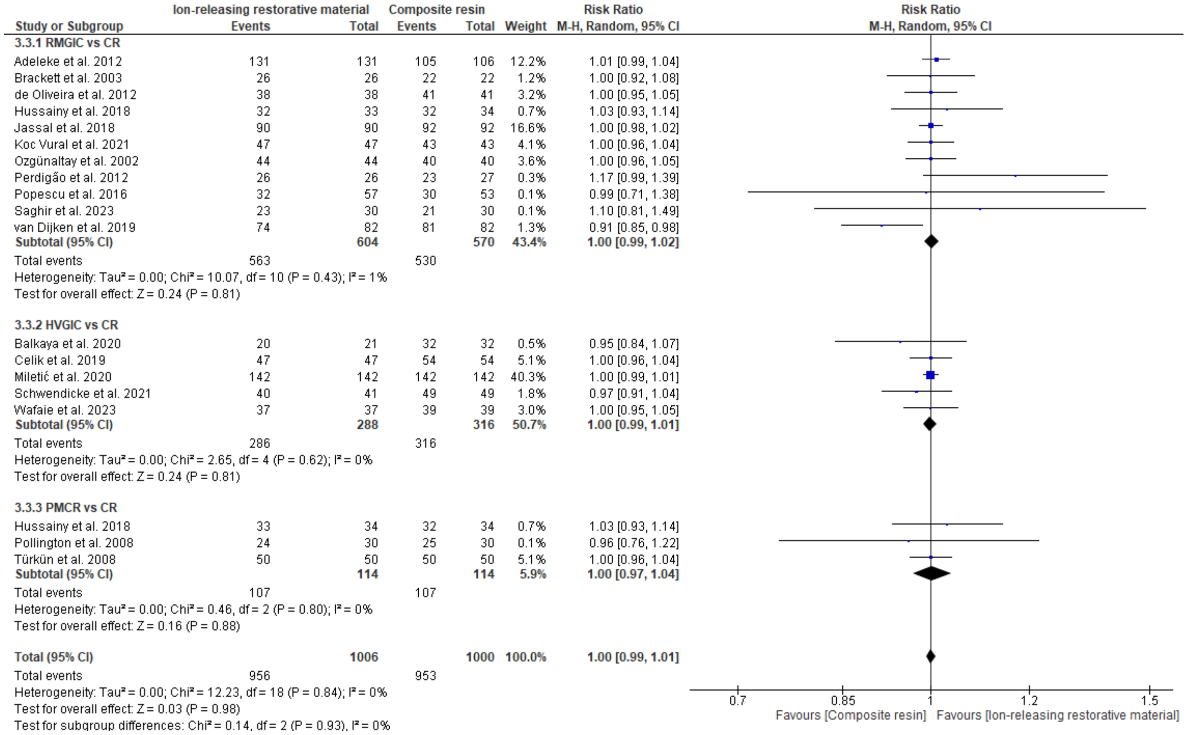

Figure S16. Adequate marginal or tooth integrity between IRR and CR in dental restorations by restorative material

Study or Subgroup	Ion-releasing restorative material Events	Total	Composite resin Events	Total	Weight	Risk Ratio M-H, Random, 95% CI
**3.4.1 RMGIC vs CR**						
Ballal et al. 2023	23	40	31	40	0.1%	0.74 [0.54, 1.02]
Federlin et al. 1998	15	15	13	15	0.3%	1.15 [0.91, 1.44]
Folwaczny et al. 2001	17	23	20	22	0.2%	0.81 [0.62, 1.07]
Franco et al. 2006	23	27	13	17	0.2%	1.11 [0.82, 1.51]
Gonçalves et al. 2021	30	42	25	37	0.2%	1.06 [0.79, 1.42]
Onal et al. 2005	24	24	13	13	1.1%	1.00 [0.89, 1.12]
Santiago et al. 2010	33	33	26	26	3.3%	1.00 [0.94, 1.07]
**Subtotal (95% CI)**		**204**		**170**	**5.3%**	**0.99 [0.91, 1.08]**
Total events	165		141			
Heterogeneity: Tau² = 0.00; Chi² = 9.81, df = 6 (P = 0.13); I² = 39%						
Test for overall effect: Z = 0.22 (P = 0.82)						
**3.4.2 HVGIC vs CR**						
Celik et al. 2019	47	47	54	54	9.7%	1.00 [0.96, 1.04]
Fagundes et al. 2014	20	23	9	13	0.1%	1.26 [0.85, 1.87]
Miletić et al. 2020	143	143	143	143	77.3%	1.00 [0.99, 1.01]
Wafaie et al. 2023	39	39	39	39	5.9%	1.00 [0.95, 1.05]
**Subtotal (95% CI)**		**252**		**249**	**93.0%**	**1.00 [0.98, 1.03]**
Total events	249		245			
Heterogeneity: Tau² = 0.00; Chi² = 4.68, df = 3 (P = 0.20); I² = 36%						
Test for overall effect: Z = 0.07 (P = 0.94)						
**3.4.3 PMCR vs CR**						
Federlin et al. 1998	15	15	13	15	0.3%	1.15 [0.91, 1.44]
Folwaczny et al. 2001	36	43	20	22	0.4%	0.92 [0.76, 1.11]
Onal et al. 2005	21	21	13	13	1.0%	1.00 [0.89, 1.13]
**Subtotal (95% CI)**		**79**		**50**	**1.7%**	**1.00 [0.91, 1.11]**
Total events	72		46			
Heterogeneity: Tau² = 0.00; Chi² = 2.16, df = 2 (P = 0.34); I² = 7%						
Test for overall effect: Z = 0.06 (P = 0.95)						
**Total (95% CI)**		**535**		**469**	**100.0%**	**1.00 [0.99, 1.01]**
Total events	486		432			
Heterogeneity: Tau² = 0.00; Chi² = 12.80, df = 13 (P = 0.46); I² = 0%						
Test for overall effect: Z = 0.01 (P = 0.99)						
Test for subgroup differences: Chi² = 0.06, df = 2 (P = 0.97), I² = 0%						

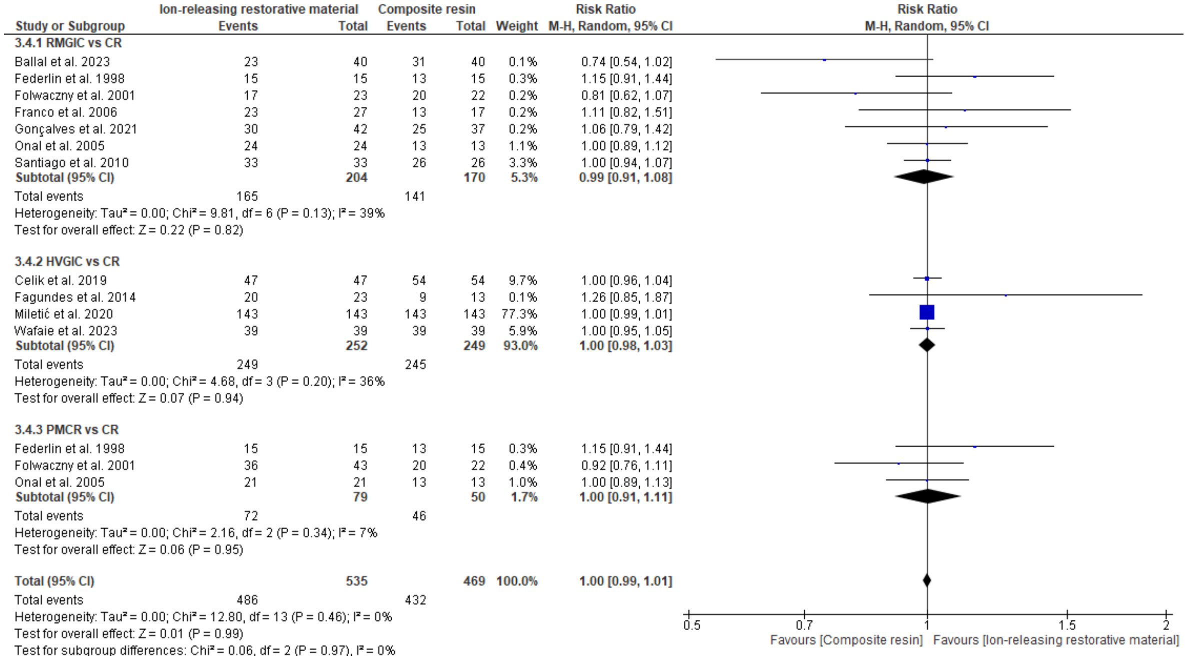

Figure S17. Adequate color or translucency between IRR and CR in dental restorations by restorative material

Study or Subgroup	Ion-releasing restorative material Events	Total	Composite resin Events	Total	Weight	Risk Ratio M-H, Random, 95% CI
**3.5.1 RMGIC vs CR**						
Brackett et al. 2003	26	26	22	22	4.2%	1.00 [0.92, 1.08]
de Oliveira et al. 2012	38	38	41	41	10.3%	1.00 [0.95, 1.05]
Federlin et al. 1998	15	15	15	15	1.8%	1.00 [0.88, 1.13]
Folwaczny et al. 2001	23	23	22	22	3.8%	1.00 [0.92, 1.09]
Gonçalves et al. 2021	27	42	29	37	0.4%	0.82 [0.62, 1.09]
Hussainy et al. 2018	32	33	32	34	2.6%	1.03 [0.93, 1.14]
Onal et al. 2005	24	24	13	13	2.1%	1.00 [0.89, 1.12]
Ozgünaltay et al. 2002	44	44	40	40	11.4%	1.00 [0.96, 1.05]
Perdigão et al. 2012	26	26	22	27	0.8%	1.22 [1.01, 1.48]
Popescu et al. 2016	50	57	44	53	1.2%	1.06 [0.90, 1.23]
van Dijken et al. 2019	77	82	82	82	7.2%	0.94 [0.88, 1.00]
**Subtotal (95% CI)**		**410**		**386**	**45.8%**	**1.00 [0.97, 1.02]**
Total events	382		362			
Heterogeneity: Tau² = 0.00; Chi² = 10.96, df = 10 (P = 0.36); I² = 9%						
Test for overall effect: Z = 0.36 (P = 0.72)						
**3.5.2 HVGIC vs CR**						
Balkaya et al. 2020	15	21	32	32	0.4%	0.72 [0.54, 0.94]
Celik et al. 2019	47	47	54	54	15.2%	1.00 [0.96, 1.04]
Schwendicke et al. 2021	41	41	49	49	12.6%	1.00 [0.96, 1.04]
Wafaie et al. 2023	37	37	39	39	9.7%	1.00 [0.95, 1.05]
**Subtotal (95% CI)**		**146**		**174**	**37.8%**	**0.98 [0.91, 1.05]**
Total events	140		174			
Heterogeneity: Tau² = 0.00; Chi² = 17.91, df = 3 (P = 0.0005); I² = 83%						
Test for overall effect: Z = 0.52 (P = 0.60)						
**3.5.3 PMCR vs CR**						
Federlin et al. 1998	15	15	15	15	1.8%	1.00 [0.88, 1.13]
Folwaczny et al. 2001	43	43	22	22	5.6%	1.00 [0.93, 1.07]
Hussainy et al. 2018	33	34	32	34	2.6%	1.03 [0.93, 1.14]
Onal et al. 2005	21	21	13	13	2.0%	1.00 [0.89, 1.13]
Türkün et al. 2008	50	50	47	50	4.3%	1.06 [0.98, 1.15]
**Subtotal (95% CI)**		**163**		**134**	**16.3%**	**1.02 [0.98, 1.06]**
Total events	162		129			
Heterogeneity: Tau² = 0.00; Chi² = 1.72, df = 4 (P = 0.79); I² = 0%						
Test for overall effect: Z = 1.01 (P = 0.31)						
**Total (95% CI)**		**719**		**694**	**100.0%**	**1.00 [0.98, 1.02]**
Total events	684		665			
Heterogeneity: Tau² = 0.00; Chi² = 20.48, df = 19 (P = 0.37); I² = 7%						
Test for overall effect: Z = 0.01 (P = 0.99)						
Test for subgroup differences: Chi² = 1.42, df = 2 (P = 0.49), I² = 0%						

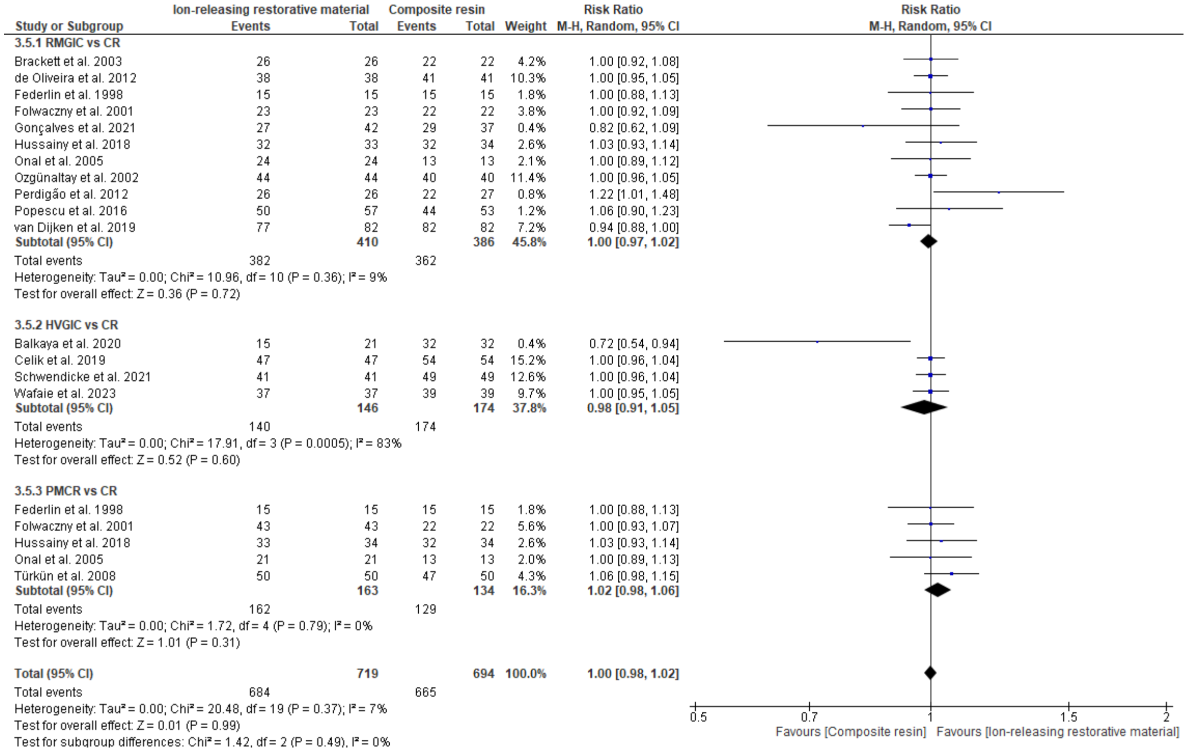

Figure S18. Proper surface texture or luster between IRR and CR in dental restorations by restorative material

Study or Subgroup	Ion-releasing restorative material Events	Total	Composite resin Events	Total	Weight	Risk Ratio M-H, Random, 95% CI
**3.6.1 RMGIC vs CR**						
Brackett et al. 2003	26	26	22	22	5.5%	1.00 [0.92, 1.08]
Federlin et al. 1998	15	15	15	15	2.8%	1.00 [0.88, 1.13]
Folwaczny et al. 2001	23	23	22	22	5.1%	1.00 [0.92, 1.09]
Gonçalves et al. 2021	34	42	35	37	1.7%	0.86 [0.73, 1.01]
Hussainy et al. 2018	32	33	32	34	3.8%	1.03 [0.93, 1.14]
Onal et al. 2005	24	24	13	13	3.2%	1.00 [0.89, 1.12]
Perdigão et al. 2012	11	26	25	27	0.2%	0.46 [0.29, 0.72]
Saghir et al. 2023	25	30	18	30	0.4%	1.39 [1.00, 1.94]
van Dijken et al. 2019	82	82	82	82	15.0%	1.00 [0.98, 1.02]
**Subtotal (95% CI)**		**301**		**282**	**37.8%**	**0.99 [0.92, 1.06]**
Total events	272		264			
Heterogeneity: Tau² = 0.01; Chi² = 31.92, df = 8 (P < 0.0001); I² = 75%						
Test for overall effect: Z = 0.40 (P = 0.69)						
**3.6.2 HVGIC vs CR**						
Balkaya et al. 2020	19	21	32	32	1.9%	0.90 [0.77, 1.05]
Celik et al. 2019	47	47	54	54	11.8%	1.00 [0.96, 1.04]
Schwendicke et al. 2021	41	41	49	49	10.8%	1.00 [0.96, 1.04]
Wafaie et al. 2023	37	37	39	39	9.5%	1.00 [0.95, 1.05]
**Subtotal (95% CI)**		**146**		**174**	**34.0%**	**1.00 [0.97, 1.02]**
Total events	144		174			
Heterogeneity: Tau² = 0.00; Chi² = 2.90, df = 3 (P = 0.41); I² = 0%						
Test for overall effect: Z = 0.21 (P = 0.83)						
**3.6.3 PMCR vs CR**						
Federlin et al. 1998	15	15	15	15	2.8%	1.00 [0.88, 1.13]
Folwaczny et al. 2001	43	43	22	22	6.8%	1.00 [0.93, 1.07]
Hussainy et al. 2018	33	34	32	34	3.8%	1.03 [0.93, 1.14]
Onal et al. 2005	21	21	13	13	3.0%	1.00 [0.89, 1.13]
Türkün et al. 2008	50	50	50	50	11.8%	1.00 [0.96, 1.04]
**Subtotal (95% CI)**		**163**		**134**	**28.2%**	**1.00 [0.97, 1.03]**
Total events	162		132			
Heterogeneity: Tau² = 0.00; Chi² = 0.40, df = 4 (P = 0.98); I² = 0%						
Test for overall effect: Z = 0.17 (P = 0.86)						
**Total (95% CI)**		**610**		**590**	**100.0%**	**1.00 [0.98, 1.02]**
Total events	578		570			
Heterogeneity: Tau² = 0.00; Chi² = 28.92, df = 17 (P = 0.04); I² = 41%						
Test for overall effect: Z = 0.23 (P = 0.82)						
Test for subgroup differences: Chi² = 0.21, df = 2 (P = 0.90), I² = 0%						

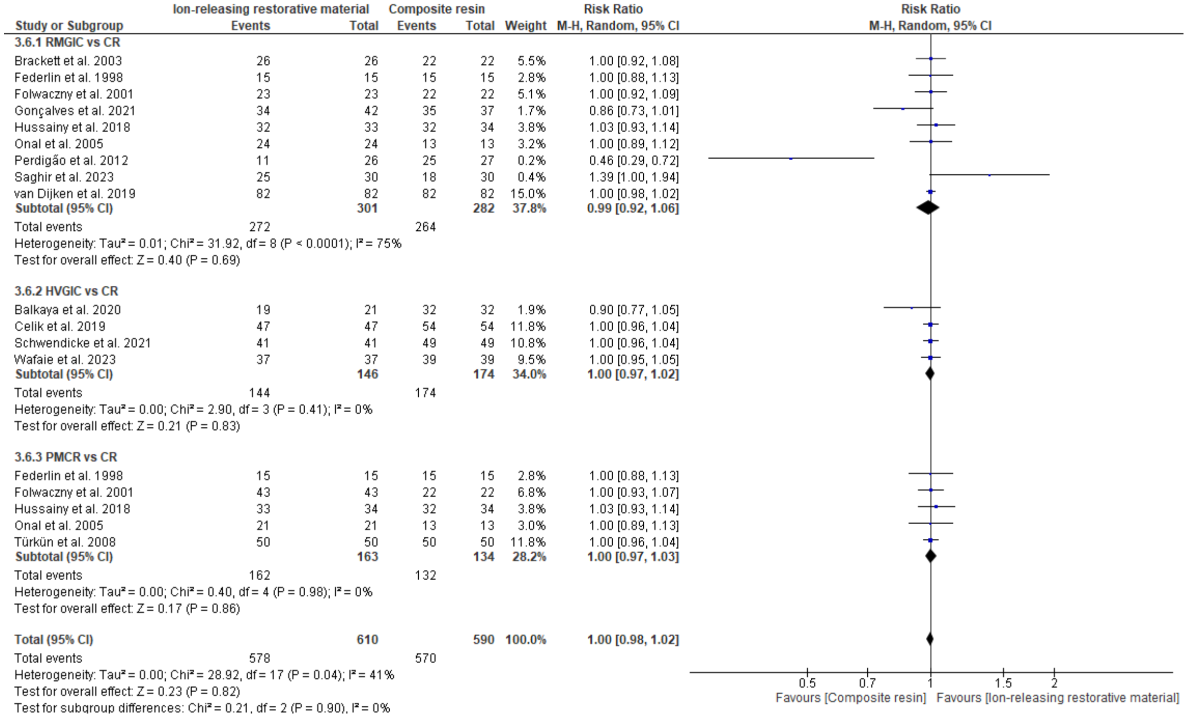

Figure S19. Proper surface staining between IRR and CR in dental restorations by restorative material

Study or Subgroup	Ion-releasing restorative material Events	Ion-releasing restorative material Total	Composite resin Events	Composite resin Total	Weight	Risk Ratio M-H, Random, 95% CI
**3.7.1 HVGIC vs CR**						
Celik et al. 2019	47	47	54	54	9.7%	1.00 [0.96, 1.04]
Miletić et al. 2020	143	143	143	143	77.1%	1.00 [0.99, 1.01]
Schwendicke et al. 2021	41	41	49	49	7.6%	1.00 [0.96, 1.04]
Wafaie et al. 2023	37	37	39	39	5.6%	1.00 [0.95, 1.05]
**Subtotal (95% CI)**		**268**		**285**	**100.0%**	**1.00 [0.99, 1.01]**
Total events	268		285			
Heterogeneity: Tau² = 0.00; Chi² = 0.00, df = 3 (P = 1.00); I² = 0%						
Test for overall effect: Z = 0.00 (P = 1.00)						
**Total (95% CI)**		**268**		**285**	**100.0%**	**1.00 [0.99, 1.01]**
Total events	268		285			
Heterogeneity: Tau² = 0.00; Chi² = 0.00, df = 3 (P = 1.00); I² = 0%						
Test for overall effect: Z = 0.00 (P = 1.00)						
Test for subgroup differences: Not applicable						

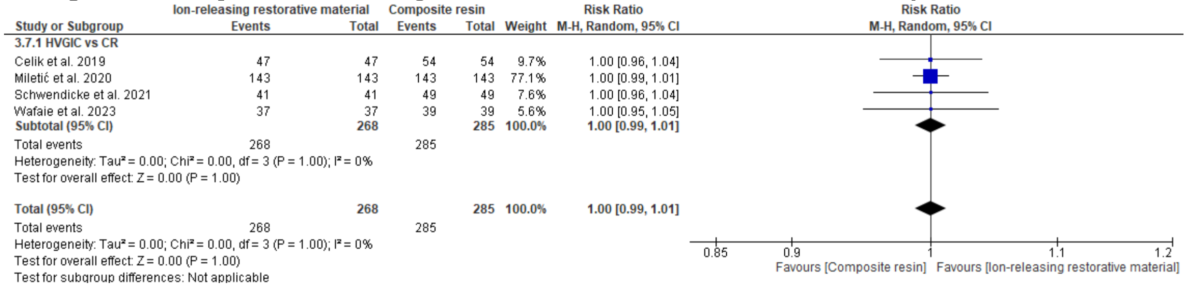

Figure S20. Retention between IRR and CR in dental restorations by restorative material

Study or Subgroup	Ion-releasing restorative material Events	Ion-releasing restorative material Total	Composite resin Events	Composite resin Total	Weight	Risk Ratio M-H, Random, 95% CI
**3.8.1 RMGIC vs CR**						
Adeleke et al. 2012	131	144	106	143	4.3%	1.23 [1.10, 1.37]
Ballal et al. 2023	24	40	33	40	1.4%	0.73 [0.54, 0.97]
Brackett et al. 2003	26	27	22	27	2.4%	1.18 [0.97, 1.44]
de Oliveira et al. 2012	38	40	41	41	5.0%	0.95 [0.87, 1.03]
Folwaczny et al. 2001	23	26	22	23	3.0%	0.92 [0.79, 1.09]
Franco et al. 2006	27	28	27	33	2.7%	1.18 [0.99, 1.41]
Gonçalves et al. 2021	42	43	37	42	4.0%	1.11 [0.98, 1.25]
Hussainy et al. 2018	32	33	32	34	4.5%	1.03 [0.93, 1.14]
Jassal et al. 2018	90	98	92	98	5.2%	0.98 [0.91, 1.06]
Koc Vural et al. 2021	47	51	43	51	3.4%	1.09 [0.95, 1.26]
Onal et al. 2005	24	24	13	18	1.3%	1.38 [1.03, 1.85]
Ozgünaltay et al. 2002	44	45	40	42	5.1%	1.03 [0.95, 1.11]
Perdigão et al. 2012	26	26	25	27	3.8%	1.08 [0.95, 1.22]
Popescu et al. 2016	54	57	53	57	4.7%	1.02 [0.93, 1.12]
Saghir et al. 2023	28	30	19	30	1.4%	1.47 [1.10, 1.97]
Santiago et al. 2010	33	33	26	33	2.6%	1.26 [1.05, 1.52]
van Dijken et al. 2019	62	82	80	82	3.8%	0.78 [0.68, 0.88]
**Subtotal (95% CI)**		**827**		**821**	**58.7%**	**1.05 [0.99, 1.12]**
Total events	751		711			
Heterogeneity: Tau² = 0.01; Chi² = 63.97, df = 16 (P < 0.00001); I² = 75%						
Test for overall effect: Z = 1.58 (P = 0.11)						
**3.8.2 HVGIC vs CR**						
Balkaya et al. 2020	15	21	32	32	1.5%	0.72 [0.54, 0.94]
Celik et al. 2019	47	49	54	54	5.5%	0.96 [0.90, 1.03]
Fagundes et al. 2014	23	26	13	25	0.8%	1.70 [1.14, 2.54]
Miletić et al. 2020	143	143	141	143	6.5%	1.01 [0.99, 1.04]
Schwendicke et al. 2021	39	41	49	49	5.1%	0.95 [0.88, 1.03]
Wafaie et al. 2023	37	39	39	39	4.9%	0.95 [0.87, 1.04]
**Subtotal (95% CI)**		**319**		**342**	**24.3%**	**0.97 [0.91, 1.04]**
Total events	304		328			
Heterogeneity: Tau² = 0.00; Chi² = 18.74, df = 5 (P = 0.002); I² = 73%						
Test for overall effect: Z = 0.86 (P = 0.39)						
**3.8.3 PMCR vs CR**						
Folwaczny et al. 2001	43	48	22	23	3.7%	0.94 [0.82, 1.07]
Hussainy et al. 2018	33	34	32	34	4.5%	1.03 [0.93, 1.14]
Onal et al. 2005	21	32	13	18	0.9%	0.91 [0.62, 1.33]
Pollington et al. 2008	26	30	26	30	2.4%	1.00 [0.82, 1.22]
Türkün et al. 2008	48	50	50	50	5.5%	0.96 [0.90, 1.03]
**Subtotal (95% CI)**		**194**		**155**	**17.0%**	**0.97 [0.93, 1.02]**
Total events	171		143			
Heterogeneity: Tau² = 0.00; Chi² = 1.92, df = 4 (P = 0.75); I² = 0%						
Test for overall effect: Z = 1.01 (P = 0.31)						
**Total (95% CI)**		**1340**		**1318**	**100.0%**	**1.02 [0.98, 1.05]**
Total events	1226		1182			
Heterogeneity: Tau² = 0.01; Chi² = 91.23, df = 27 (P < 0.00001); I² = 70%						
Test for overall effect: Z = 0.78 (P = 0.43)						
Test for subgroup differences: Chi² = 4.18, df = 2 (P = 0.12), I² = 52.1%						

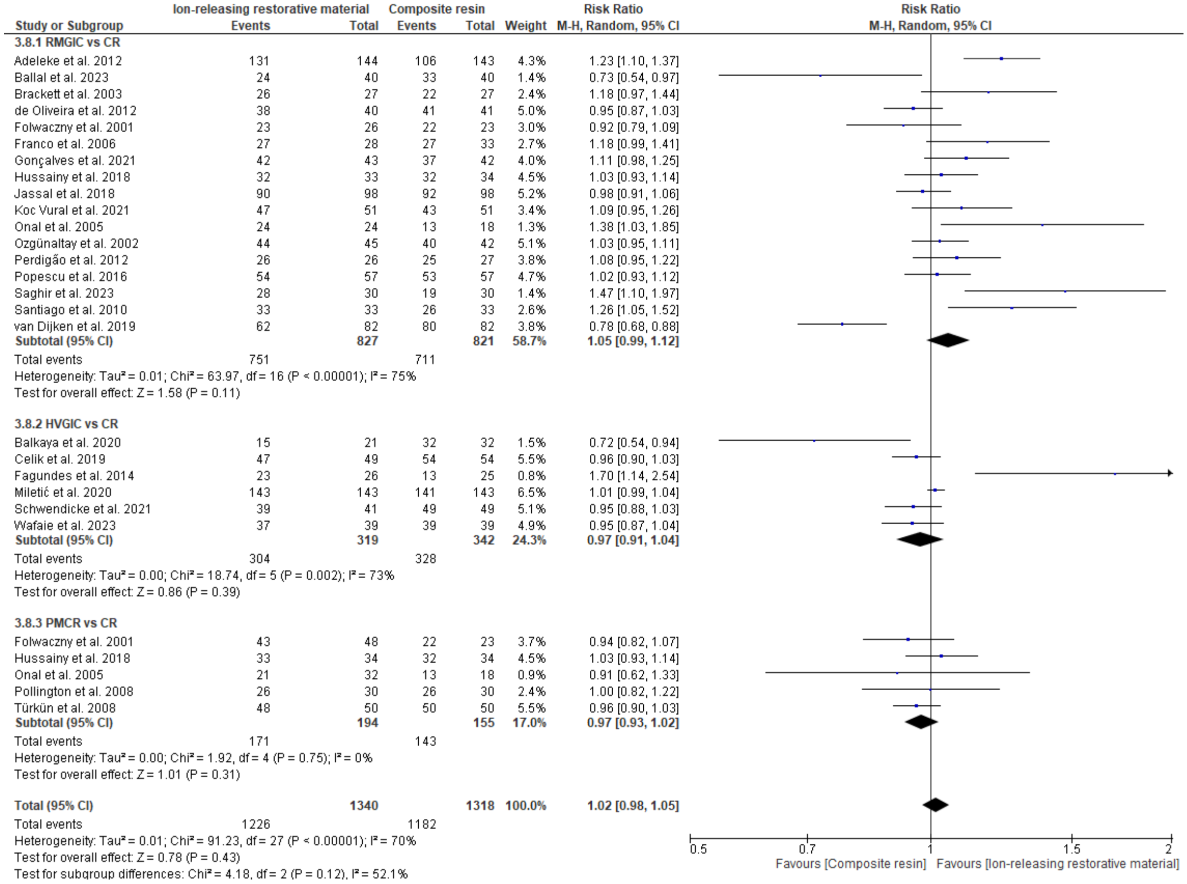

Figure S21. Absence of wear between IRR and CR in dental restorations by restorative material

Study or Subgroup	Ion-releasing restorative material Events	Total	Composite resin Events	Total	Weight	Risk Ratio M-H, Random, 95% CI
**3.9.1 RMGIC vs CR**						
Adeleke et al. 2012	131	131	106	106	33.4%	1.00 [0.98, 1.02]
Gonçalves et al. 2021	39	42	36	37	0.9%	0.95 [0.86, 1.05]
Perdigão et al. 2012	26	26	25	27	0.6%	1.08 [0.95, 1.22]
**Subtotal (95% CI)**		**199**		**170**	**34.9%**	**1.00 [0.97, 1.03]**
Total events	196		167			
Heterogeneity: Tau² = 0.00; Chi² = 2.20, df = 2 (P = 0.33); I² = 9%						
Test for overall effect: Z = 0.00 (P = 1.00)						
**3.9.2 HVGIC vs CR**						
Celik et al. 2019	47	47	54	54	6.3%	1.00 [0.96, 1.04]
Miletić et al. 2020	143	143	143	143	50.2%	1.00 [0.99, 1.01]
Schwendicke et al. 2021	41	41	49	49	5.0%	1.00 [0.96, 1.04]
Wafaie et al. 2023	37	37	39	39	3.6%	1.00 [0.95, 1.05]
**Subtotal (95% CI)**		**268**		**285**	**65.1%**	**1.00 [0.99, 1.01]**
Total events	268		285			
Heterogeneity: Tau² = 0.00; Chi² = 0.00, df = 3 (P = 1.00); I² = 0%						
Test for overall effect: Z = 0.00 (P = 1.00)						
**Total (95% CI)**		**467**		**455**	**100.0%**	**1.00 [0.99, 1.01]**
Total events	464		452			
Heterogeneity: Tau² = 0.00; Chi² = 2.19, df = 6 (P = 0.90); I² = 0%						
Test for overall effect: Z = 0.00 (P = 1.00)						
Test for subgroup differences: Chi² = 0.00, df = 1 (P = 1.00), I² = 0%						

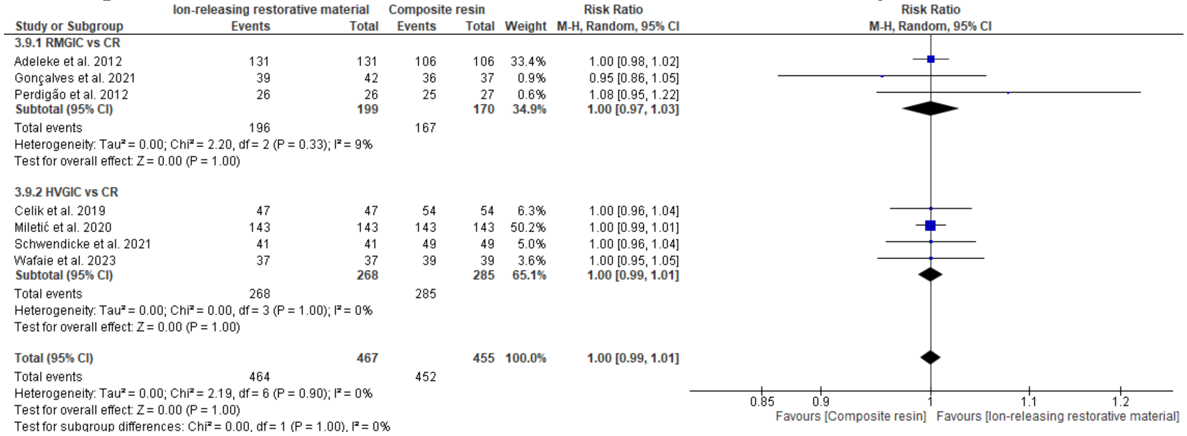

Figure S22. Proper anatomic form between IRR and CR in dental restorations by restorative material

Study or Subgroup	Ion-releasing restorative material Events	Total	Composite resin Events	Total	Weight	Risk Ratio M-H, Random, 95% CI
**3.10.1 RMGIC vs CR**						
Ballal et al. 2023	22	40	32	40	0.7%	0.69 [0.50, 0.95]
Brackett et al. 2003	26	26	22	22	5.3%	1.00 [0.92, 1.08]
de Oliveira et al. 2012	38	38	41	41	7.2%	1.00 [0.95, 1.05]
Federlin et al. 1998	15	15	15	15	3.3%	1.00 [0.88, 1.13]
Folwaczny et al. 2001	14	23	22	22	0.7%	0.62 [0.44, 0.86]
Franco et al. 2006	23	27	15	17	1.2%	0.97 [0.76, 1.22]
Gonçalves et al. 2021	40	42	36	37	4.9%	0.98 [0.90, 1.07]
Onal et al. 2005	24	24	13	13	3.6%	1.00 [0.89, 1.12]
Ozgünaltay et al. 2002	44	44	40	40	7.4%	1.00 [0.96, 1.05]
Popescu et al. 2016	57	57	53	53	8.1%	1.00 [0.97, 1.04]
Santiago et al. 2010	33	33	25	26	4.2%	1.04 [0.94, 1.15]
van Dijken et al. 2019	74	82	81	82	5.5%	0.91 [0.85, 0.98]
**Subtotal (95% CI)**		**451**		**408**	**52.0%**	**0.97 [0.93, 1.02]**
Total events	410		395			
Heterogeneity: Tau² = 0.00; Chi² = 47.54, df = 11 (P < 0.00001); I² = 77%						
Test for overall effect: Z = 1.03 (P = 0.30)						
**3.10.2 HVGIC vs CR**						
Balkaya et al. 2020	20	21	32	32	3.4%	0.95 [0.84, 1.07]
Celik et al. 2019	45	47	54	54	5.8%	0.96 [0.89, 1.03]
Fagundes et al. 2014	21	23	12	13	1.6%	0.99 [0.81, 1.21]
Miletić et al. 2020	143	143	143	143	9.1%	1.00 [0.99, 1.01]
Schwendicke et al. 2021	41	41	49	49	7.6%	1.00 [0.96, 1.04]
Wafaie et al. 2023	37	37	39	39	7.1%	1.00 [0.95, 1.05]
**Subtotal (95% CI)**		**312**		**330**	**34.5%**	**1.00 [0.99, 1.01]**
Total events	307		329			
Heterogeneity: Tau² = 0.00; Chi² = 4.72, df = 5 (P = 0.45); I² = 0%						
Test for overall effect: Z = 0.31 (P = 0.76)						
**3.10.3 PMCR vs CR**						
Federlin et al. 1998	15	15	15	15	3.3%	1.00 [0.88, 1.13]
Folwaczny et al. 2001	40	43	22	22	4.0%	0.94 [0.85, 1.05]
Onal et al. 2005	21	21	13	13	3.4%	1.00 [0.89, 1.13]
Pollington et al. 2008	27	30	29	30	2.9%	0.93 [0.81, 1.07]
**Subtotal (95% CI)**		**109**		**80**	**13.5%**	**0.97 [0.91, 1.03]**
Total events	103		79			
Heterogeneity: Tau² = 0.00; Chi² = 1.22, df = 3 (P = 0.75); I² = 0%						
Test for overall effect: Z = 1.09 (P = 0.28)						
**Total (95% CI)**		**872**		**818**	**100.0%**	**0.98 [0.95, 1.01]**
Total events	820		803			
Heterogeneity: Tau² = 0.00; Chi² = 71.62, df = 21 (P < 0.00001); I² = 71%						
Test for overall effect: Z = 1.38 (P = 0.17)						
Test for subgroup differences: Chi² = 1.79, df = 2 (P = 0.41), I² = 0%						

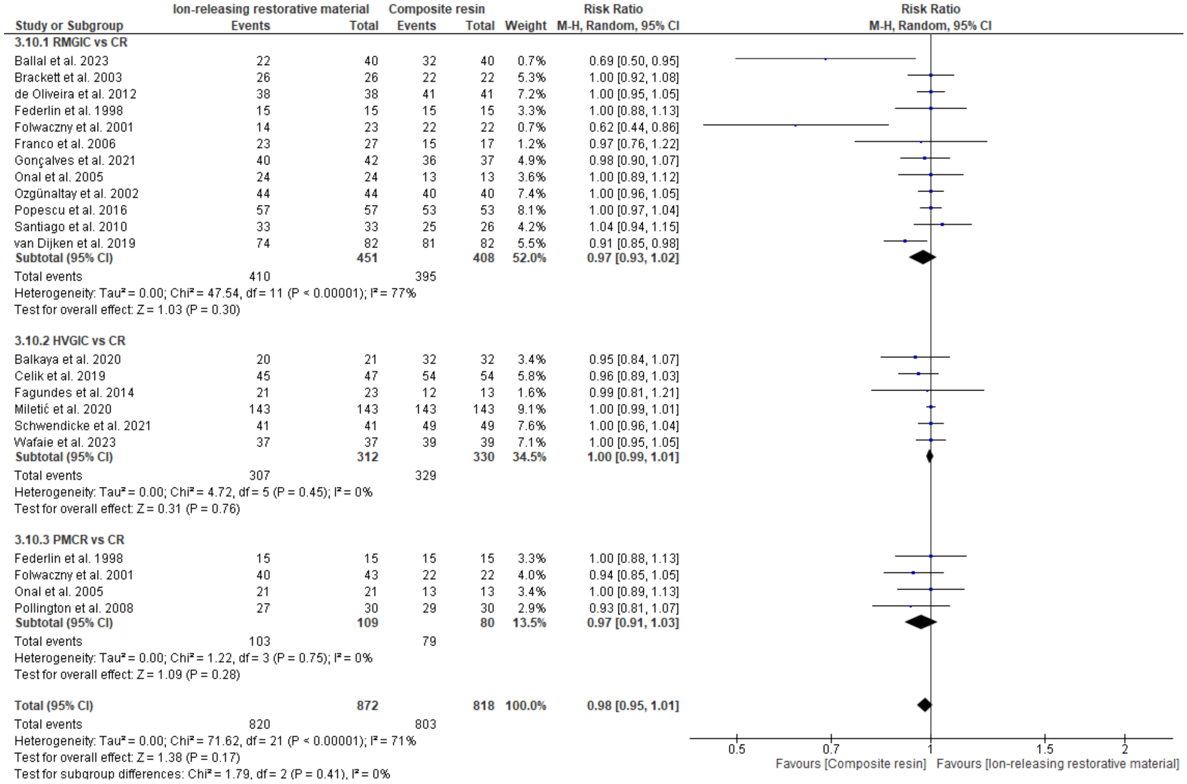

Figure S23. Absence of sensibility between IRR and CR in dental restorations by restorative material

Study or Subgroup	Ion-releasing restorative material Events	Total	Composite resin Events	Total	Weight	Risk Ratio M-H, Random, 95% CI
**3.11.1 RMGIC vs CR**						
Adeleke et al. 2012	131	131	106	106	24.9%	1.00 [0.98, 1.02]
Hussainy et al. 2018	32	33	32	34	0.6%	1.03 [0.93, 1.14]
Jassal et al. 2018	90	90	92	92	15.2%	1.00 [0.98, 1.02]
Koc Vural et al. 2021	47	47	43	43	3.8%	1.00 [0.96, 1.04]
Perdigão et al. 2012	25	26	24	27	0.3%	1.08 [0.93, 1.26]
**Subtotal (95% CI)**		**327**		**302**	**44.8%**	**1.00 [0.99, 1.01]**
Total events	325		297			
Heterogeneity: Tau² = 0.00; Chi² = 3.01, df = 4 (P = 0.56); I² = 0%						
Test for overall effect: Z = 0.15 (P = 0.88)						
**3.11.2 HVGIC vs CR**						
Balkaya et al. 2020	21	21	32	32	1.2%	1.00 [0.93, 1.08]
Celik et al. 2019	47	47	54	54	4.7%	1.00 [0.96, 1.04]
Miletić et al. 2020	143	143	143	143	37.4%	1.00 [0.99, 1.01]
Schwendicke et al. 2021	41	41	49	49	3.7%	1.00 [0.96, 1.04]
Wafaie et al. 2023	39	39	39	39	2.9%	1.00 [0.95, 1.05]
**Subtotal (95% CI)**		**291**		**317**	**49.8%**	**1.00 [0.99, 1.01]**
Total events	291		317			
Heterogeneity: Tau² = 0.00; Chi² = 0.00, df = 4 (P = 1.00); I² = 0%						
Test for overall effect: Z = 0.00 (P = 1.00)						
**3.11.3 PMCR vs CR**						
Hussainy et al. 2018	33	34	32	34	0.7%	1.03 [0.93, 1.14]
Türkün et al. 2008	50	50	50	50	4.7%	1.00 [0.96, 1.04]
**Subtotal (95% CI)**		**84**		**84**	**5.3%**	**1.00 [0.97, 1.04]**
Total events	83		82			
Heterogeneity: Tau² = 0.00; Chi² = 0.50, df = 1 (P = 0.48); I² = 0%						
Test for overall effect: Z = 0.21 (P = 0.84)						
**Total (95% CI)**		**702**		**703**	**100.0%**	**1.00 [0.99, 1.01]**
Total events	699		696			
Heterogeneity: Tau² = 0.00; Chi² = 2.98, df = 11 (P = 0.99); I² = 0%						
Test for overall effect: Z = 0.15 (P = 0.88)						
Test for subgroup differences: Chi² = 0.04, df = 2 (P = 0.98), I² = 0%						

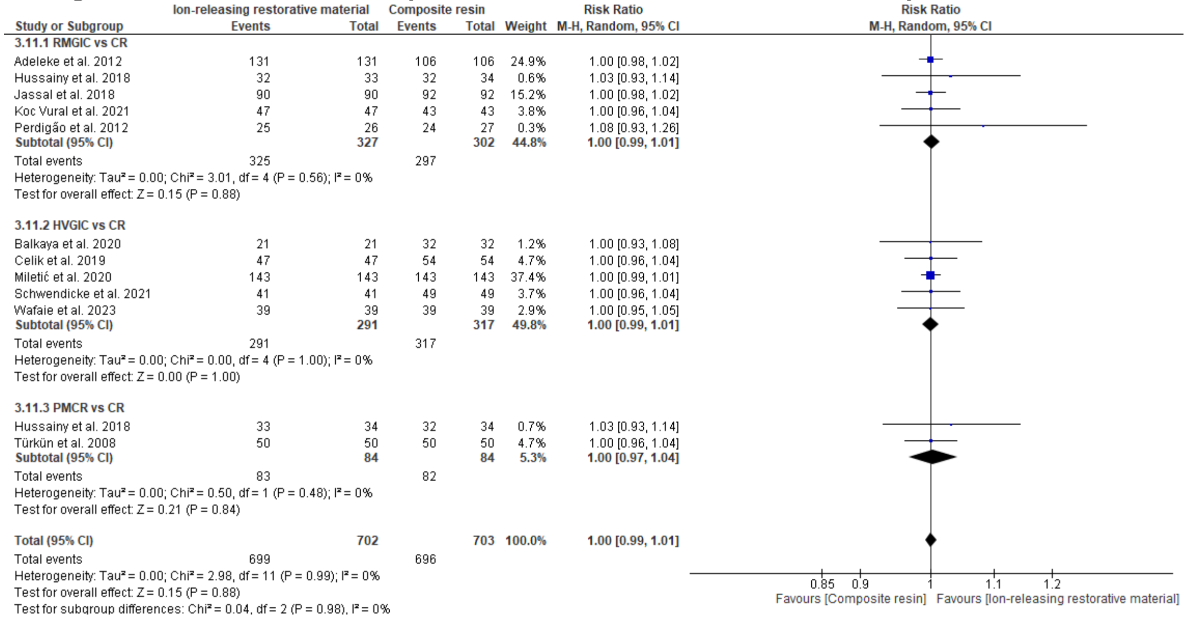

Figure S24. Adequate periodontal tissue between IRR and CR in dental restorations by restorative material

Study or Subgroup	Ion-releasing restorative material Events	Total	Composite resin Events	Total	Weight	Risk Ratio M-H, Random, 95% CI
**3.12.1 HVGIC vs CR**						
Celik et al. 2019	47	47	52	54	5.0%	1.04 [0.97, 1.11]
Miletić et al. 2020	144	144	144	144	86.6%	1.00 [0.99, 1.01]
Wafaie et al. 2023	39	39	39	39	8.4%	1.00 [0.95, 1.05]
**Subtotal (95% CI)**		**230**		**237**	**100.0%**	**1.00 [0.99, 1.02]**
Total events	230		235			
Heterogeneity: Tau² = 0.00; Chi² = 2.07, df = 2 (P = 0.36); I² = 3%						
Test for overall effect: Z = 0.24 (P = 0.81)						
**Total (95% CI)**		**230**		**237**	**100.0%**	**1.00 [0.99, 1.02]**
Total events	230		235			
Heterogeneity: Tau² = 0.00; Chi² = 2.07, df = 2 (P = 0.36); I² = 3%						
Test for overall effect: Z = 0.24 (P = 0.81)						
Test for subgroup differences: Not applicable						

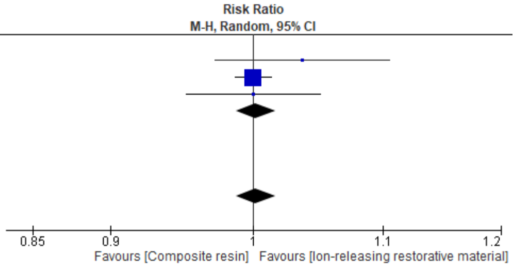

Figure S25. Absence of secondary caries or erosion or abfraction between IRR and CR in dental restorations by cavity types

	Ion-releasing restorative material		Composite resin			Risk Ratio
**Study or Subgroup**	**Events**	**Total**	**Events**	**Total**	**Weight**	**M-H, Random, 95% CI**
**2.1.1 Class I and II**						
Albelasy et al. 2024	27	27	27	27	4.2%	1.00 [0.93, 1.07]
Toz-Akalin et al. 2023	28	28	28	29	3.6%	1.03 [0.94, 1.14]
van Dijken et al. 2019	3	82	82	82	0.1%	0.04 [0.02, 0.12]
**Subtotal (95% CI)**		**137**		**138**	**7.9%**	**0.39 [0.09, 1.69]**
Total events	58		137			
Heterogeneity: Tau² = 1.58; Chi² = 873.23, df = 2 (P < 0.00001); I² = 100%						
Test for overall effect: Z = 1.25 (P = 0.21)						
**2.1.2 Class II**						
Balkaya et al. 2020	21	21	32	32	4.0%	1.00 [0.93, 1.08]
Miletić et al. 2020	143	143	143	143	5.1%	1.00 [0.99, 1.01]
Wafaie et al. 2023	37	37	39	39	4.6%	1.00 [0.95, 1.05]
**Subtotal (95% CI)**		**201**		**214**	**13.7%**	**1.00 [0.99, 1.01]**
Total events	201		214			
Heterogeneity: Tau² = 0.00; Chi² = 0.00, df = 2 (P = 1.00); I² = 0%						
Test for overall effect: Z = 0.00 (P = 1.00)						
**2.1.3 Class V**						
Adeleke et al. 2012	117	117	72	72	5.0%	1.00 [0.98, 1.02]
Ballal et al. 2023	40	40	40	40	4.6%	1.00 [0.95, 1.05]
Brackett et al. 2003	26	26	22	22	4.0%	1.00 [0.92, 1.08]
Celik et al. 2019	47	47	54	54	4.8%	1.00 [0.96, 1.04]
de Oliveira et al. 2012	38	38	41	41	4.6%	1.00 [0.95, 1.05]
Fagundes et al. 2014	23	23	12	13	1.9%	1.10 [0.91, 1.33]
Federlin et al. 1998	15	15	15	15	3.0%	1.00 [0.88, 1.13]
Franco et al. 2006	27	27	15	17	1.9%	1.14 [0.94, 1.38]
Gonçalves et al. 2021	42	42	37	37	4.6%	1.00 [0.95, 1.05]
Jassal et al. 2018	90	90	92	92	5.0%	1.00 [0.98, 1.02]
Koc Vural et al. 2021	47	47	43	43	4.7%	1.00 [0.96, 1.04]
Matis et al. 1996	12	12	3	3	0.6%	1.00 [0.68, 1.47]
Onal et al. 2005	24	24	13	13	3.2%	1.00 [0.89, 1.12]
Ozgünaltay et al. 2002	44	44	40	40	4.7%	1.00 [0.96, 1.05]
Perdigão et al. 2012	26	26	25	27	2.9%	1.08 [0.95, 1.22]
Pollington et al. 2008	30	30	30	30	4.3%	1.00 [0.94, 1.07]
Popescu et al. 2016	57	57	53	53	4.8%	1.00 [0.97, 1.04]
Santiago et al. 2010	33	33	26	26	4.3%	1.00 [0.94, 1.07]
Schwendicke et al. 2021	41	41	49	49	4.7%	1.00 [0.96, 1.04]
Türkün et al. 2008	50	50	50	50	4.8%	1.00 [0.96, 1.04]
**Subtotal (95% CI)**		**829**		**737**	**78.4%**	**1.00 [0.99, 1.01]**
Total events	829		732			
Heterogeneity: Tau² = 0.00; Chi² = 5.78, df = 19 (P = 1.00); I² = 0%						
Test for overall effect: Z = 0.21 (P = 0.83)						
**Total (95% CI)**		**1167**		**1089**	**100.0%**	**1.00 [0.97, 1.04]**
Total events	1088		1083			
Heterogeneity: Tau² = 0.01; Chi² = 322.94, df = 25 (P < 0.00001); I² = 92%						
Test for overall effect: Z = 0.26 (P = 0.80)						
Test for subgroup differences: Chi² = 1.59, df = 2 (P = 0.45), I² = 0%						

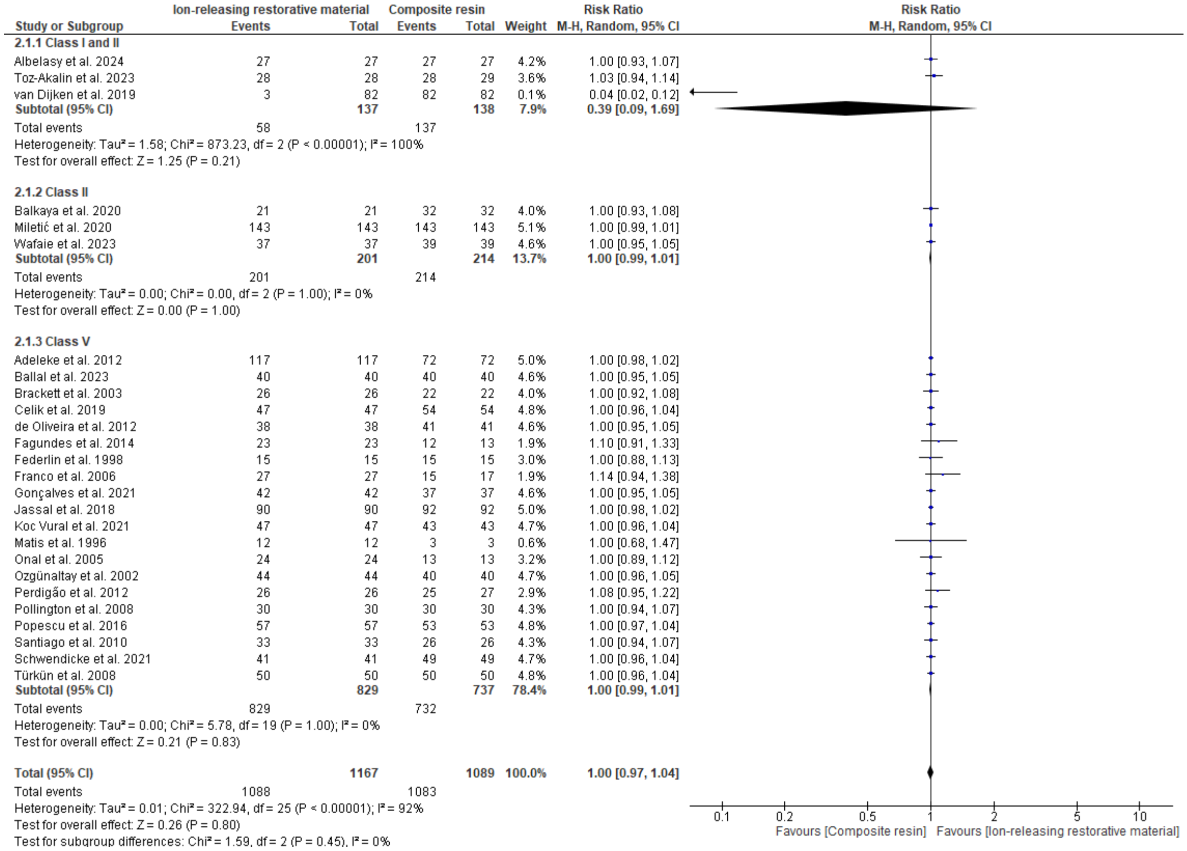

Figure S26. Absence of marginal discoloration between IRR and CR in dental restorations by cavity types

	Ion-releasing restorative material		Composite resin			Risk Ratio
Study or Subgroup	Events	Total	Events	Total	Weight	M-H, Random, 95% CI
**2.2.1 Class I and II**						
Albelasy et al. 2024	27	27	27	27	2.7%	1.00 [0.93, 1.07]
van Dijken et al. 2019	82	82	82	82	11.0%	1.00 [0.98, 1.02]
**Subtotal (95% CI)**		**109**		**109**	**13.7%**	**1.00 [0.98, 1.02]**
Total events	109		109			
Heterogeneity: Tau² = 0.00; Chi² = 0.00, df = 1 (P = 1.00); I² = 0%						
Test for overall effect: Z = 0.00 (P = 1.00)						
**2.2.2 Class II**						
Balkaya et al. 2020	20	21	32	32	1.0%	0.95 [0.84, 1.07]
Miletić et al. 2020	142	142	142	142	14.9%	1.00 [0.99, 1.01]
Wafaie et al. 2023	37	37	39	39	4.6%	1.00 [0.95, 1.05]
**Subtotal (95% CI)**		**200**		**213**	**20.5%**	**1.00 [0.99, 1.01]**
Total events	199		213			
Heterogeneity: Tau² = 0.00; Chi² = 1.63, df = 2 (P = 0.44); I² = 0%						
Test for overall effect: Z = 0.10 (P = 0.92)						
**2.2.3 Class V**						
Adeleke et al. 2012	130	131	105	106	10.9%	1.00 [0.98, 1.03]
Ballal et al. 2023	24	40	33	40	0.2%	0.73 [0.54, 0.97]
Brackett et al. 2003	26	26	22	22	2.2%	1.00 [0.92, 1.08]
Celik et al. 2019	47	47	54	54	6.7%	1.00 [0.96, 1.04]
de Oliveira et al. 2012	38	38	41	41	4.8%	1.00 [0.95, 1.05]
Fagundes et al. 2014	23	23	13	13	1.1%	1.00 [0.89, 1.12]
Federlin et al. 1998	15	15	15	15	1.0%	1.00 [0.88, 1.13]
Folwaczny et al. 2001	19	23	20	22	0.3%	0.91 [0.72, 1.14]
Franco et al. 2006	27	27	17	17	1.7%	1.00 [0.91, 1.10]
Gonçalves et al. 2021	29	42	25	37	0.2%	1.02 [0.76, 1.38]
Hussainy et al. 2018	32	33	32	34	1.4%	1.03 [0.93, 1.14]
Jassal et al. 2018	90	90	92	92	11.9%	1.00 [0.98, 1.02]
Koc Vural et al. 2021	31	47	40	43	0.3%	0.71 [0.57, 0.88]
Matis et al. 1996	12	12	3	3	0.1%	1.00 [0.68, 1.47]
Onal et al. 2005	24	24	13	13	1.1%	1.00 [0.89, 1.12]
Ozgünaltay et al. 2002	44	44	40	40	5.3%	1.00 [0.96, 1.05]
Perdigão et al. 2012	25	26	22	27	0.4%	1.18 [0.97, 1.43]
Pollington et al. 2008	28	30	28	30	0.8%	1.00 [0.87, 1.14]
Popescu et al. 2016	32	57	31	53	0.2%	0.96 [0.70, 1.33]
Santiago et al. 2010	33	33	26	26	3.0%	1.00 [0.94, 1.07]
Schwendicke et al. 2021	41	41	49	49	5.7%	1.00 [0.96, 1.04]
Türkün et al. 2008	50	50	50	50	6.7%	1.00 [0.96, 1.04]
**Subtotal (95% CI)**		**899**		**827**	**65.8%**	**1.00 [0.98, 1.02]**
Total events	820		771			
Heterogeneity: Tau² = 0.00; Chi² = 33.70, df = 21 (P = 0.04); I² = 38%						
Test for overall effect: Z = 0.19 (P = 0.85)						
**Total (95% CI)**		**1208**		**1149**	**100.0%**	**1.00 [0.99, 1.01]**
Total events	1128		1093			
Heterogeneity: Tau² = 0.00; Chi² = 37.12, df = 26 (P = 0.07); I² = 30%						
Test for overall effect: Z = 0.20 (P = 0.84)						
Test for subgroup differences: Chi² = 0.02, df = 2 (P = 0.99), I² = 0%						

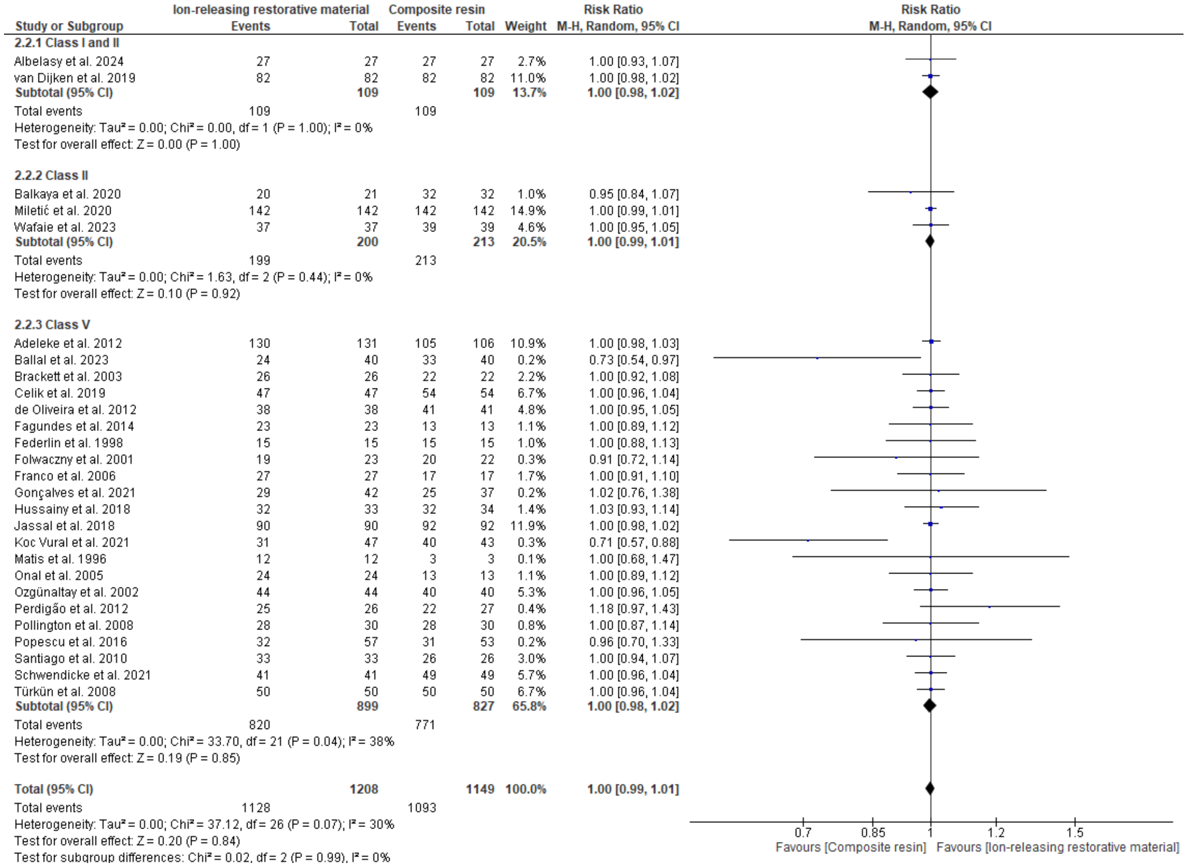

Figure S27. Adequate of marginal adaptation between IRR and CR in dental restorations by cavity types

Study or Subgroup	Ion-releasing restorative material Events	Ion-releasing restorative material Total	Composite resin Events	Composite resin Total	Weight	Risk Ratio M-H, Random, 95% CI
**2.3.1 Class I and II**						
Albelasy et al. 2024	27	27	27	27	1.5%	1.00 [0.93, 1.07]
Toz-Akalin et al. 2023	28	28	29	29	1.6%	1.00 [0.94, 1.07]
van Dijken et al. 2019	74	82	81	82	1.3%	0.91 [0.85, 0.98]
**Subtotal (95% CI)**		**137**		**138**	**4.4%**	**0.97 [0.91, 1.04]**
Total events	129		137			
Heterogeneity: Tau² = 0.00; Chi² = 5.43, df = 2 (P = 0.07); I² = 63%						
Test for overall effect: Z = 0.84 (P = 0.40)						
**2.3.2 Class II**						
Balkaya et al. 2020	20	21	32	32	0.5%	0.95 [0.84, 1.07]
Miletić et al. 2020	142	142	142	142	39.3%	1.00 [0.99, 1.01]
Wafaie et al. 2023	37	37	39	39	2.9%	1.00 [0.95, 1.05]
**Subtotal (95% CI)**		**200**		**213**	**42.7%**	**1.00 [0.99, 1.01]**
Total events	199		213			
Heterogeneity: Tau² = 0.00; Chi² = 1.63, df = 2 (P = 0.44); I² = 0%						
Test for overall effect: Z = 0.10 (P = 0.92)						
**2.3.3 Class V**						
Adeleke et al. 2012	131	131	105	106	11.9%	1.01 [0.99, 1.04]
Brackett et al. 2003	26	26	22	22	1.2%	1.00 [0.92, 1.08]
Celik et al. 2019	47	47	54	54	5.0%	1.00 [0.96, 1.04]
de Oliveira et al. 2012	38	38	41	41	3.1%	1.00 [0.95, 1.05]
Hussainy et al. 2018	32	33	32	34	0.7%	1.03 [0.93, 1.14]
Jassal et al. 2018	90	90	92	92	16.2%	1.00 [0.98, 1.02]
Koc Vural et al. 2021	47	47	43	43	4.0%	1.00 [0.96, 1.04]
Matis et al. 1996	10	12	2	3	0.0%	1.25 [0.54, 2.89]
Özgünaltay et al. 2002	44	44	40	40	3.5%	1.00 [0.96, 1.05]
Perdigão et al. 2012	26	26	23	27	0.3%	1.17 [0.99, 1.39]
Pollington et al. 2008	24	30	25	30	0.1%	0.96 [0.76, 1.22]
Popescu et al. 2016	32	57	30	53	0.1%	0.99 [0.71, 1.38]
Saghir et al. 2023	23	30	21	30	0.1%	1.10 [0.81, 1.49]
Schwendicke et al. 2021	40	41	49	49	1.8%	0.97 [0.91, 1.04]
Türkün et al. 2008	50	50	50	50	5.0%	1.00 [0.96, 1.04]
**Subtotal (95% CI)**		**702**		**674**	**52.9%**	**1.00 [0.99, 1.01]**
Total events	660		629			
Heterogeneity: Tau² = 0.00; Chi² = 6.88, df = 14 (P = 0.94); I² = 0%						
Test for overall effect: Z = 0.44 (P = 0.66)						
**Total (95% CI)**		**1039**		**1025**	**100.0%**	**1.00 [0.99, 1.01]**
Total events	988		979			
Heterogeneity: Tau² = 0.00; Chi² = 12.15, df = 20 (P = 0.91); I² = 0%						
Test for overall effect: Z = 0.01 (P = 0.99)						
Test for subgroup differences: Chi² = 0.88, df = 2 (P = 0.64), I² = 0%						

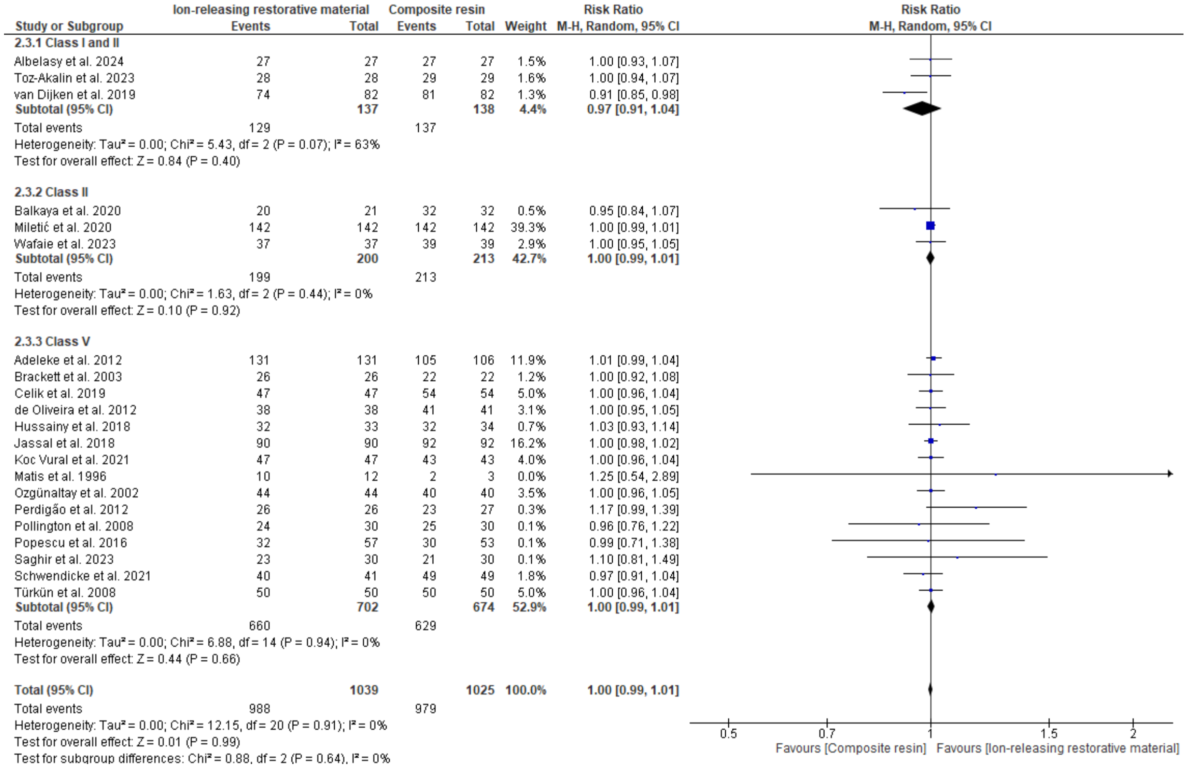

Figure S28. Adequate marginal or tooth integrity between IRR and CR in dental restorations by cavity types

Study or Subgroup	Ion-releasing restorative material Events	Ion-releasing restorative material Total	Composite resin Events	Composite resin Total	Weight	Risk Ratio M-H, Random, 95% CI
**2.4.1 Class I and II**						
Albelasy et al. 2024	26	27	27	27	1.4%	0.96 [0.87, 1.07]
Toz-Akalin et al. 2023	28	28	29	29	3.1%	1.00 [0.94, 1.07]
**Subtotal (95% CI)**		**55**		**56**	**4.5%**	**0.99 [0.94, 1.05]**
Total events	54		56			
Heterogeneity: Tau² = 0.00; Chi² = 0.41, df = 1 (P = 0.52); I² = 0%						
Test for overall effect: Z = 0.40 (P = 0.69)						
**2.4.2 Class II**						
Miletić et al. 2020	143	143	143	143	75.1%	1.00 [0.99, 1.01]
Wafaie et al. 2023	39	39	39	39	5.7%	1.00 [0.95, 1.05]
**Subtotal (95% CI)**		**182**		**182**	**80.9%**	**1.00 [0.99, 1.01]**
Total events	182		182			
Heterogeneity: Tau² = 0.00; Chi² = 0.00, df = 1 (P = 1.00); I² = 0%						
Test for overall effect: Z = 0.00 (P = 1.00)						
**2.4.3 Class V**						
Ballal et al. 2023	23	40	31	40	0.1%	0.74 [0.54, 1.02]
Celik et al. 2019	47	47	54	54	9.4%	1.00 [0.96, 1.04]
Fagundes et al. 2014	20	23	9	13	0.1%	1.26 [0.85, 1.87]
Federlin et al. 1998	15	15	13	15	0.3%	1.15 [0.91, 1.44]
Folwaczny et al. 2001	17	23	20	22	0.2%	0.81 [0.62, 1.07]
Franco et al. 2006	23	27	13	17	0.1%	1.11 [0.82, 1.51]
Gonçalves et al. 2021	30	42	25	37	0.2%	1.06 [0.79, 1.42]
Onal et al. 2005	24	24	13	13	1.1%	1.00 [0.89, 1.12]
Santiago et al. 2010	33	33	26	26	3.2%	1.00 [0.94, 1.07]
**Subtotal (95% CI)**		**274**		**237**	**14.7%**	**1.00 [0.95, 1.05]**
Total events	232		204			
Heterogeneity: Tau² = 0.00; Chi² = 10.02, df = 8 (P = 0.26); I² = 20%						
Test for overall effect: Z = 0.01 (P = 0.99)						
**Total (95% CI)**		**511**		**475**	**100.0%**	**1.00 [0.99, 1.01]**
Total events	468		442			
Heterogeneity: Tau² = 0.00; Chi² = 11.91, df = 12 (P = 0.45); I² = 0%						
Test for overall effect: Z = 0.08 (P = 0.94)						
Test for subgroup differences: Chi² = 0.15, df = 2 (P = 0.93), I² = 0%						

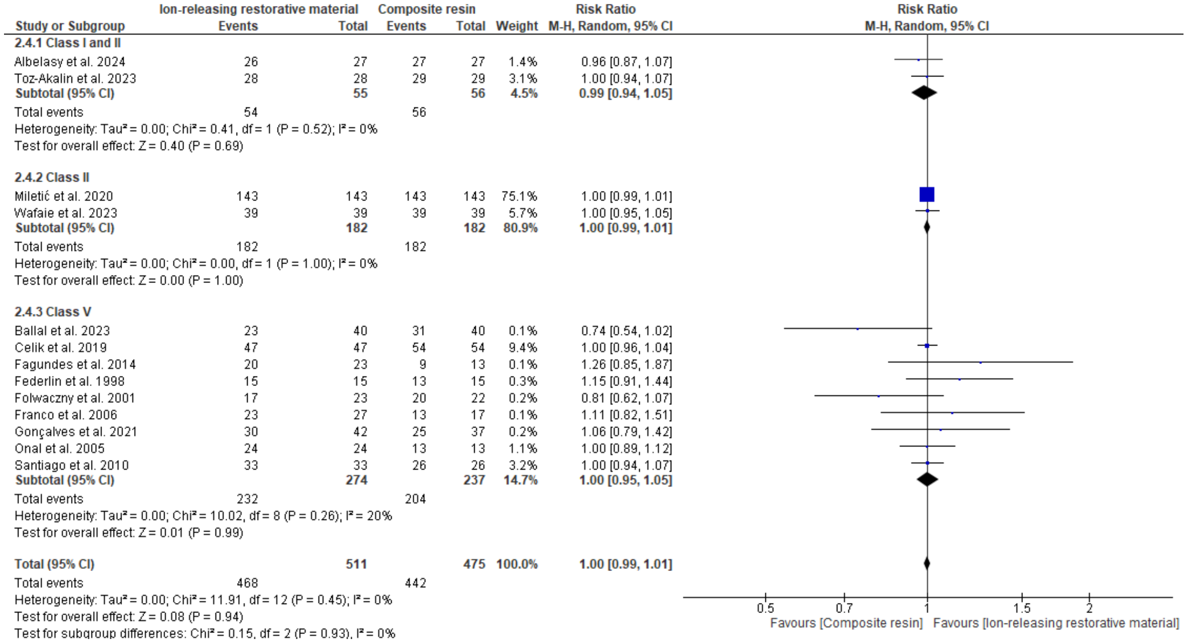

Figure S29. Adequate color or translucency between IRR and CR in dental restorations by cavity types

Study or Subgroup	Ion-releasing restorative material Events	Ion-releasing restorative material Total	Composite resin Events	Composite resin Total	Weight	Risk Ratio M-H, Random, 95% CI
**2.5.1 Class I and II**						
Toz-Akalin et al. 2023	28	28	29	29	5.9%	1.00 [0.94, 1.07]
van Dijken et al. 2019	77	82	82	82	7.2%	0.94 [0.88, 1.00]
**Subtotal (95% CI)**		**110**		**111**	**13.1%**	**0.97 [0.91, 1.03]**
Total events	105		111			
Heterogeneity: $Tau^2 = 0.00$; $Chi^2 = 2.10$, df = 1 (P = 0.15); $I^2 = 52\%$						
Test for overall effect: Z = 1.00 (P = 0.32)						
**2.5.2 Class II**						
Balkaya et al. 2020	15	21	32	32	0.4%	0.72 [0.54, 0.94]
Wafaie et al. 2023	37	37	39	39	9.3%	1.00 [0.95, 1.05]
**Subtotal (95% CI)**		**58**		**71**	**9.7%**	**0.85 [0.43, 1.70]**
Total events	52		71			
Heterogeneity: $Tau^2 = 0.24$; $Chi^2 = 24.59$, df = 1 (P < 0.00001); $I^2 = 96\%$						
Test for overall effect: Z = 0.46 (P = 0.65)						
**2.5.3 Class V**						
Brackett et al. 2003	26	26	22	22	4.4%	1.00 [0.92, 1.08]
Celik et al. 2019	47	47	54	54	13.6%	1.00 [0.96, 1.04]
de Oliveira et al. 2012	38	38	41	41	9.8%	1.00 [0.95, 1.05]
Federlin et al. 1998	15	15	15	15	1.9%	1.00 [0.88, 1.13]
Folwaczny et al. 2001	23	23	22	22	4.0%	1.00 [0.92, 1.09]
Gonçalves et al. 2021	27	42	29	37	0.4%	0.82 [0.62, 1.09]
Hussainy et al. 2018	32	33	32	34	2.7%	1.03 [0.93, 1.14]
Onal et al. 2005	24	24	13	13	2.2%	1.00 [0.89, 1.12]
Ozgünaltay et al. 2002	44	44	40	40	10.7%	1.00 [0.96, 1.05]
Perdigão et al. 2012	26	26	22	27	0.9%	1.22 [1.01, 1.48]
Popescu et al. 2016	50	57	44	53	1.3%	1.06 [0.90, 1.23]
Schwendicke et al. 2021	41	41	49	49	11.6%	1.00 [0.96, 1.04]
Türkün et al. 2008	50	50	50	50	13.6%	1.00 [0.96, 1.04]
**Subtotal (95% CI)**		**466**		**457**	**77.2%**	**1.00 [0.99, 1.02]**
Total events	443		433			
Heterogeneity: $Tau^2 = 0.00$; $Chi^2 = 7.09$, df = 12 (P = 0.85); $I^2 = 0\%$						
Test for overall effect: Z = 0.27 (P = 0.79)						
**Total (95% CI)**		**634**		**639**	**100.0%**	**1.00 [0.98, 1.01]**
Total events	600		615			
Heterogeneity: $Tau^2 = 0.00$; $Chi^2 = 19.24$, df = 16 (P = 0.26); $I^2 = 17\%$						
Test for overall effect: Z = 0.38 (P = 0.70)						
Test for subgroup differences: $Chi^2 = 1.28$, df = 2 (P = 0.53), $I^2 = 0\%$						

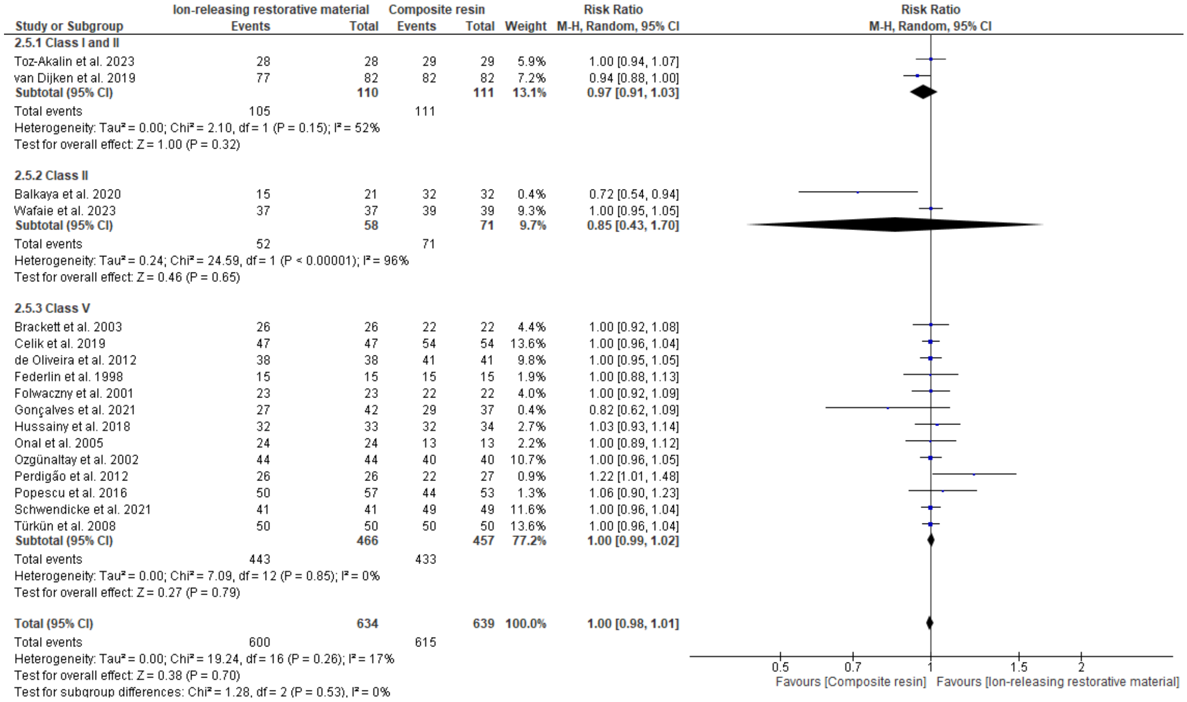

Figure S30. Proper surface texture or luster between IRR and CR in dental restorations by cavity types

Study or Subgroup	Ion-releasing restorative material Events	Ion-releasing restorative material Total	Composite resin Events	Composite resin Total	Weight	Risk Ratio M-H, Random, 95% CI
**2.6.1 Class I and II**						
Toz-Akalin et al. 2023	28	28	29	29	8.0%	1.00 [0.94, 1.07]
van Dijken et al. 2019	82	82	82	82	14.7%	1.00 [0.98, 1.02]
**Subtotal (95% CI)**		**110**		**111**	**22.7%**	**1.00 [0.98, 1.02]**
Total events	110		111			
Heterogeneity: $Tau^2 = 0.00$; $Chi^2 = 0.00$, df = 1 (P = 1.00); $I^2 = 0\%$						
Test for overall effect: Z = 0.00 (P = 1.00)						
**2.6.2 Class II**						
Balkaya et al. 2020	19	21	32	32	2.4%	0.90 [0.77, 1.05]
Wafaie et al. 2023	37	37	39	39	10.3%	1.00 [0.95, 1.05]
**Subtotal (95% CI)**		**58**		**71**	**12.8%**	**0.96 [0.83, 1.11]**
Total events	56		71			
Heterogeneity: $Tau^2 = 0.01$; $Chi^2 = 3.19$, df = 1 (P = 0.07); $I^2 = 69\%$						
Test for overall effect: Z = 0.55 (P = 0.59)						
**2.6.3 Class V**						
Brackett et al. 2003	26	26	22	22	6.6%	1.00 [0.92, 1.08]
Celik et al. 2019	47	47	54	54	12.3%	1.00 [0.96, 1.04]
Federlin et al. 1998	15	15	15	15	3.5%	1.00 [0.88, 1.13]
Folwaczny et al. 2001	23	23	22	22	6.2%	1.00 [0.92, 1.09]
Gonçalves et al. 2021	34	42	35	37	2.2%	0.86 [0.73, 1.01]
Hussainy et al. 2018	32	33	32	34	4.7%	1.03 [0.93, 1.14]
Matis et al. 1996	12	12	3	3	0.5%	1.00 [0.68, 1.47]
Onal et al. 2005	24	24	13	13	4.0%	1.00 [0.89, 1.12]
Perdigão et al. 2012	11	26	25	27	0.3%	0.46 [0.29, 0.72]
Saghir et al. 2023	25	30	18	30	0.6%	1.39 [1.00, 1.94]
Schwendicke et al. 2021	41	41	49	49	11.5%	1.00 [0.96, 1.04]
Türkün et al. 2008	50	50	50	50	12.3%	1.00 [0.96, 1.04]
**Subtotal (95% CI)**		**369**		**356**	**64.5%**	**0.99 [0.96, 1.03]**
Total events	340		338			
Heterogeneity: $Tau^2 = 0.00$; $Chi^2 = 27.20$, df = 11 (P = 0.004); $I^2 = 60\%$						
Test for overall effect: Z = 0.28 (P = 0.78)						
**Total (95% CI)**		**537**		**538**	**100.0%**	**0.99 [0.97, 1.02]**
Total events	506		520			
Heterogeneity: $Tau^2 = 0.00$; $Chi^2 = 31.43$, df = 15 (P = 0.008); $I^2 = 52\%$						
Test for overall effect: Z = 0.38 (P = 0.70)						
Test for subgroup differences: $Chi^2 = 0.33$, df = 2 (P = 0.85), $I^2 = 0\%$						

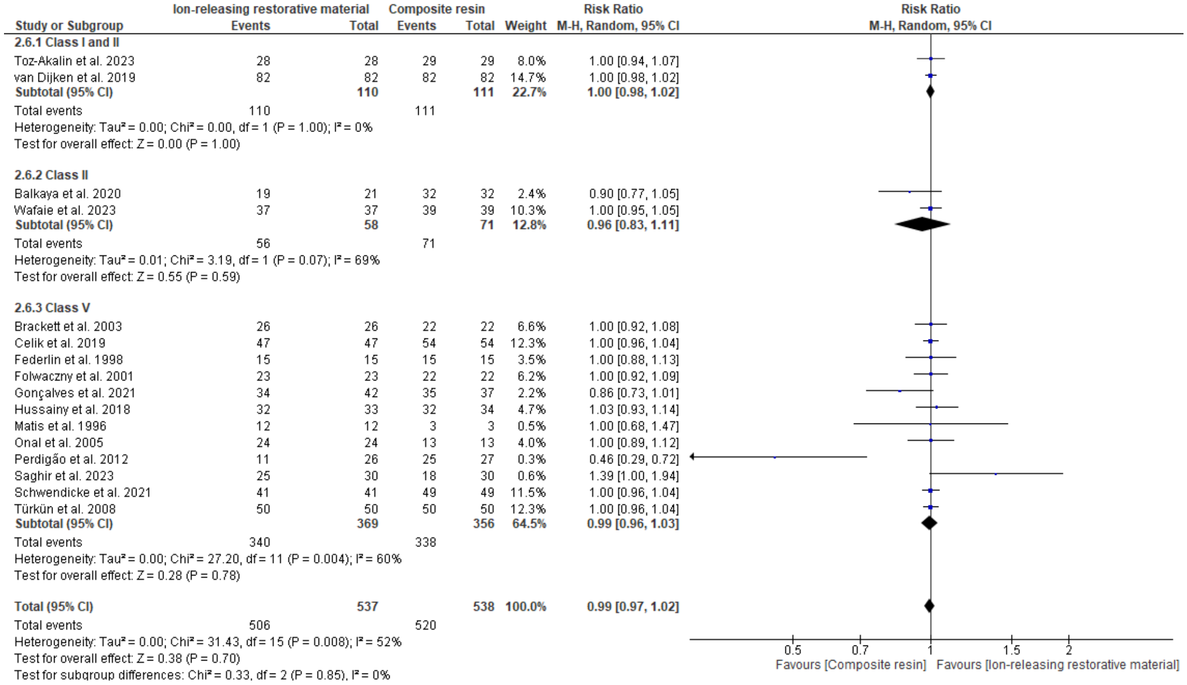

Figure S31. Proper surface staining between IRR and CR in dental restorations by cavity types

Study or Subgroup	Ion-releasing restorative material Events	Ion-releasing restorative material Total	Composite resin Events	Composite resin Total	Weight	Risk Ratio M-H, Random, 95% CI
**2.7.1 Class II**						
Miletić et al. 2020	143	143	143	143	76.0%	1.00 [0.99, 1.01]
Wafaie et al. 2023	37	37	39	39	5.5%	1.00 [0.95, 1.05]
**Subtotal (95% CI)**		**180**		**182**	**81.5%**	**1.00 [0.99, 1.01]**
Total events	180		182			
Heterogeneity: Tau² = 0.00; Chi² = 0.00, df = 1 (P = 1.00); I² = 0%						
Test for overall effect: Z = 0.00 (P = 1.00)						
**2.7.2 Class V**						
Celik et al. 2019	47	47	54	54	9.5%	1.00 [0.96, 1.04]
Gonçalves et al. 2021	40	42	35	37	1.3%	1.01 [0.91, 1.12]
Matis et al. 1996	11	12	3	3	0.1%	1.01 [0.66, 1.54]
Schwendicke et al. 2021	41	41	49	49	7.5%	1.00 [0.96, 1.04]
**Subtotal (95% CI)**		**142**		**143**	**18.5%**	**1.00 [0.97, 1.03]**
Total events	139		141			
Heterogeneity: Tau² = 0.00; Chi² = 0.03, df = 3 (P = 1.00); I² = 0%						
Test for overall effect: Z = 0.04 (P = 0.97)						
**Total (95% CI)**		**322**		**325**	**100.0%**	**1.00 [0.99, 1.01]**
Total events	319		323			
Heterogeneity: Tau² = 0.00; Chi² = 0.04, df = 5 (P = 1.00); I² = 0%						
Test for overall effect: Z = 0.02 (P = 0.99)						
Test for subgroup differences: Chi² = 0.00, df = 1 (P = 0.97), I² = 0%						

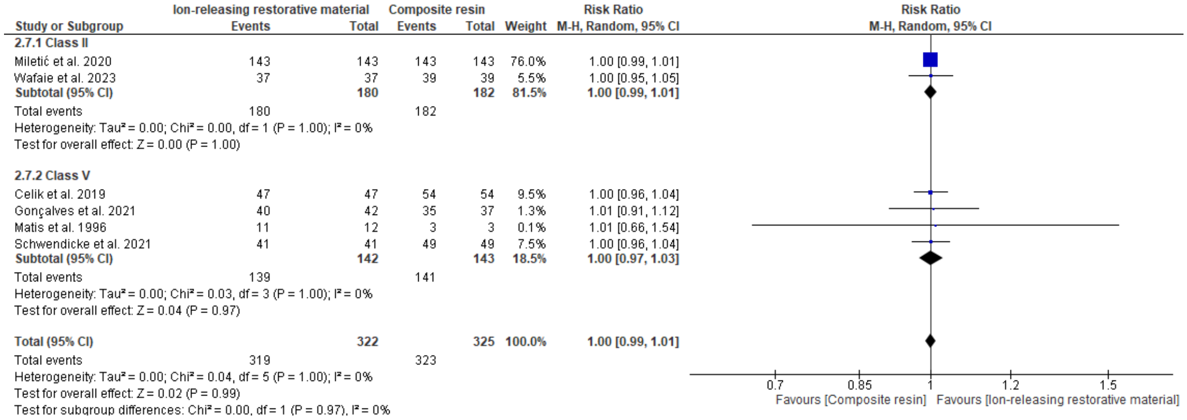

Figure S32. Retention between IRR and CR in dental restorations by cavity types

Study or Subgroup	Ion-releasing restorative material Events	Ion-releasing restorative material Total	Composite resin Events	Composite resin Total	Weight	Risk Ratio M-H, Random, 95% CI
**2.8.1 Class I and II**						
Albelasy et al. 2024	26	27	27	27	4.5%	0.96 [0.87, 1.07]
Toz-Akalin et al. 2023	28	29	29	29	4.7%	0.97 [0.88, 1.06]
van Dijken et al. 2019	62	82	80	82	3.8%	0.78 [0.68, 0.88]
**Subtotal (95% CI)**		**138**		**138**	**13.0%**	**0.90 [0.76, 1.06]**
Total events	116		136			
Heterogeneity: Tau² = 0.02; Chi² = 14.24, df = 2 (P = 0.0008); I² = 86%						
Test for overall effect: Z = 1.25 (P = 0.21)						
**2.8.2 Class II**						
Balkaya et al. 2020	15	21	32	32	1.6%	0.72 [0.54, 0.94]
Miletić et al. 2020	143	143	141	143	6.2%	1.01 [0.99, 1.04]
Wafaie et al. 2023	37	39	39	39	4.8%	0.95 [0.87, 1.04]
**Subtotal (95% CI)**		**203**		**214**	**12.6%**	**0.92 [0.77, 1.10]**
Total events	195		212			
Heterogeneity: Tau² = 0.02; Chi² = 22.01, df = 2 (P < 0.0001); I² = 91%						
Test for overall effect: Z = 0.93 (P = 0.35)						
**2.8.3 Class V**						
Adeleke et al. 2012	131	144	106	143	4.3%	1.23 [1.10, 1.37]
Ballal et al. 2023	24	40	33	40	1.5%	0.73 [0.54, 0.97]
Brackett et al. 2003	26	27	22	27	2.5%	1.18 [0.97, 1.44]
Celik et al. 2019	47	49	54	54	5.3%	0.96 [0.90, 1.03]
de Oliveira et al. 2012	38	40	41	41	4.9%	0.95 [0.87, 1.03]
Fagundes et al. 2014	23	26	13	25	0.9%	1.70 [1.14, 2.54]
Folwaczny et al. 2001	23	26	22	23	3.1%	0.92 [0.79, 1.09]
Franco et al. 2006	27	28	27	33	2.8%	1.18 [0.99, 1.41]
Gonçalves et al. 2021	42	43	37	42	4.0%	1.11 [0.98, 1.25]
Hussainy et al. 2018	32	33	32	34	4.4%	1.03 [0.93, 1.14]
Jassal et al. 2018	90	98	92	98	5.1%	0.98 [0.91, 1.06]
Koc Vural et al. 2021	47	51	43	51	3.5%	1.09 [0.95, 1.26]
Matis et al. 1996	12	18	3	18	0.1%	4.00 [1.35, 11.82]
Onal et al. 2005	24	24	13	18	1.4%	1.38 [1.03, 1.85]
Ozgünaltay et al. 2002	44	45	40	42	5.0%	1.03 [0.95, 1.11]
Perdigão et al. 2012	26	26	25	27	3.9%	1.08 [0.95, 1.22]
Pollington et al. 2008	26	30	26	30	2.5%	1.00 [0.82, 1.22]
Popescu et al. 2016	54	57	53	57	4.7%	1.02 [0.93, 1.12]
Saghir et al. 2023	28	30	19	30	1.5%	1.47 [1.10, 1.97]
Santiago et al. 2010	33	33	26	33	2.7%	1.26 [1.05, 1.52]
Schwendicke et al. 2021	39	41	49	49	5.0%	0.95 [0.88, 1.03]
Türkün et al. 2008	48	50	50	50	5.3%	0.96 [0.90, 1.03]
**Subtotal (95% CI)**		**959**		**965**	**74.4%**	**1.06 [1.00, 1.12]**
Total events	884		826			
Heterogeneity: Tau² = 0.01; Chi² = 89.10, df = 21 (P < 0.00001); I² = 76%						
Test for overall effect: Z = 2.01 (P = 0.04)						
**Total (95% CI)**		**1300**		**1317**	**100.0%**	**1.02 [0.98, 1.06]**
Total events	1195		1174			
Heterogeneity: Tau² = 0.01; Chi² = 103.38, df = 27 (P < 0.00001); I² = 74%						
Test for overall effect: Z = 0.82 (P = 0.41)						
Test for subgroup differences: Chi² = 5.08, df = 2 (P = 0.08), I² = 60.6%						

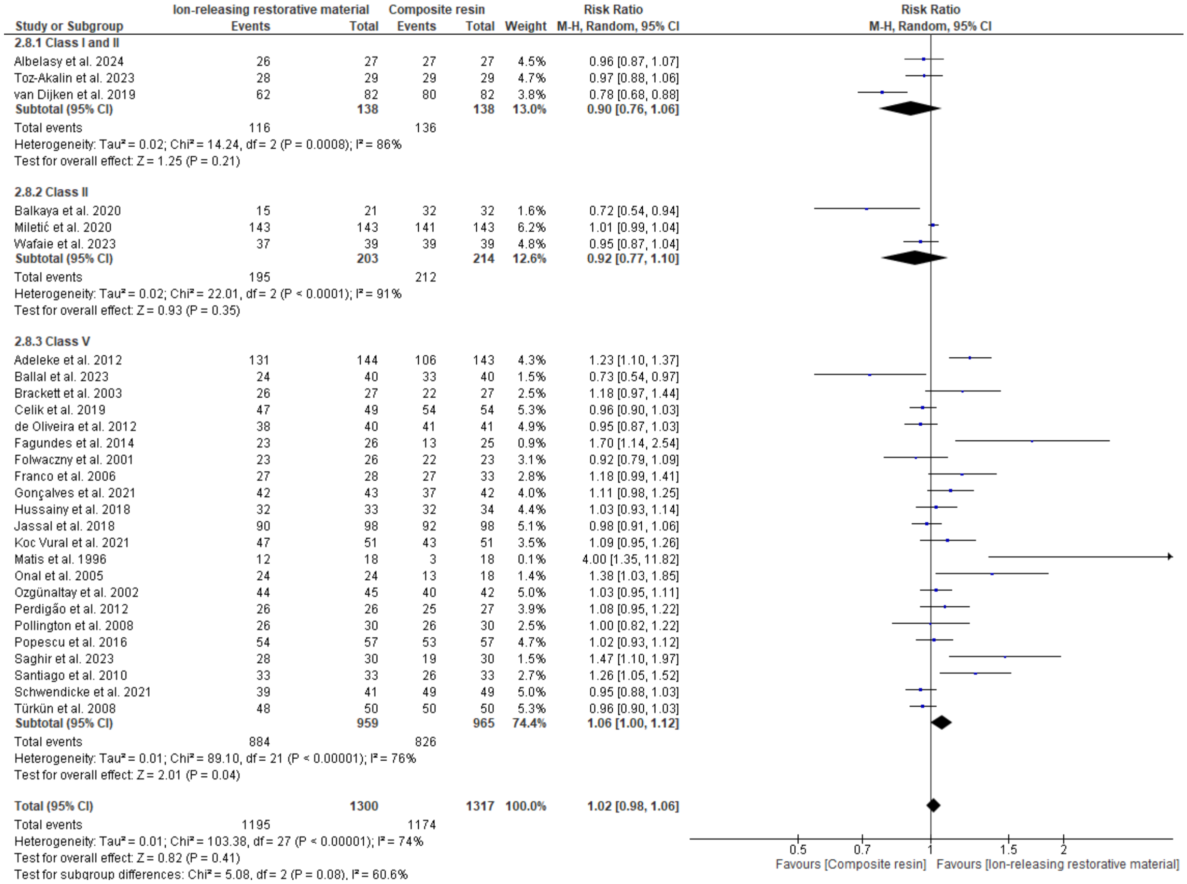

Figure S33. Absence of wear between IRR and CR in dental restorations by cavity types

Study or Subgroup	Ion-releasing restorative material Events	Ion-releasing restorative material Total	Composite resin Events	Composite resin Total	Weight	Risk Ratio M-H, Random, 95% CI
**2.9.1 Class II**						
Miletić et al. 2020	143	143	143	143	50.2%	1.00 [0.99, 1.01]
Wafaie et al. 2023	37	37	39	39	3.6%	1.00 [0.95, 1.05]
**Subtotal (95% CI)**		**180**		**182**	**53.8%**	**1.00 [0.99, 1.01]**
Total events	180		182			
Heterogeneity: Tau² = 0.00; Chi² = 0.00, df = 1 (P = 1.00); I² = 0%						
Test for overall effect: Z = 0.00 (P = 1.00)						
**2.9.2 Class V**						
Adeleke et al. 2012	131	131	106	106	33.4%	1.00 [0.98, 1.02]
Celik et al. 2019	47	47	54	54	6.3%	1.00 [0.96, 1.04]
Gonçalves et al. 2021	39	42	36	37	0.9%	0.95 [0.86, 1.05]
Matis et al. 1996	9	12	3	3	0.0%	0.84 [0.51, 1.37]
Perdigão et al. 2012	26	26	25	27	0.6%	1.08 [0.95, 1.22]
Schwendicke et al. 2021	41	41	49	49	5.0%	1.00 [0.96, 1.04]
**Subtotal (95% CI)**		**299**		**276**	**46.2%**	**1.00 [0.99, 1.01]**
Total events	293		273			
Heterogeneity: Tau² = 0.00; Chi² = 2.78, df = 5 (P = 0.73); I² = 0%						
Test for overall effect: Z = 0.02 (P = 0.98)						
**Total (95% CI)**		**479**		**458**	**100.0%**	**1.00 [0.99, 1.01]**
Total events	473		455			
Heterogeneity: Tau² = 0.00; Chi² = 2.77, df = 7 (P = 0.91); I² = 0%						
Test for overall effect: Z = 0.02 (P = 0.99)						
Test for subgroup differences: Chi² = 0.00, df = 1 (P = 0.99), I² = 0%						

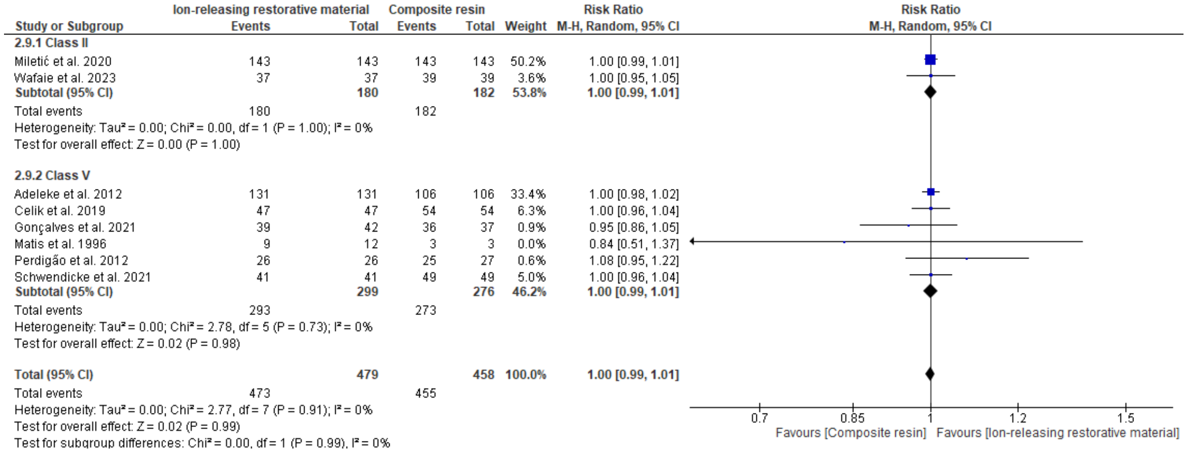

Figure S34. Proper anatomic form between IRR and CR in dental restorations by cavity types

Study or Subgroup	Ion-releasing restorative material Events	Ion-releasing restorative material Total	Composite resin Events	Composite resin Total	Weight	Risk Ratio M-H, Random, 95% CI
**2.10.1 Class II**						
Balkaya et al. 2020	20	21	32	32	4.0%	0.95 [0.84, 1.07]
Miletić et al. 2020	143	143	143	143	10.9%	1.00 [0.99, 1.01]
Wafaie et al. 2023	37	37	39	39	8.5%	1.00 [0.95, 1.05]
**Subtotal (95% CI)**		**201**		**214**	**23.3%**	**1.00 [0.99, 1.01]**
Total events	200		214			
Heterogeneity: Tau² = 0.00; Chi² = 1.63, df = 2 (P = 0.44); I² = 0%						
Test for overall effect: Z = 0.10 (P = 0.92)						
**2.10.2 Class V**						
Ballal et al. 2023	22	40	32	40	0.8%	0.69 [0.50, 0.95]
Brackett et al. 2003	26	26	22	22	6.2%	1.00 [0.92, 1.08]
Celik et al. 2019	45	47	54	54	6.9%	0.96 [0.89, 1.03]
de Oliveira et al. 2012	38	38	41	41	8.6%	1.00 [0.95, 1.05]
Fagundes et al. 2014	21	23	12	13	1.9%	0.99 [0.81, 1.21]
Federlin et al. 1998	15	15	15	15	3.8%	1.00 [0.88, 1.13]
Folwaczny et al. 2001	14	23	22	22	0.8%	0.62 [0.44, 0.86]
Franco et al. 2006	23	27	15	17	1.4%	0.97 [0.76, 1.22]
Gonçalves et al. 2021	40	42	36	37	5.8%	0.98 [0.90, 1.07]
Matis et al. 1996	8	12	3	3	0.3%	0.75 [0.43, 1.28]
Onal et al. 2005	24	24	13	13	4.2%	1.00 [0.89, 1.12]
Ozgünaltay et al. 2002	44	44	40	40	8.8%	1.00 [0.96, 1.05]
Pollington et al. 2008	27	30	29	30	3.4%	0.93 [0.81, 1.07]
Popescu et al. 2016	57	57	53	53	9.7%	1.00 [0.97, 1.04]
Santiago et al. 2010	33	33	25	26	5.0%	1.04 [0.94, 1.15]
Schwendicke et al. 2021	41	41	49	49	9.0%	1.00 [0.96, 1.04]
**Subtotal (95% CI)**		**522**		**475**	**76.7%**	**0.98 [0.95, 1.02]**
Total events	478		461			
Heterogeneity: Tau² = 0.00; Chi² = 42.50, df = 15 (P = 0.0002); I² = 65%						
Test for overall effect: Z = 0.99 (P = 0.32)						
**Total (95% CI)**		**723**		**689**	**100.0%**	**0.98 [0.96, 1.02]**
Total events	678		675			
Heterogeneity: Tau² = 0.00; Chi² = 62.75, df = 18 (P < 0.00001); I² = 71%						
Test for overall effect: Z = 0.99 (P = 0.32)						
Test for subgroup differences: Chi² = 0.81, df = 1 (P = 0.37), I² = 0%						

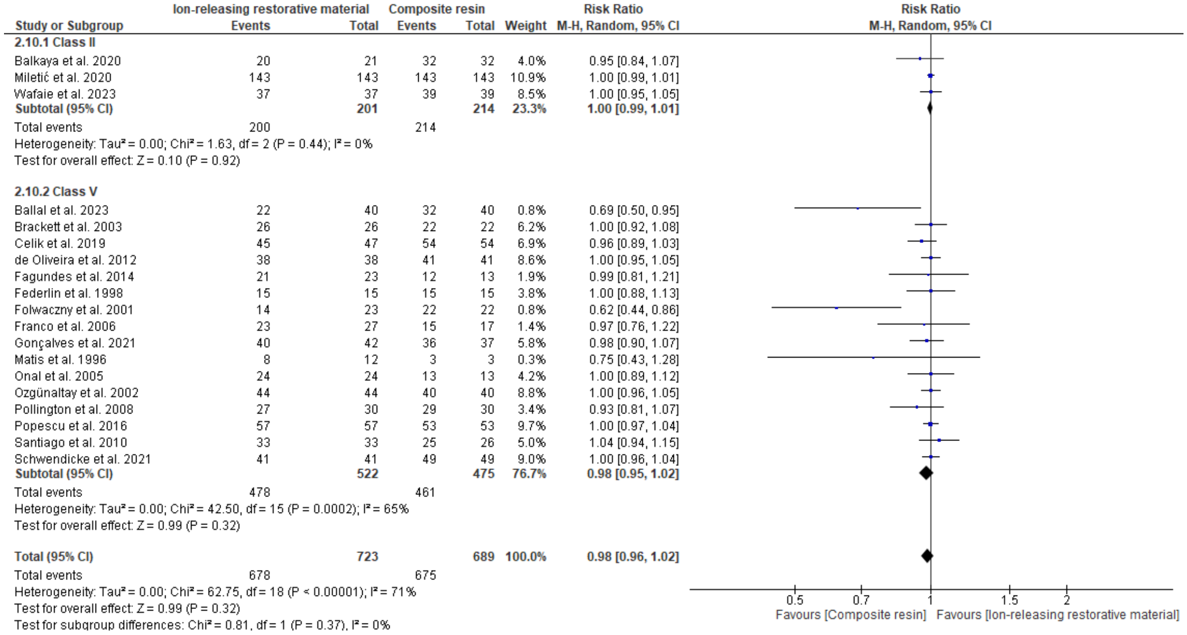

Figure S35. Absence of sensibility between IRR and CR in dental restorations by cavity types

	Ion-releasing restorative material		Composite resin			Risk Ratio
**Study or Subgroup**	**Events**	**Total**	**Events**	**Total**	**Weight**	**M-H, Random, 95% CI**
**2.11.1 Class I and II**						
Albelasy et al. 2024	27	27	27	27	1.4%	1.00 [0.93, 1.07]
Toz-Akalin et al. 2023	28	28	29	29	1.5%	1.00 [0.94, 1.07]
**Subtotal (95% CI)**		**55**		**56**	**2.9%**	**1.00 [0.95, 1.05]**
Total events	55		56			
Heterogeneity: Tau² = 0.00; Chi² = 0.00, df = 1 (P = 1.00); I² = 0%						
Test for overall effect: Z = 0.00 (P = 1.00)						
**2.11.2 Class II**						
Balkaya et al. 2020	21	21	32	32	1.2%	1.00 [0.93, 1.08]
Miletić et al. 2020	143	143	143	143	36.6%	1.00 [0.99, 1.01]
Wafaie et al. 2023	39	39	39	39	2.8%	1.00 [0.95, 1.05]
**Subtotal (95% CI)**		**203**		**214**	**40.5%**	**1.00 [0.99, 1.01]**
Total events	203		214			
Heterogeneity: Tau² = 0.00; Chi² = 0.00, df = 2 (P = 1.00); I² = 0%						
Test for overall effect: Z = 0.00 (P = 1.00)						
**2.11.3 Class V**						
Adeleke et al. 2012	131	131	106	106	24.3%	1.00 [0.98, 1.02]
Celik et al. 2019	47	47	54	54	4.6%	1.00 [0.96, 1.04]
Hussainy et al. 2018	32	33	32	34	0.6%	1.03 [0.93, 1.14]
Jassal et al. 2018	90	90	92	92	14.9%	1.00 [0.98, 1.02]
Koc Vural et al. 2021	47	47	43	43	3.7%	1.00 [0.96, 1.04]
Perdigão et al. 2012	25	26	24	27	0.3%	1.08 [0.93, 1.26]
Schwendicke et al. 2021	41	41	49	49	3.6%	1.00 [0.96, 1.04]
Türkün et al. 2008	50	50	50	50	4.6%	1.00 [0.96, 1.04]
**Subtotal (95% CI)**		**465**		**455**	**56.6%**	**1.00 [0.99, 1.01]**
Total events	463		450			
Heterogeneity: Tau² = 0.00; Chi² = 2.29, df = 7 (P = 0.94); I² = 0%						
Test for overall effect: Z = 0.13 (P = 0.90)						
**Total (95% CI)**		**723**		**725**	**100.0%**	**1.00 [0.99, 1.01]**
Total events	721		720			
Heterogeneity: Tau² = 0.00; Chi² = 2.03, df = 12 (P = 1.00); I² = 0%						
Test for overall effect: Z = 0.10 (P = 0.92)						
Test for subgroup differences: Chi² = 0.01, df = 2 (P = 1.00), I² = 0%						

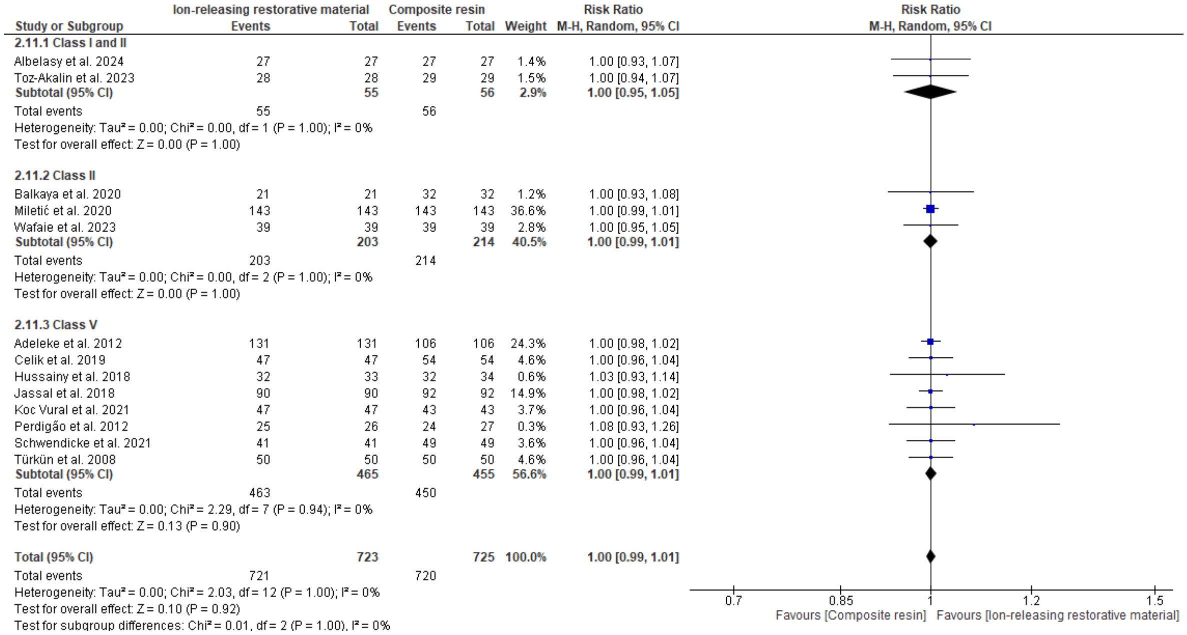

Figure S36. Adequate periodontal tissue between IRR and CR in dental restorations by cavity types

	Ion-releasing restorative material		Composite resin			Risk Ratio
**Study or Subgroup**	**Events**	**Total**	**Events**	**Total**	**Weight**	**M-H, Random, 95% CI**
**2.12.1 Class II**						
Miletić et al. 2020	144	144	144	144	78.3%	1.00 [0.99, 1.01]
Wafaie et al. 2023	39	39	39	39	5.9%	1.00 [0.95, 1.05]
**Subtotal (95% CI)**		**183**		**183**	**84.2%**	**1.00 [0.99, 1.01]**
Total events	183		183			
Heterogeneity: Tau² = 0.00; Chi² = 0.00, df = 1 (P = 1.00); I² = 0%						
Test for overall effect: Z = 0.00 (P = 1.00)						
**2.12.2 Class V**						
Celik et al. 2019	47	47	52	54	3.4%	1.04 [0.97, 1.11]
Gonçalves et al. 2021	42	42	36	37	2.8%	1.03 [0.96, 1.11]
Türkün et al. 2008	50	50	50	50	9.6%	1.00 [0.96, 1.04]
**Subtotal (95% CI)**		**139**		**141**	**15.8%**	**1.01 [0.98, 1.04]**
Total events	139		138			
Heterogeneity: Tau² = 0.00; Chi² = 1.38, df = 2 (P = 0.50); I² = 0%						
Test for overall effect: Z = 0.83 (P = 0.40)						
**Total (95% CI)**		**322**		**324**	**100.0%**	**1.00 [0.99, 1.01]**
Total events	322		321			
Heterogeneity: Tau² = 0.00; Chi² = 3.00, df = 4 (P = 0.56); I² = 0%						
Test for overall effect: Z = 0.33 (P = 0.74)						
Test for subgroup differences: Chi² = 0.59, df = 1 (P = 0.44), I² = 0%						

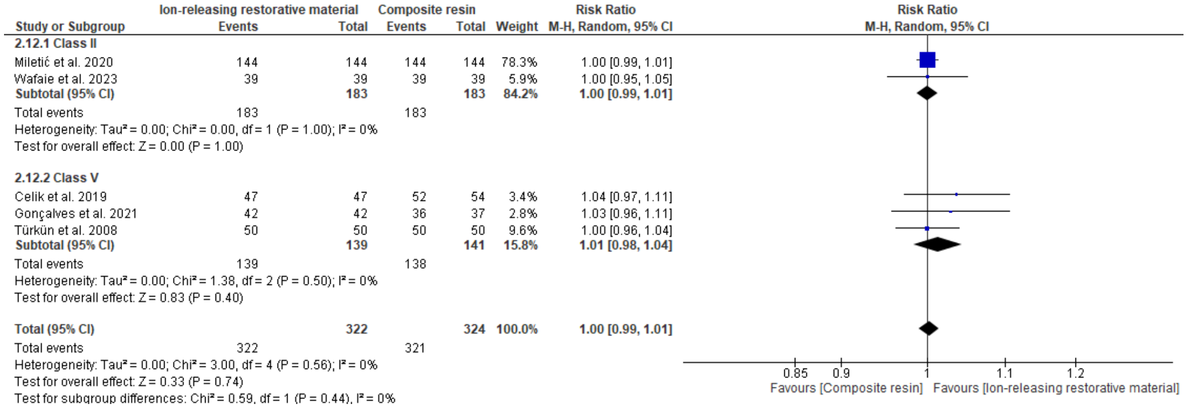

Figure S37. Absence of secondary caries or erosion or abfraction between IRR and CR in dental restorations by the evaluation criteria

Study or Subgroup	Ion-releasing restorative material Events	Ion-releasing restorative material Total	Composite resin Events	Composite resin Total	Weight	Risk Ratio M-H, Random, 95% CI
**4.1.1 FDI**						
Albelasy et al. 2024	27	27	27	27	4.2%	1.00 [0.93, 1.07]
Celik et al. 2019	47	47	54	54	4.8%	1.00 [0.96, 1.04]
Jassal et al. 2018	90	90	92	92	5.0%	1.00 [0.98, 1.02]
Miletić et al. 2020	143	143	143	143	5.1%	1.00 [0.99, 1.01]
Schwendicke et al. 2021	41	41	49	49	4.7%	1.00 [0.96, 1.04]
Toz-Akalin et al. 2023	28	28	28	29	3.6%	1.03 [0.94, 1.14]
Wafaie et al. 2023	37	37	39	39	4.6%	1.00 [0.95, 1.05]
**Subtotal (95% CI)**		**413**		**433**	**31.9%**	**1.00 [0.99, 1.01]**
Total events	413		432			
Heterogeneity: Tau² = 0.00; Chi² = 0.61, df = 6 (P = 1.00); I² = 0%						
Test for overall effect: Z = 0.08 (P = 0.94)						
**4.1.2 USPHS**						
Adeleke et al. 2012	117	117	72	72	5.0%	1.00 [0.98, 1.02]
Balkaya et al. 2020	21	21	32	32	4.0%	1.00 [0.93, 1.08]
Ballal et al. 2023	40	40	40	40	4.6%	1.00 [0.95, 1.05]
Brackett et al. 2003	26	26	22	22	4.0%	1.00 [0.92, 1.08]
de Oliveira et al. 2012	38	38	41	41	4.6%	1.00 [0.95, 1.05]
Fagundes et al. 2014	23	23	12	13	1.9%	1.10 [0.91, 1.33]
Federlin et al. 1998	15	15	15	15	3.0%	1.00 [0.88, 1.13]
Franco et al. 2006	27	27	15	17	1.9%	1.14 [0.94, 1.38]
Gonçalves et al. 2021	42	42	37	37	4.6%	1.00 [0.95, 1.05]
Koc Vural et al. 2021	47	47	43	43	4.7%	1.00 [0.96, 1.04]
Matis et al. 1996	12	12	3	3	0.6%	1.00 [0.68, 1.47]
Onal et al. 2005	24	24	13	13	3.2%	1.00 [0.89, 1.12]
Ozgünaltay et al. 2002	44	44	40	40	4.7%	1.00 [0.96, 1.05]
Perdigão et al. 2012	26	26	25	27	2.9%	1.08 [0.95, 1.22]
Pollington et al. 2008	30	30	30	30	4.3%	1.00 [0.94, 1.07]
Popescu et al. 2016	57	57	53	53	4.8%	1.00 [0.97, 1.04]
Santiago et al. 2010	33	33	26	26	4.3%	1.00 [0.94, 1.07]
Türkün et al. 2008	50	50	50	50	4.8%	1.00 [0.96, 1.04]
van Dijken et al. 2019	3	82	82	82	0.1%	0.04 [0.02, 0.12]
**Subtotal (95% CI)**		**754**		**656**	**68.1%**	**1.00 [0.94, 1.07]**
Total events	675		651			
Heterogeneity: Tau² = 0.02; Chi² = 369.33, df = 18 (P < 0.00001); I² = 95%						
Test for overall effect: Z = 0.06 (P = 0.95)						
**Total (95% CI)**		**1167**		**1089**	**100.0%**	**1.00 [0.97, 1.04]**
Total events	1088		1083			
Heterogeneity: Tau² = 0.01; Chi² = 322.94, df = 25 (P < 0.00001); I² = 92%						
Test for overall effect: Z = 0.26 (P = 0.80)						
Test for subgroup differences: Chi² = 0.00, df = 1 (P = 0.96), I² = 0%						

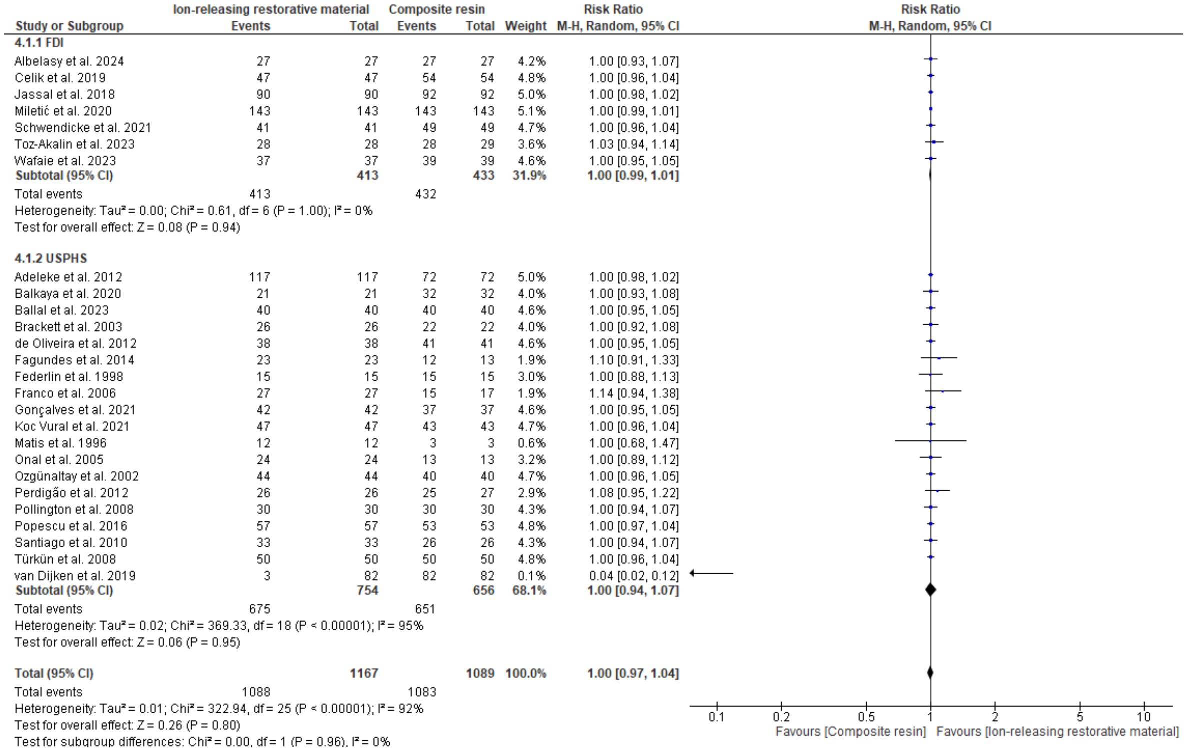

Figure S38. Absence of marginal discoloration between IRR and CR in dental restorations by the evaluation criteria

Study or Subgroup	Ion-releasing restorative material Events	Ion-releasing restorative material Total	Composite resin Events	Composite resin Total	Weight	Risk Ratio M-H, Random, 95% CI
**4.2.1 FDI**						
Albelasy et al. 2024	27	27	27	27	2.7%	1.00 [0.93, 1.07]
Celik et al. 2019	47	47	54	54	6.7%	1.00 [0.96, 1.04]
Jassal et al. 2018	90	90	92	92	11.9%	1.00 [0.98, 1.02]
Miletić et al. 2020	142	142	142	142	14.9%	1.00 [0.99, 1.01]
Schwendicke et al. 2021	41	41	49	49	5.7%	1.00 [0.96, 1.04]
Wafaie et al. 2023	37	37	39	39	4.6%	1.00 [0.95, 1.05]
**Subtotal (95% CI)**		**384**		**403**	**46.4%**	**1.00 [0.99, 1.01]**
Total events	384		403			
Heterogeneity: Tau² = 0.00; Chi² = 0.00, df = 5 (P = 1.00); I² = 0%						
Test for overall effect: Z = 0.00 (P = 1.00)						
**4.2.2 USPHS**						
Adeleke et al. 2012	130	131	105	106	10.9%	1.00 [0.98, 1.03]
Balkaya et al. 2020	20	21	32	32	1.0%	0.95 [0.84, 1.07]
Ballal et al. 2023	24	40	33	40	0.2%	0.73 [0.54, 0.97]
Brackett et al. 2003	26	26	22	22	2.2%	1.00 [0.92, 1.08]
de Oliveira et al. 2012	38	38	41	41	4.8%	1.00 [0.95, 1.05]
Fagundes et al. 2014	23	23	13	13	1.1%	1.00 [0.89, 1.12]
Federlin et al. 1998	15	15	15	15	1.0%	1.00 [0.88, 1.13]
Folwaczny et al. 2001	19	23	20	22	0.3%	0.91 [0.72, 1.14]
Franco et al. 2006	27	27	17	17	1.7%	1.00 [0.91, 1.10]
Gonçalves et al. 2021	29	42	25	37	0.2%	1.02 [0.76, 1.38]
Hussainy et al. 2018	32	33	32	34	1.4%	1.03 [0.93, 1.14]
Koc Vural et al. 2021	31	47	40	43	0.3%	0.71 [0.57, 0.88]
Matis et al. 1996	12	12	3	3	0.1%	1.00 [0.68, 1.47]
Onal et al. 2005	24	24	13	13	1.1%	1.00 [0.89, 1.12]
Ozgünaltay et al. 2002	44	44	40	40	5.3%	1.00 [0.96, 1.05]
Perdigão et al. 2012	25	26	22	27	0.4%	1.18 [0.97, 1.43]
Pollington et al. 2008	28	30	28	30	0.8%	1.00 [0.87, 1.14]
Popescu et al. 2016	32	57	31	53	0.2%	0.96 [0.70, 1.33]
Santiago et al. 2010	33	33	26	26	3.0%	1.00 [0.94, 1.07]
Türkün et al. 2008	50	50	50	50	6.7%	1.00 [0.96, 1.04]
van Dijken et al. 2019	82	82	82	82	11.0%	1.00 [0.98, 1.02]
**Subtotal (95% CI)**		**824**		**746**	**53.6%**	**1.00 [0.97, 1.02]**
Total events	744		690			
Heterogeneity: Tau² = 0.00; Chi² = 35.90, df = 20 (P = 0.02); I² = 44%						
Test for overall effect: Z = 0.39 (P = 0.70)						
**Total (95% CI)**		**1208**		**1149**	**100.0%**	**1.00 [0.99, 1.01]**
Total events	1128		1093			
Heterogeneity: Tau² = 0.00; Chi² = 37.12, df = 26 (P = 0.07); I² = 30%						
Test for overall effect: Z = 0.20 (P = 0.84)						
Test for subgroup differences: Chi² = 0.13, df = 1 (P = 0.72), I² = 0%						

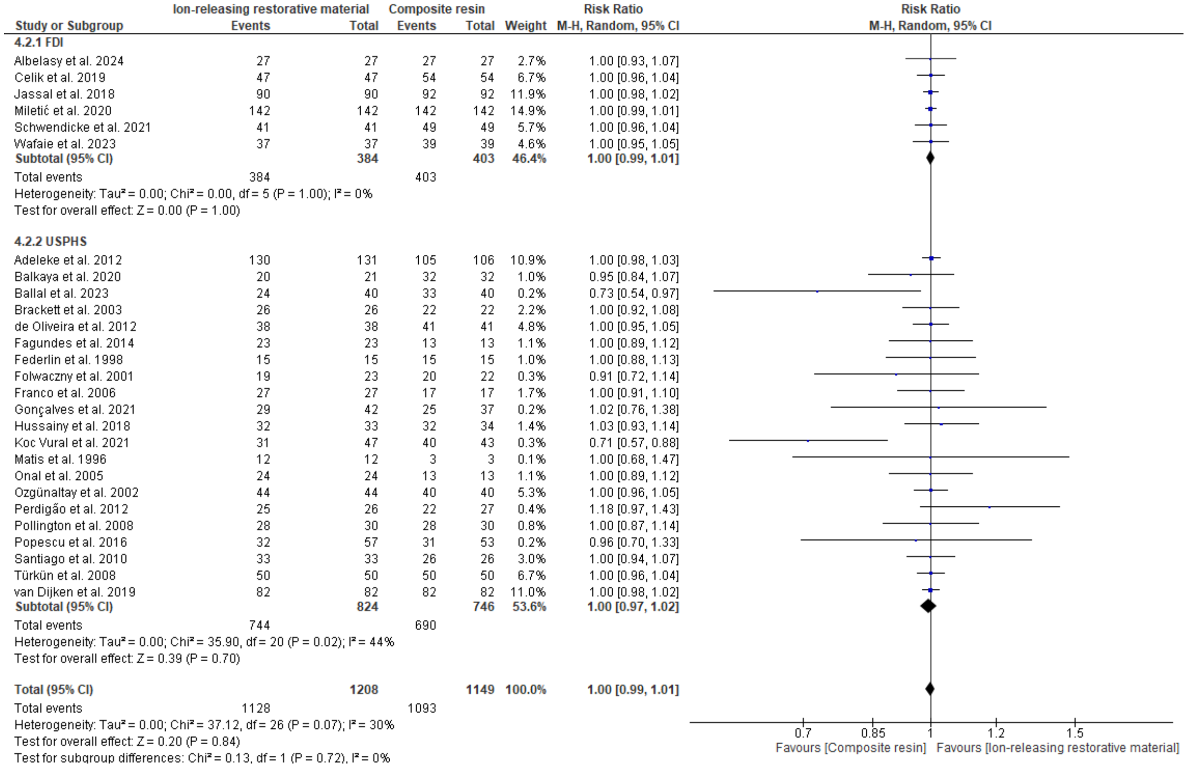

Figure S39. Adequate of marginal adaptation between IRR and CR in dental restorations by the evaluation criteria

Study or Subgroup	Ion-releasing restorative material Events	Ion-releasing restorative material Total	Composite resin Events	Composite resin Total	Weight	Risk Ratio M-H, Random, 95% CI
**4.3.1 FDI**						
Albelasy et al. 2024	27	27	27	27	1.5%	1.00 [0.93, 1.07]
Celik et al. 2019	47	47	54	54	5.0%	1.00 [0.96, 1.04]
Jassal et al. 2018	90	90	92	92	16.2%	1.00 [0.98, 1.02]
Miletić et al. 2020	142	142	142	142	39.3%	1.00 [0.99, 1.01]
Schwendicke et al. 2021	40	41	49	49	1.8%	0.97 [0.91, 1.04]
Toz-Akalin et al. 2023	28	28	29	29	1.6%	1.00 [0.94, 1.07]
Wafaie et al. 2023	37	37	39	39	2.9%	1.00 [0.95, 1.05]
**Subtotal (95% CI)**		**412**		**432**	**68.3%**	**1.00 [0.99, 1.01]**
Total events	411		432			
Heterogeneity: Tau² = 0.00; Chi² = 0.78, df = 6 (P = 0.99); I² = 0%						
Test for overall effect: Z = 0.13 (P = 0.90)						
**4.3.2 USPHS**						
Adeleke et al. 2012	131	131	105	106	11.9%	1.01 [0.99, 1.04]
Balkaya et al. 2020	20	21	32	32	0.5%	0.95 [0.84, 1.07]
Brackett et al. 2003	26	26	22	22	1.2%	1.00 [0.92, 1.08]
de Oliveira et al. 2012	38	38	41	41	3.1%	1.00 [0.95, 1.05]
Hussainy et al. 2018	32	33	32	34	0.7%	1.03 [0.93, 1.14]
Koc Vural et al. 2021	47	47	43	43	4.0%	1.00 [0.96, 1.04]
Matis et al. 1996	10	12	2	3	0.0%	1.25 [0.54, 2.89]
Ozgünaltay et al. 2002	44	44	40	40	3.5%	1.00 [0.96, 1.05]
Perdigão et al. 2012	26	26	23	27	0.3%	1.17 [0.99, 1.39]
Pollington et al. 2008	24	30	25	30	0.1%	0.96 [0.76, 1.22]
Popescu et al. 2016	32	57	30	53	0.1%	0.99 [0.71, 1.38]
Saghir et al. 2023	23	30	21	30	0.1%	1.10 [0.81, 1.49]
Türkün et al. 2008	50	50	50	50	5.0%	1.00 [0.96, 1.04]
van Dijken et al. 2019	74	82	81	82	1.3%	0.91 [0.85, 0.98]
**Subtotal (95% CI)**		**627**		**593**	**31.7%**	**1.00 [0.99, 1.02]**
Total events	577		547			
Heterogeneity: Tau² = 0.00; Chi² = 11.41, df = 13 (P = 0.58); I² = 0%						
Test for overall effect: Z = 0.16 (P = 0.87)						
**Total (95% CI)**		**1039**		**1025**	**100.0%**	**1.00 [0.99, 1.01]**
Total events	988		979			
Heterogeneity: Tau² = 0.00; Chi² = 12.15, df = 20 (P = 0.91); I² = 0%						
Test for overall effect: Z = 0.01 (P = 0.99)						
Test for subgroup differences: Chi² = 0.04, df = 1 (P = 0.83), I² = 0%						

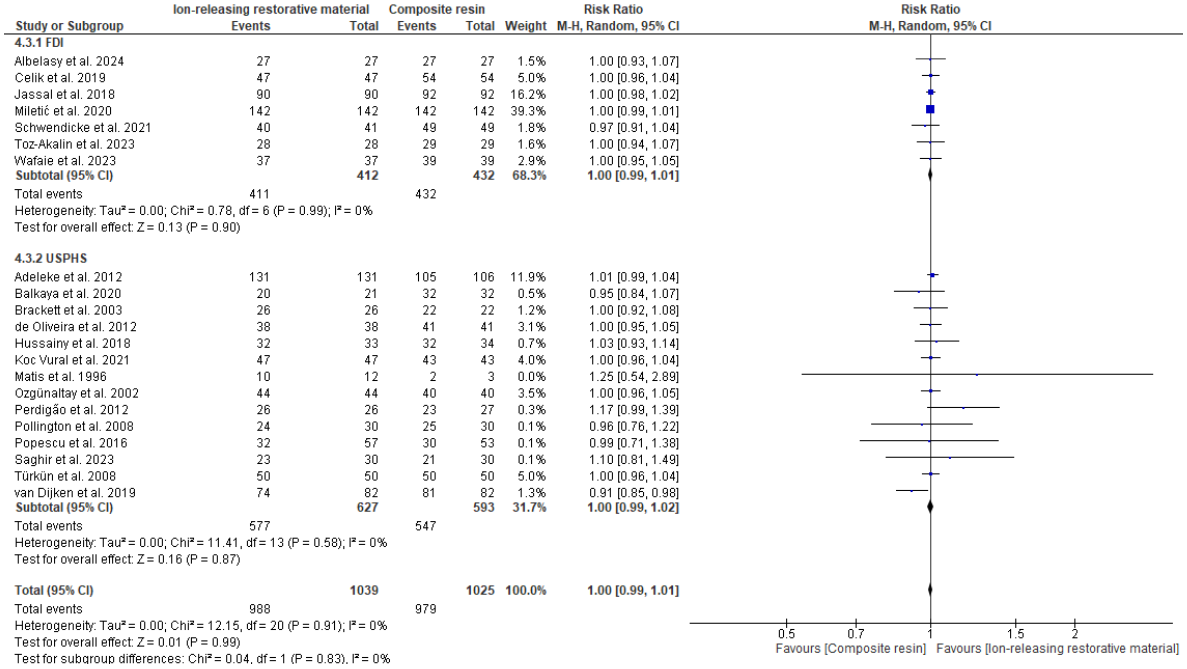

Figure S40. Adequate marginal or tooth integrity between IRR and CR in dental restorations by the evaluation criteria

Study or Subgroup	Ion-releasing restorative material Events	Ion-releasing restorative material Total	Composite resin Events	Composite resin Total	Weight	Risk Ratio M-H, Random, 95% CI
**4.4.1 FDI**						
Albelasy et al. 2024	26	27	27	27	1.4%	0.96 [0.87, 1.07]
Celik et al. 2019	47	47	54	54	9.4%	1.00 [0.96, 1.04]
Miletić et al. 2020	143	143	143	143	75.1%	1.00 [0.99, 1.01]
Toz-Akalin et al. 2023	28	28	29	29	3.1%	1.00 [0.94, 1.07]
Wafaie et al. 2023	39	39	39	39	5.7%	1.00 [0.95, 1.05]
**Subtotal (95% CI)**		**284**		**292**	**94.8%**	**1.00 [0.99, 1.01]**
Total events	283		292			
Heterogeneity: Tau² = 0.00; Chi² = 0.73, df = 4 (P = 0.95); I² = 0%						
Test for overall effect: Z = 0.09 (P = 0.93)						
**4.4.2 USPHS**						
Ballal et al. 2023	23	40	31	40	0.1%	0.74 [0.54, 1.02]
Fagundes et al. 2014	20	23	9	13	0.1%	1.26 [0.85, 1.87]
Federlin et al. 1998	15	15	13	15	0.3%	1.15 [0.91, 1.44]
Folwaczny et al. 2001	17	23	20	22	0.2%	0.81 [0.62, 1.07]
Franco et al. 2006	23	27	13	17	0.1%	1.11 [0.82, 1.51]
Gonçalves et al. 2021	30	42	25	37	0.2%	1.06 [0.79, 1.42]
Onal et al. 2005	24	24	13	13	1.1%	1.00 [0.89, 1.12]
Santiago et al. 2010	33	33	26	26	3.2%	1.00 [0.94, 1.07]
**Subtotal (95% CI)**		**227**		**183**	**5.2%**	**1.00 [0.92, 1.08]**
Total events	185		150			
Heterogeneity: Tau² = 0.00; Chi² = 9.61, df = 7 (P = 0.21); I² = 27%						
Test for overall effect: Z = 0.01 (P = 0.99)						
**Total (95% CI)**		**511**		**475**	**100.0%**	**1.00 [0.99, 1.01]**
Total events	468		442			
Heterogeneity: Tau² = 0.00; Chi² = 11.91, df = 12 (P = 0.45); I² = 0%						
Test for overall effect: Z = 0.08 (P = 0.94)						
Test for subgroup differences: Chi² = 0.00, df = 1 (P = 0.98), I² = 0%						

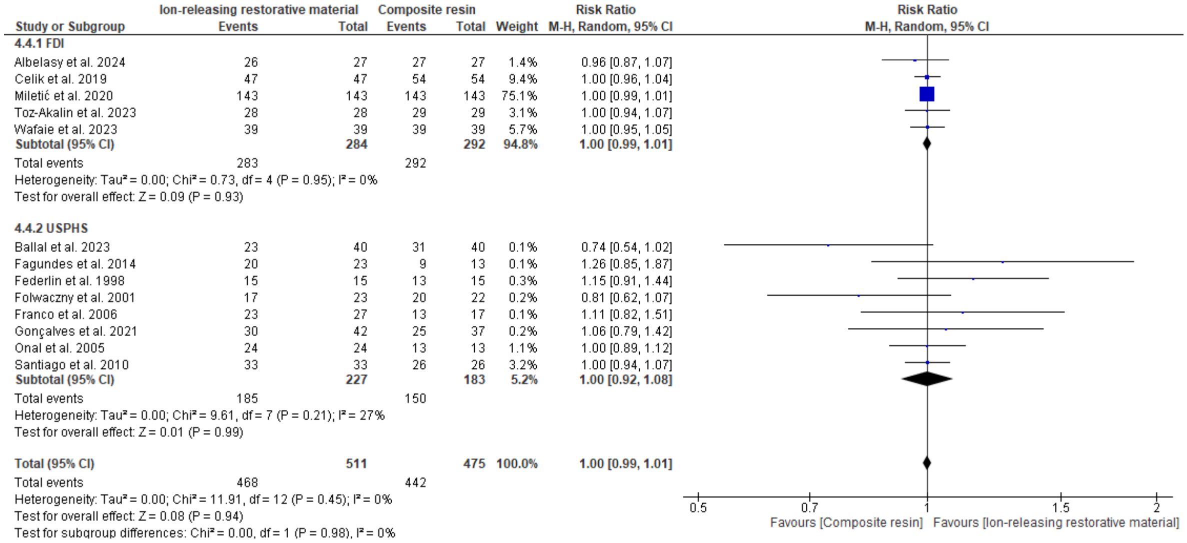

Figure S41. Adequate color or translucency between IRR and CR in dental restorations by the evaluation criteria

Study or Subgroup	Ion-releasing restorative material Events	Total	Composite resin Events	Total	Weight	Risk Ratio M-H, Random, 95% CI
**4.5.1 FDI**						
Celik et al. 2019	47	47	54	54	14.0%	1.00 [0.96, 1.04]
Schwendicke et al. 2021	41	41	49	49	12.3%	1.00 [0.96, 1.04]
Toz-Akalin et al. 2023	28	28	29	29	6.8%	1.00 [0.94, 1.07]
Wafaie et al. 2023	37	37	39	39	10.1%	1.00 [0.95, 1.05]
**Subtotal (95% CI)**		**153**		**171**	**43.1%**	**1.00 [0.98, 1.02]**
Total events	153		171			
Heterogeneity: Tau² = 0.00; Chi² = 0.00, df = 3 (P = 1.00); I² = 0%						
Test for overall effect: Z = 0.00 (P = 1.00)						
**4.5.2 USPHS**						
Balkaya et al. 2020	15	21	32	32	0.5%	0.72 [0.54, 0.94]
Brackett et al. 2003	26	26	22	22	5.1%	1.00 [0.92, 1.08]
de Oliveira et al. 2012	38	38	41	41	10.6%	1.00 [0.95, 1.05]
Federlin et al. 1998	15	15	15	15	2.3%	1.00 [0.88, 1.13]
Folwaczny et al. 2001	23	23	22	22	4.7%	1.00 [0.92, 1.09]
Gonçalves et al. 2021	27	42	29	37	0.5%	0.82 [0.62, 1.09]
Hussainy et al. 2018	32	33	32	34	3.3%	1.03 [0.93, 1.14]
Onal et al. 2005	24	24	13	13	2.7%	1.00 [0.89, 1.12]
Ozgünaltay et al. 2002	44	44	40	40	11.4%	1.00 [0.96, 1.05]
Perdigão et al. 2012	26	26	22	27	1.0%	1.22 [1.01, 1.48]
Popescu et al. 2016	50	57	44	53	1.5%	1.06 [0.90, 1.23]
Türkün et al. 2008	50	50	47	50	5.2%	1.06 [0.98, 1.15]
van Dijken et al. 2019	77	82	82	82	8.0%	0.94 [0.88, 1.00]
**Subtotal (95% CI)**		**481**		**468**	**56.9%**	**1.00 [0.97, 1.03]**
Total events	447		441			
Heterogeneity: Tau² = 0.00; Chi² = 20.33, df = 12 (P = 0.06); I² = 41%						
Test for overall effect: Z = 0.01 (P = 0.99)						
**Total (95% CI)**		**634**		**639**	**100.0%**	**1.00 [0.98, 1.02]**
Total events	600		612			
Heterogeneity: Tau² = 0.00; Chi² = 20.50, df = 16 (P = 0.20); I² = 22%						
Test for overall effect: Z = 0.06 (P = 0.95)						
Test for subgroup differences: Chi² = 0.00, df = 1 (P = 0.99), I² = 0%						

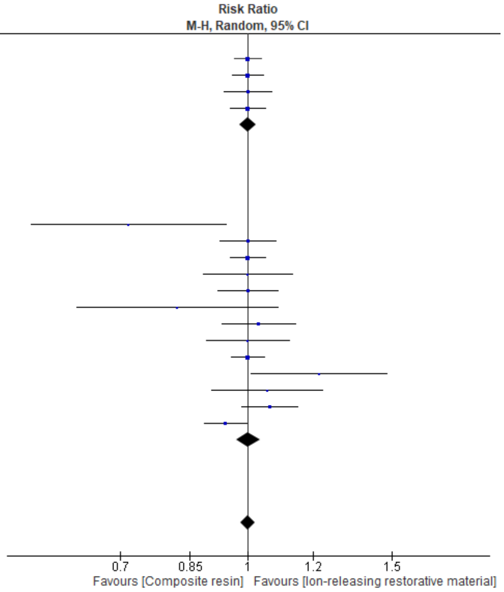

Figure S42. Proper surface texture or luster between IRR and CR in dental restorations by the evaluation criteria

Study or Subgroup	Ion-releasing restorative material Events	Total	Composite resin Events	Total	Weight	Risk Ratio M-H, Random, 95% CI
**4.6.1 FDI**						
Celik et al. 2019	47	47	54	54	12.3%	1.00 [0.96, 1.04]
Schwendicke et al. 2021	41	41	49	49	11.5%	1.00 [0.96, 1.04]
Toz-Akalin et al. 2023	28	28	29	29	8.0%	1.00 [0.94, 1.07]
Wafaie et al. 2023	37	37	39	39	10.3%	1.00 [0.95, 1.05]
**Subtotal (95% CI)**		**153**		**171**	**42.1%**	**1.00 [0.98, 1.02]**
Total events	153		171			
Heterogeneity: Tau² = 0.00; Chi² = 0.00, df = 3 (P = 1.00); I² = 0%						
Test for overall effect: Z = 0.00 (P = 1.00)						
**4.6.2 USPHS**						
Balkaya et al. 2020	19	21	32	32	2.4%	0.90 [0.77, 1.05]
Brackett et al. 2003	26	26	22	22	6.6%	1.00 [0.92, 1.08]
Federlin et al. 1998	15	15	15	15	3.5%	1.00 [0.88, 1.13]
Folwaczny et al. 2001	23	23	22	22	6.2%	1.00 [0.92, 1.09]
Gonçalves et al. 2021	34	42	35	37	2.2%	0.86 [0.73, 1.01]
Hussainy et al. 2018	32	33	32	34	4.7%	1.03 [0.93, 1.14]
Matis et al. 1996	12	12	3	3	0.5%	1.00 [0.68, 1.47]
Onal et al. 2005	24	24	13	13	4.0%	1.00 [0.89, 1.12]
Perdigão et al. 2012	11	26	25	27	0.3%	0.46 [0.29, 0.72]
Saghir et al. 2023	25	30	18	30	0.6%	1.39 [1.00, 1.94]
Türkün et al. 2008	50	50	50	50	12.3%	1.00 [0.96, 1.04]
van Dijken et al. 2019	82	82	82	82	14.7%	1.00 [0.98, 1.02]
**Subtotal (95% CI)**		**384**		**367**	**57.9%**	**0.99 [0.94, 1.03]**
Total events	353		349			
Heterogeneity: Tau² = 0.00; Chi² = 35.22, df = 11 (P = 0.0002); I² = 69%						
Test for overall effect: Z = 0.59 (P = 0.56)						
**Total (95% CI)**		**537**		**538**	**100.0%**	**0.99 [0.97, 1.02]**
Total events	506		520			
Heterogeneity: Tau² = 0.00; Chi² = 31.43, df = 15 (P = 0.008); I² = 52%						
Test for overall effect: Z = 0.38 (P = 0.70)						
Test for subgroup differences: Chi² = 0.28, df = 1 (P = 0.60), I² = 0%						

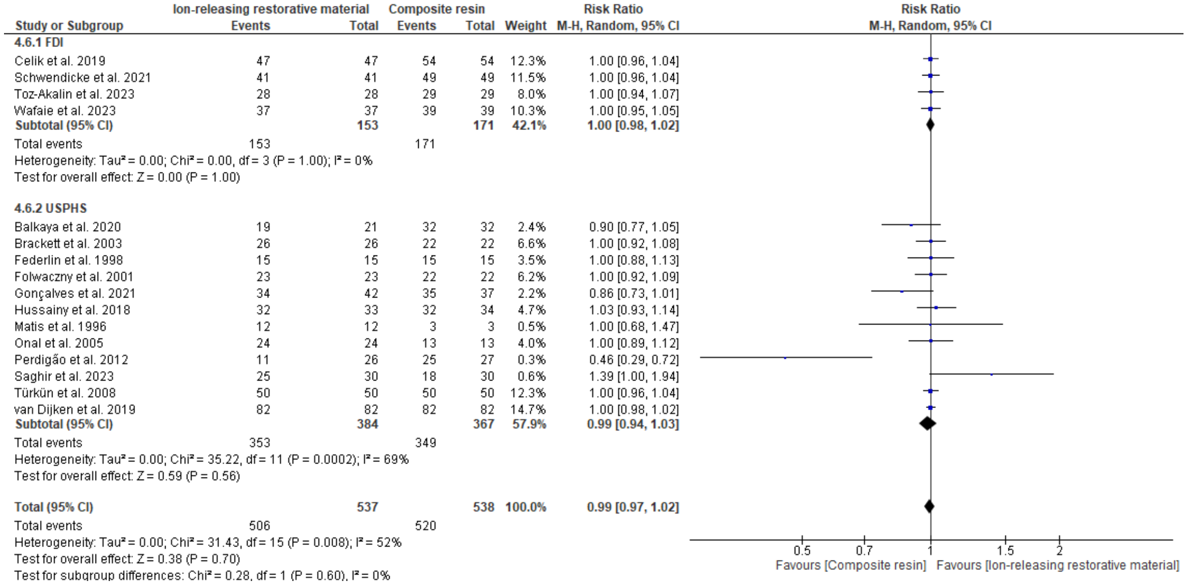

Figure S43. Proper surface staining between IRR and CR in dental restorations by the evaluation criteria

Study or Subgroup	Ion-releasing restorative material Events	Ion-releasing restorative material Total	Composite resin Events	Composite resin Total	Weight	Risk Ratio M-H, Random, 95% CI
**4.7.1 FDI**						
Celik et al. 2019	47	47	54	54	9.2%	1.00 [0.96, 1.04]
Miletić et al. 2020	143	143	143	143	73.7%	1.00 [0.99, 1.01]
Schwendicke et al. 2021	41	41	49	49	7.3%	1.00 [0.96, 1.04]
Toz-Akalin et al. 2023	28	28	29	29	3.0%	1.00 [0.94, 1.07]
Wafaie et al. 2023	37	37	39	39	5.3%	1.00 [0.95, 1.05]
**Subtotal (95% CI)**		**296**		**314**	**98.6%**	**1.00 [0.99, 1.01]**
Total events	296		314			
Heterogeneity: Tau² = 0.00; Chi² = 0.00, df = 4 (P = 1.00); I² = 0%						
Test for overall effect: Z = 0.00 (P = 1.00)						
**4.7.2 USPHS**						
Gonçalves et al. 2021	40	42	35	37	1.3%	1.01 [0.91, 1.12]
Matis et al. 1996	11	12	3	3	0.1%	1.01 [0.66, 1.54]
**Subtotal (95% CI)**		**54**		**40**	**1.4%**	**1.01 [0.91, 1.11]**
Total events	51		38			
Heterogeneity: Tau² = 0.00; Chi² = 0.00, df = 1 (P = 0.98); I² = 0%						
Test for overall effect: Z = 0.14 (P = 0.89)						
**Total (95% CI)**		**350**		**354**	**100.0%**	**1.00 [0.99, 1.01]**
Total events	347		352			
Heterogeneity: Tau² = 0.00; Chi² = 0.04, df = 6 (P = 1.00); I² = 0%						
Test for overall effect: Z = 0.02 (P = 0.99)						
Test for subgroup differences: Chi² = 0.02, df = 1 (P = 0.89), I² = 0%						

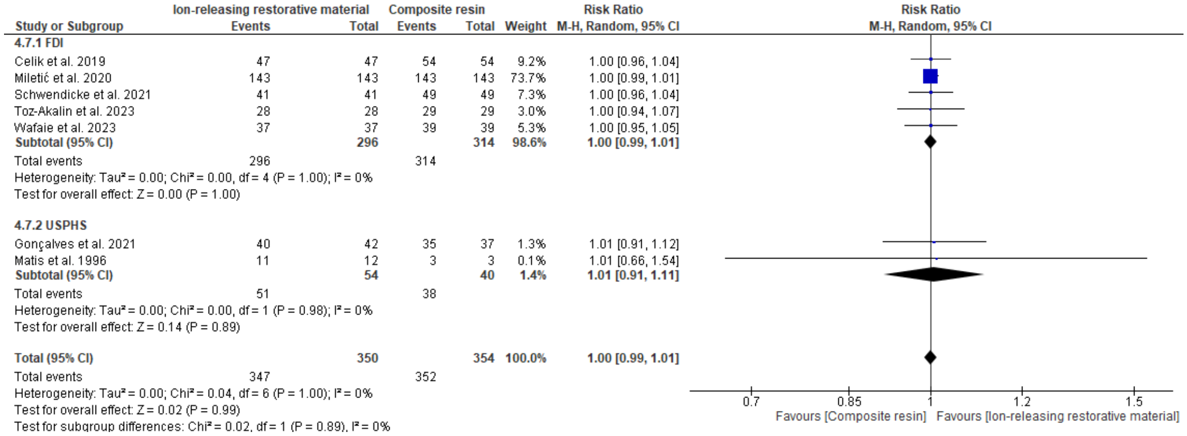

Figure S44. Retention between IRR and CR in dental restorations by the evaluation criteria

Study or Subgroup	Ion-releasing restorative material Events	Ion-releasing restorative material Total	Composite resin Events	Composite resin Total	Weight	Risk Ratio M-H, Random, 95% CI
**4.8.1 FDI**						
Albelasy et al. 2024	26	27	27	27	4.5%	0.96 [0.87, 1.07]
Celik et al. 2019	47	49	54	54	5.3%	0.96 [0.90, 1.03]
Jassal et al. 2018	90	98	92	98	5.1%	0.98 [0.91, 1.06]
Miletić et al. 2020	143	143	141	143	6.2%	1.01 [0.99, 1.04]
Schwendicke et al. 2021	39	41	49	49	5.0%	0.95 [0.88, 1.03]
Toz-Akalin et al. 2023	28	29	29	29	4.7%	0.97 [0.88, 1.06]
Wafaie et al. 2023	37	39	39	39	4.8%	0.95 [0.87, 1.04]
**Subtotal (95% CI)**		**426**		**439**	**35.6%**	**0.98 [0.95, 1.01]**
Total events	410		431			
Heterogeneity: Tau² = 0.00; Chi² = 9.54, df = 6 (P = 0.15); I² = 37%						
Test for overall effect: Z = 1.30 (P = 0.19)						
**4.8.2 USPHS**						
Adeleke et al. 2012	131	144	106	143	4.3%	1.23 [1.10, 1.37]
Balkaya et al. 2020	15	21	32	32	1.6%	0.72 [0.54, 0.94]
Ballal et al. 2023	24	40	33	40	1.5%	0.73 [0.54, 0.97]
Brackett et al. 2003	26	27	22	27	2.5%	1.18 [0.97, 1.44]
de Oliveira et al. 2012	38	40	41	41	4.9%	0.95 [0.87, 1.03]
Fagundes et al. 2014	23	26	13	25	0.9%	1.70 [1.14, 2.54]
Folwaczny et al. 2001	23	26	22	23	3.1%	0.92 [0.79, 1.09]
Franco et al. 2006	27	28	27	33	2.8%	1.18 [0.99, 1.41]
Gonçalves et al. 2021	42	43	37	42	4.0%	1.11 [0.98, 1.25]
Hussainy et al. 2018	32	33	32	34	4.4%	1.03 [0.93, 1.14]
Koc Vural et al. 2021	47	51	43	51	3.5%	1.09 [0.95, 1.26]
Matis et al. 1996	12	18	3	18	0.1%	4.00 [1.35, 11.82]
Onal et al. 2005	24	24	13	18	1.4%	1.38 [1.03, 1.85]
Ozgünaltay et al. 2002	44	45	40	42	5.0%	1.03 [0.95, 1.11]
Perdigão et al. 2012	26	26	25	27	3.9%	1.08 [0.95, 1.22]
Pollington et al. 2008	26	30	26	30	2.5%	1.00 [0.82, 1.22]
Popescu et al. 2016	54	57	53	57	4.7%	1.02 [0.93, 1.12]
Saghir et al. 2023	28	30	19	30	1.5%	1.47 [1.10, 1.97]
Santiago et al. 2010	33	33	26	33	2.7%	1.26 [1.05, 1.52]
Türkün et al. 2008	48	50	50	50	5.3%	0.96 [0.90, 1.03]
van Dijken et al. 2019	62	82	80	82	3.8%	0.78 [0.68, 0.88]
**Subtotal (95% CI)**		**874**		**878**	**64.4%**	**1.05 [0.98, 1.13]**
Total events	785		743			
Heterogeneity: Tau² = 0.02; Chi² = 90.02, df = 20 (P < 0.00001); I² = 78%						
Test for overall effect: Z = 1.50 (P = 0.13)						
**Total (95% CI)**		**1300**		**1317**	**100.0%**	**1.02 [0.98, 1.06]**
Total events	1195		1174			
Heterogeneity: Tau² = 0.01; Chi² = 103.38, df = 27 (P < 0.00001); I² = 74%						
Test for overall effect: Z = 0.82 (P = 0.41)						
Test for subgroup differences: Chi² = 3.67, df = 1 (P = 0.06), I² = 72.7%						

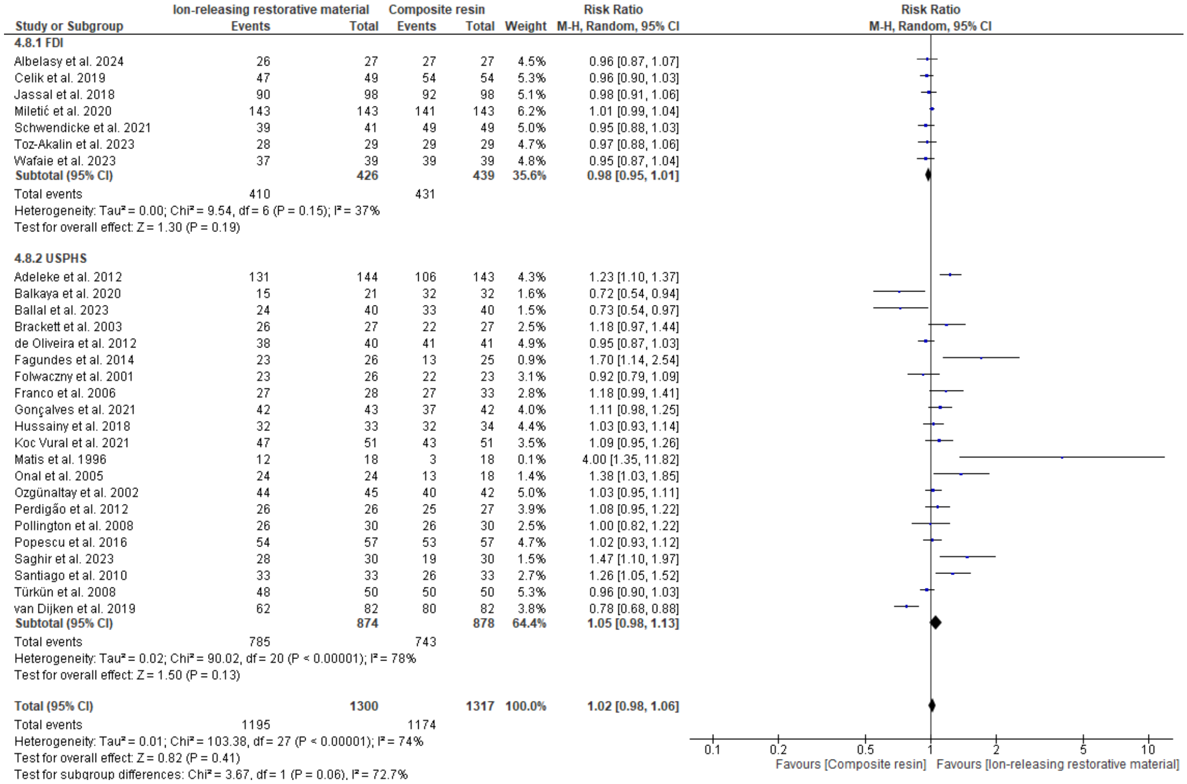

Figure S45. Absence of wear between IRR and CR in dental restorations by the evaluation criteria

Study or Subgroup	Ion-releasing restorative material Events	Ion-releasing restorative material Total	Composite resin Events	Composite resin Total	Weight	Risk Ratio M-H, Random, 95% CI
**4.9.1 FDI**						
Celik et al. 2019	47	47	54	54	6.2%	1.00 [0.96, 1.04]
Miletić et al. 2020	143	143	143	143	49.1%	1.00 [0.99, 1.01]
Schwendicke et al. 2021	41	41	49	49	4.9%	1.00 [0.96, 1.04]
Toz-Akalin et al. 2023	28	28	29	29	2.0%	1.00 [0.94, 1.07]
Wafaie et al. 2023	37	37	39	39	3.6%	1.00 [0.95, 1.05]
**Subtotal (95% CI)**		**296**		**314**	**65.8%**	**1.00 [0.99, 1.01]**
Total events	296		314			
Heterogeneity: Tau² = 0.00; Chi² = 0.00, df = 4 (P = 1.00); I² = 0%						
Test for overall effect: Z = 0.00 (P = 1.00)						
**4.9.2 USPHS**						
Adeleke et al. 2012	131	131	106	106	32.7%	1.00 [0.98, 1.02]
Gonçalves et al. 2021	39	42	36	37	0.9%	0.95 [0.86, 1.05]
Matis et al. 1996	9	12	3	3	0.0%	0.84 [0.51, 1.37]
Perdigão et al. 2012	26	26	25	27	0.6%	1.08 [0.95, 1.22]
**Subtotal (95% CI)**		**211**		**173**	**34.2%**	**1.00 [0.98, 1.02]**
Total events	205		170			
Heterogeneity: Tau² = 0.00; Chi² = 2.86, df = 3 (P = 0.41); I² = 0%						
Test for overall effect: Z = 0.03 (P = 0.98)						
**Total (95% CI)**		**507**		**487**	**100.0%**	**1.00 [0.99, 1.01]**
Total events	501		484			
Heterogeneity: Tau² = 0.00; Chi² = 2.76, df = 8 (P = 0.95); I² = 0%						
Test for overall effect: Z = 0.02 (P = 0.99)						
Test for subgroup differences: Chi² = 0.00, df = 1 (P = 0.98), I² = 0%						

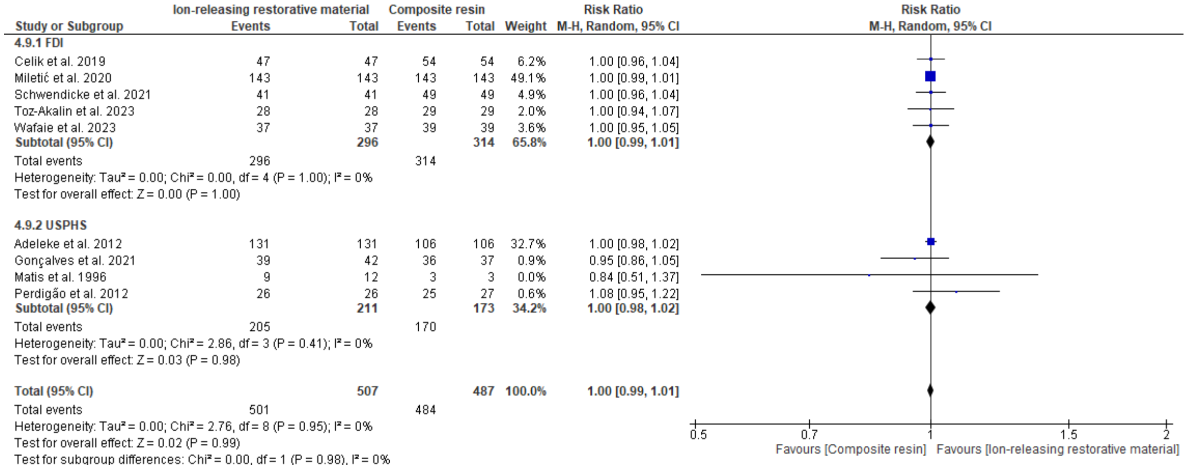

Figure S46. Proper anatomic form between IRR and CR in dental restorations by the evaluation criteria

Study or Subgroup	Ion-releasing restorative material Events	Ion-releasing restorative material Total	Composite resin Events	Composite resin Total	Weight	Risk Ratio M-H, Random, 95% CI
**4.10.1 FDI**						
Celik et al. 2019	45	47	54	54	6.5%	0.96 [0.89, 1.03]
Miletić et al. 2020	143	143	143	143	9.5%	1.00 [0.99, 1.01]
Schwendicke et al. 2021	41	41	49	49	8.2%	1.00 [0.96, 1.04]
Wafaie et al. 2023	37	37	39	39	7.7%	1.00 [0.95, 1.05]
**Subtotal (95% CI)**		**268**		**285**	**31.8%**	**1.00 [0.99, 1.01]**
Total events	266		285			
Heterogeneity: Tau² = 0.00; Chi² = 2.54, df = 3 (P = 0.47); I² = 0%						
Test for overall effect: Z = 0.21 (P = 0.83)						
**4.10.2 USPHS**						
Balkaya et al. 2020	20	21	32	32	4.0%	0.95 [0.84, 1.07]
Ballal et al. 2023	22	40	32	40	0.9%	0.69 [0.50, 0.95]
Brackett et al. 2003	26	26	22	22	6.0%	1.00 [0.92, 1.08]
de Oliveira et al. 2012	38	38	41	41	7.8%	1.00 [0.95, 1.05]
Fagundes et al. 2014	21	23	12	13	2.0%	0.99 [0.81, 1.21]
Federlin et al. 1998	15	15	15	15	3.9%	1.00 [0.88, 1.13]
Folwaczny et al. 2001	14	23	22	22	0.8%	0.62 [0.44, 0.86]
Franco et al. 2006	23	27	15	17	1.5%	0.97 [0.76, 1.22]
Gonçalves et al. 2021	40	42	36	37	5.6%	0.98 [0.90, 1.07]
Matis et al. 1996	8	12	3	3	0.3%	0.75 [0.43, 1.28]
Onal et al. 2005	24	24	13	13	4.2%	1.00 [0.89, 1.12]
Ozgünaltay et al. 2002	44	44	40	40	8.0%	1.00 [0.96, 1.05]
Pollington et al. 2008	27	30	29	30	3.4%	0.93 [0.81, 1.07]
Popescu et al. 2016	57	57	53	53	8.6%	1.00 [0.97, 1.04]
Santiago et al. 2010	33	33	25	26	4.9%	1.04 [0.94, 1.15]
van Dijken et al. 2019	74	82	81	82	6.2%	0.91 [0.85, 0.98]
**Subtotal (95% CI)**		**537**		**486**	**68.2%**	**0.97 [0.93, 1.01]**
Total events	486		471			
Heterogeneity: Tau² = 0.00; Chi² = 48.94, df = 15 (P < 0.0001); I² = 69%						
Test for overall effect: Z = 1.38 (P = 0.17)						
**Total (95% CI)**		**805**		**771**	**100.0%**	**0.98 [0.95, 1.01]**
Total events	752		756			
Heterogeneity: Tau² = 0.00; Chi² = 78.25, df = 19 (P < 0.00001); I² = 76%						
Test for overall effect: Z = 1.33 (P = 0.18)						
Test for subgroup differences: Chi² = 1.60, df = 1 (P = 0.21), I² = 37.7%						

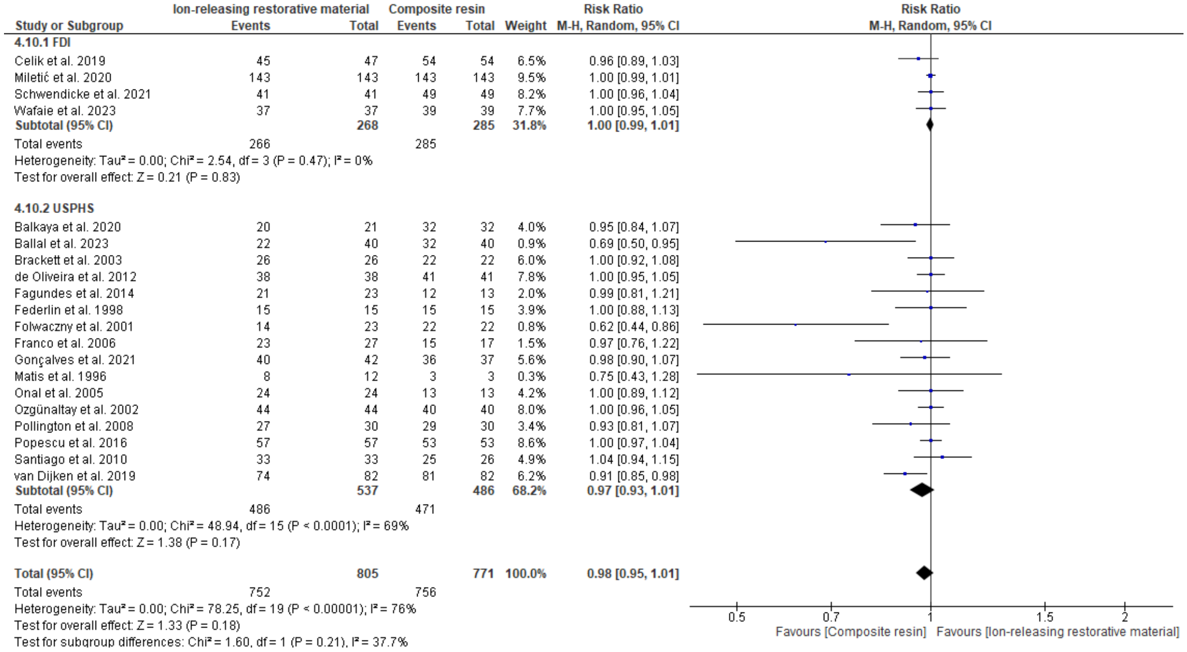

Figure S47. Absence of sensibility between IRR and CR in dental restorations by the evaluation criteria

Study or Subgroup	Ion-releasing restorative material Events	Total	Composite resin Events	Total	Weight	Risk Ratio M-H, Random, 95% CI
**4.11.1 FDI**						
Albelasy et al. 2024	27	27	27	27	1.4%	1.00 [0.93, 1.07]
Celik et al. 2019	47	47	54	54	4.6%	1.00 [0.96, 1.04]
Miletić et al. 2020	143	143	143	143	36.6%	1.00 [0.99, 1.01]
Schwendicke et al. 2021	41	41	49	49	3.6%	1.00 [0.96, 1.04]
Toz-Akalin et al. 2023	28	28	29	29	1.5%	1.00 [0.94, 1.07]
Wafaie et al. 2023	39	39	39	39	2.8%	1.00 [0.95, 1.05]
**Subtotal (95% CI)**		**325**		**341**	**50.4%**	**1.00 [0.99, 1.01]**
Total events	325		341			
Heterogeneity: Tau² = 0.00; Chi² = 0.00, df = 5 (P = 1.00); I² = 0%						
Test for overall effect: Z = 0.00 (P = 1.00)						
**4.11.2 USPHS**						
Adeleke et al. 2012	131	131	106	106	24.3%	1.00 [0.98, 1.02]
Balkaya et al. 2020	21	21	32	32	1.2%	1.00 [0.93, 1.08]
Hussainy et al. 2018	32	33	32	34	0.6%	1.03 [0.93, 1.14]
Jassal et al. 2018	90	90	92	92	14.9%	1.00 [0.98, 1.02]
Koc Vural et al. 2021	47	47	43	43	3.7%	1.00 [0.96, 1.04]
Perdigão et al. 2012	25	26	24	27	0.3%	1.08 [0.93, 1.26]
Türkün et al. 2008	50	50	50	50	4.6%	1.00 [0.96, 1.04]
**Subtotal (95% CI)**		**398**		**384**	**49.6%**	**1.00 [0.99, 1.01]**
Total events	396		379			
Heterogeneity: Tau² = 0.00; Chi² = 2.52, df = 6 (P = 0.87); I² = 0%						
Test for overall effect: Z = 0.14 (P = 0.89)						
**Total (95% CI)**		**723**		**725**	**100.0%**	**1.00 [0.99, 1.01]**
Total events	721		720			
Heterogeneity: Tau² = 0.00; Chi² = 2.03, df = 12 (P = 1.00); I² = 0%						
Test for overall effect: Z = 0.10 (P = 0.92)						
Test for subgroup differences: Chi² = 0.01, df = 1 (P = 0.92), I² = 0%						

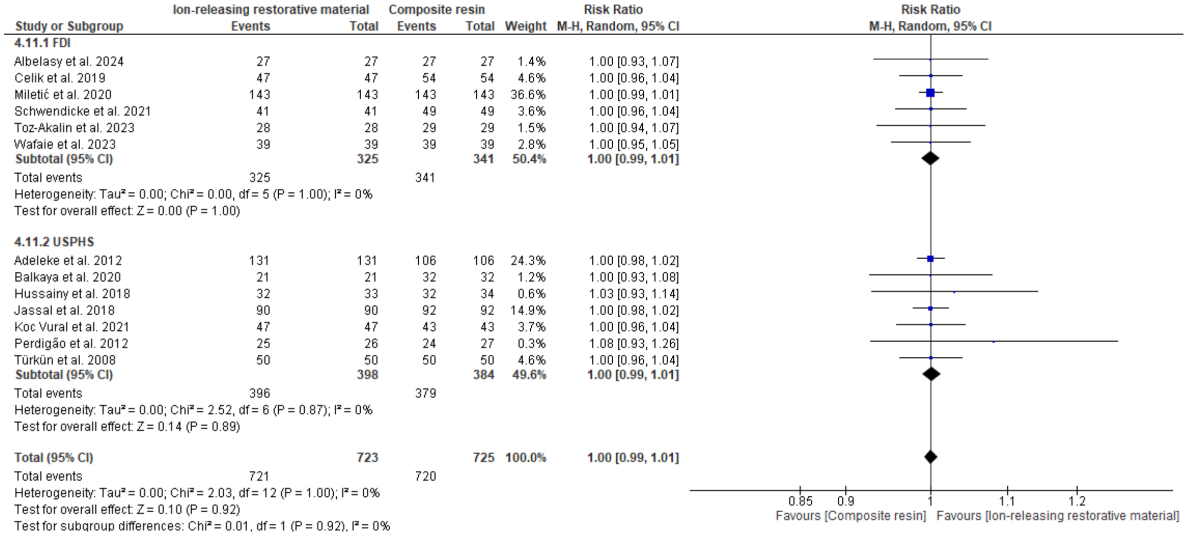

Figure S48. Adequate periodontal tissue between IRR and CR in dental restorations by the evaluation criteria

Study or Subgroup	Ion-releasing restorative material Events	Total	Composite resin Events	Total	Weight	Risk Ratio M-H, Random, 95% CI
**4.12.1 FDI**						
Celik et al. 2019	47	47	52	54	3.4%	1.04 [0.97, 1.11]
Miletić et al. 2020	144	144	144	144	78.3%	1.00 [0.99, 1.01]
Wafaie et al. 2023	39	39	39	39	5.9%	1.00 [0.95, 1.05]
**Subtotal (95% CI)**		**230**		**237**	**87.6%**	**1.00 [0.99, 1.02]**
Total events	230		235			
Heterogeneity: Tau² = 0.00; Chi² = 2.07, df = 2 (P = 0.36); I² = 3%						
Test for overall effect: Z = 0.24 (P = 0.81)						
**4.12.2 USPHS**						
Gonçalves et al. 2021	42	42	36	37	2.8%	1.03 [0.96, 1.11]
Türkün et al. 2008	50	50	50	50	9.6%	1.00 [0.96, 1.04]
**Subtotal (95% CI)**		**92**		**87**	**12.4%**	**1.01 [0.97, 1.04]**
Total events	92		86			
Heterogeneity: Tau² = 0.00; Chi² = 0.59, df = 1 (P = 0.44); I² = 0%						
Test for overall effect: Z = 0.37 (P = 0.71)						
**Total (95% CI)**		**322**		**324**	**100.0%**	**1.00 [0.99, 1.01]**
Total events	322		321			
Heterogeneity: Tau² = 0.00; Chi² = 3.00, df = 4 (P = 0.56); I² = 0%						
Test for overall effect: Z = 0.33 (P = 0.74)						
Test for subgroup differences: Chi² = 0.06, df = 1 (P = 0.81), I² = 0%						

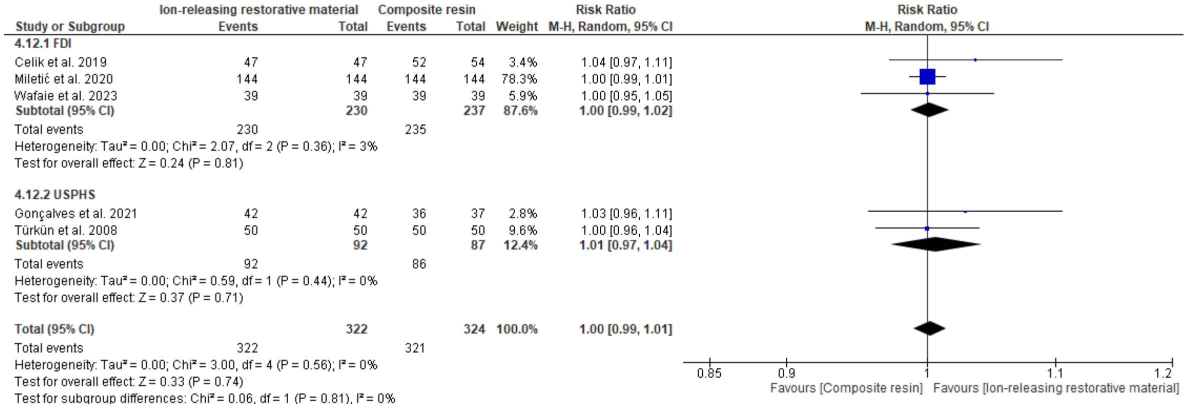

Figure S49. Absence of secondary caries or erosion or abfraction between IRR and CR in dental restorations

by follow-up time

Study or Subgroup	Ion-releasing restorative material Events	Ion-releasing restorative material Total	Composite resin Events	Composite resin Total	Weight	Risk Ratio M-H, Random, 95% CI
**5.1.1 1 year**						
Adeleke et al. 2012	117	117	72	72	5.2%	1.00 [0.98, 1.02]
Ballal et al. 2023	40	40	40	40	4.9%	1.00 [0.95, 1.05]
de Oliveira et al. 2012	38	38	41	41	4.9%	1.00 [0.95, 1.05]
Federlin et al. 1998	15	15	15	15	3.5%	1.00 [0.88, 1.13]
Perdigão et al. 2012	26	26	25	27	3.4%	1.08 [0.95, 1.22]
van Dijken et al. 2019	3	82	82	82	0.1%	0.04 [0.02, 0.12]
**Subtotal (95% CI)**		**318**		**277**	**22.1%**	**0.80 [0.55, 1.15]**
Total events	239		275			
Heterogeneity: Tau² = 0.19; Chi² = 1104.72, df = 5 (P < 0.00001); I² = 100%						
Test for overall effect: Z = 1.21 (P = 0.23)						
**5.1.2 2 years**						
Albelasy et al. 2024	27	27	27	27	4.5%	1.00 [0.93, 1.07]
Balkaya et al. 2020	21	21	32	32	4.4%	1.00 [0.93, 1.08]
Brackett et al. 2003	26	26	22	22	4.4%	1.00 [0.92, 1.08]
Miletić et al. 2020	143	143	143	143	5.3%	1.00 [0.99, 1.01]
Onal et al. 2005	24	24	13	13	3.6%	1.00 [0.89, 1.12]
Popescu et al. 2016	57	57	53	53	5.1%	1.00 [0.97, 1.04]
Santiago et al. 2010	33	33	26	26	4.6%	1.00 [0.94, 1.07]
Toz-Akalin et al. 2023	28	28	28	29	4.1%	1.03 [0.94, 1.14]
Türkün et al. 2008	50	50	50	50	5.1%	1.00 [0.96, 1.04]
**Subtotal (95% CI)**		**409**		**395**	**41.1%**	**1.00 [0.99, 1.01]**
Total events	409		394			
Heterogeneity: Tau² = 0.00; Chi² = 0.59, df = 8 (P = 1.00); I² = 0%						
Test for overall effect: Z = 0.08 (P = 0.93)						
**5.1.3 3 years**						
Celik et al. 2019	47	47	54	54	5.1%	1.00 [0.96, 1.04]
Gonçalves et al. 2021	42	42	37	37	4.9%	1.00 [0.95, 1.05]
Koc Vural et al. 2021	47	47	43	43	5.0%	1.00 [0.96, 1.04]
Ozgünaltay et al. 2002	44	44	40	40	5.0%	1.00 [0.96, 1.05]
Pollington et al. 2008	30	30	30	30	4.7%	1.00 [0.94, 1.07]
Schwendicke et al. 2021	41	41	49	49	5.0%	1.00 [0.96, 1.04]
**Subtotal (95% CI)**		**251**		**253**	**29.6%**	**1.00 [0.98, 1.02]**
Total events	251		253			
Heterogeneity: Tau² = 0.00; Chi² = 0.00, df = 5 (P = 1.00); I² = 0%						
Test for overall effect: Z = 0.00 (P = 1.00)						
**5.1.4 5 years**						
Franco et al. 2006	27	27	15	17	2.3%	1.14 [0.94, 1.38]
Wafaie et al. 2023	37	37	39	39	4.9%	1.00 [0.95, 1.05]
**Subtotal (95% CI)**		**64**		**56**	**7.2%**	**1.05 [0.87, 1.27]**
Total events	64		54			
Heterogeneity: Tau² = 0.01; Chi² = 3.84, df = 1 (P = 0.05); I² = 74%						
Test for overall effect: Z = 0.53 (P = 0.60)						
**Total (95% CI)**		**1042**		**981**	**100.0%**	**1.00 [0.96, 1.04]**
Total events	963		976			
Heterogeneity: Tau² = 0.01; Chi² = 355.53, df = 22 (P < 0.00001); I² = 94%						
Test for overall effect: Z = 0.12 (P = 0.90)						
Test for subgroup differences: Chi² = 1.74, df = 3 (P = 0.63), I² = 0%						

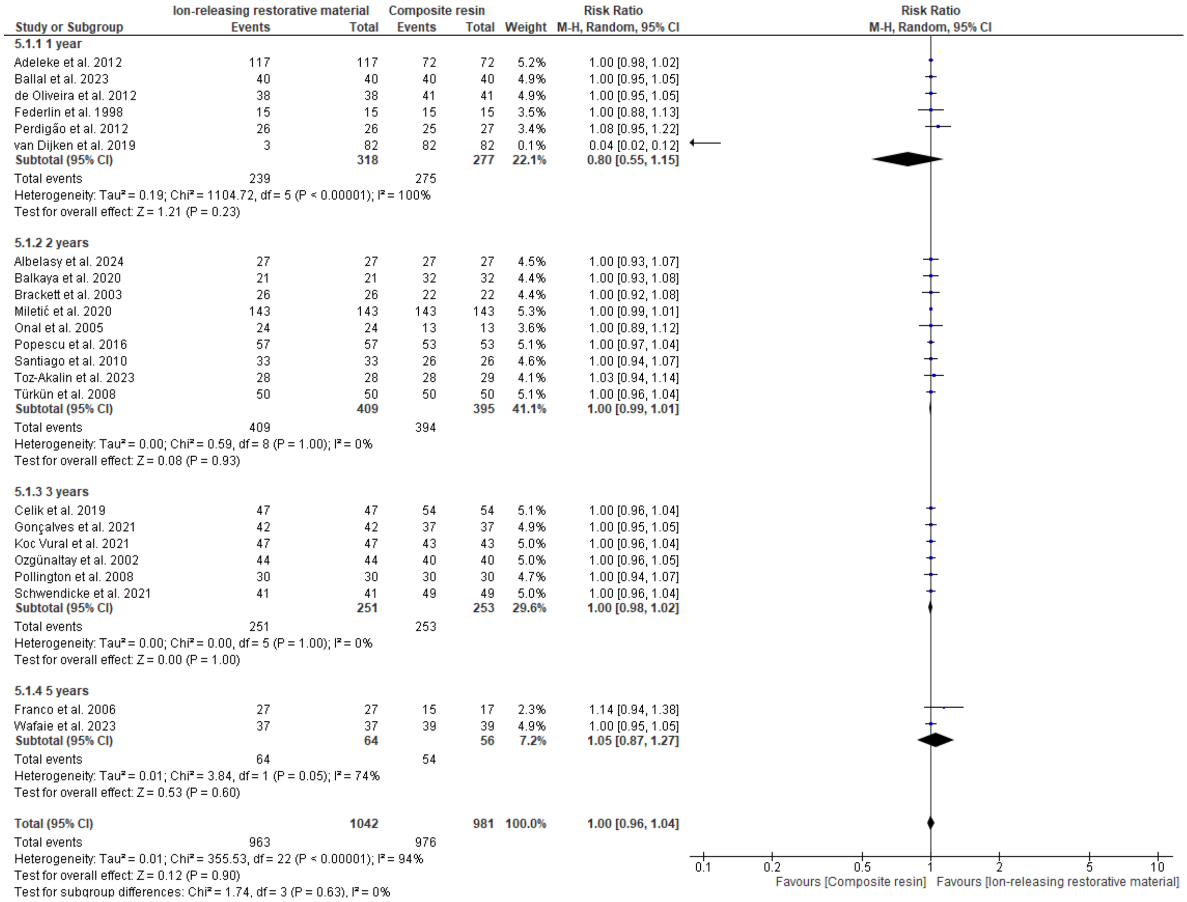

Figure S50. Absence of marginal discoloration between IRR and CR in dental restorations by follow-up time

Study or Subgroup	Ion-releasing restorative material Events	Ion-releasing restorative material Total	Composite resin Events	Composite resin Total	Weight	Risk Ratio M-H, Random, 95% CI
**5.2.1 1 year**						
Adeleke et al. 2012	130	131	105	106	11.4%	1.00 [0.98, 1.03]
Ballal et al. 2023	24	40	33	40	0.3%	0.73 [0.54, 0.97]
de Oliveira et al. 2012	38	38	41	41	6.0%	1.00 [0.95, 1.05]
Federlin et al. 1998	15	15	15	15	1.4%	1.00 [0.88, 1.13]
Hussainy et al. 2018	32	33	32	34	1.9%	1.03 [0.93, 1.14]
Perdigão et al. 2012	25	26	22	27	0.6%	1.18 [0.97, 1.43]
van Dijken et al. 2019	82	82	82	82	11.4%	1.00 [0.98, 1.02]
**Subtotal (95% CI)**		**365**		**345**	**32.9%**	**1.00 [0.98, 1.03]**
Total events	346		330			
Heterogeneity: Tau² = 0.00; Chi² = 10.47, df = 6 (P = 0.11); I² = 43%						
Test for overall effect: Z = 0.20 (P = 0.84)						
**5.2.2 2 years**						
Albelasy et al. 2024	27	27	27	27	3.6%	1.00 [0.93, 1.07]
Balkaya et al. 2020	20	21	32	32	1.4%	0.95 [0.84, 1.07]
Brackett et al. 2003	26	26	22	22	2.9%	1.00 [0.92, 1.08]
Miletić et al. 2020	142	142	142	142	14.0%	1.00 [0.99, 1.01]
Onal et al. 2005	24	24	13	13	1.6%	1.00 [0.89, 1.12]
Popescu et al. 2016	32	57	31	53	0.2%	0.96 [0.70, 1.33]
Santiago et al. 2010	33	33	26	26	4.0%	1.00 [0.94, 1.07]
Türkün et al. 2008	50	50	50	50	7.8%	1.00 [0.96, 1.04]
**Subtotal (95% CI)**		**380**		**365**	**35.5%**	**1.00 [0.99, 1.01]**
Total events	354		343			
Heterogeneity: Tau² = 0.00; Chi² = 2.16, df = 7 (P = 0.95); I² = 0%						
Test for overall effect: Z = 0.10 (P = 0.92)						
**5.2.3 3 years**						
Celik et al. 2019	47	47	54	54	7.8%	1.00 [0.96, 1.04]
Folwaczny et al. 2001	19	23	20	22	0.4%	0.91 [0.72, 1.14]
Gonçalves et al. 2021	29	42	25	37	0.3%	1.02 [0.76, 1.38]
Koc Vural et al. 2021	31	47	40	43	0.5%	0.71 [0.57, 0.88]
Ozgünaltay et al. 2002	44	44	40	40	6.5%	1.00 [0.96, 1.05]
Pollington et al. 2008	28	30	28	30	1.2%	1.00 [0.87, 1.14]
Schwendicke et al. 2021	41	41	49	49	6.9%	1.00 [0.96, 1.04]
**Subtotal (95% CI)**		**274**		**275**	**23.5%**	**0.97 [0.91, 1.04]**
Total events	239		256			
Heterogeneity: Tau² = 0.00; Chi² = 26.47, df = 6 (P = 0.0002); I² = 77%						
Test for overall effect: Z = 0.83 (P = 0.41)						
**5.2.4 5 years**						
Franco et al. 2006	27	27	17	17	2.3%	1.00 [0.91, 1.10]
Wafaie et al. 2023	37	37	39	39	5.7%	1.00 [0.95, 1.05]
**Subtotal (95% CI)**		**64**		**56**	**8.0%**	**1.00 [0.96, 1.05]**
Total events	64		56			
Heterogeneity: Tau² = 0.00; Chi² = 0.00, df = 1 (P = 1.00); I² = 0%						
Test for overall effect: Z = 0.00 (P = 1.00)						
**Total (95% CI)**		**1083**		**1041**	**100.0%**	**1.00 [0.98, 1.01]**
Total events	1003		985			
Heterogeneity: Tau² = 0.00; Chi² = 38.17, df = 23 (P = 0.02); I² = 40%						
Test for overall effect: Z = 0.25 (P = 0.80)						
Test for subgroup differences: Chi² = 0.72, df = 3 (P = 0.87), I² = 0%						

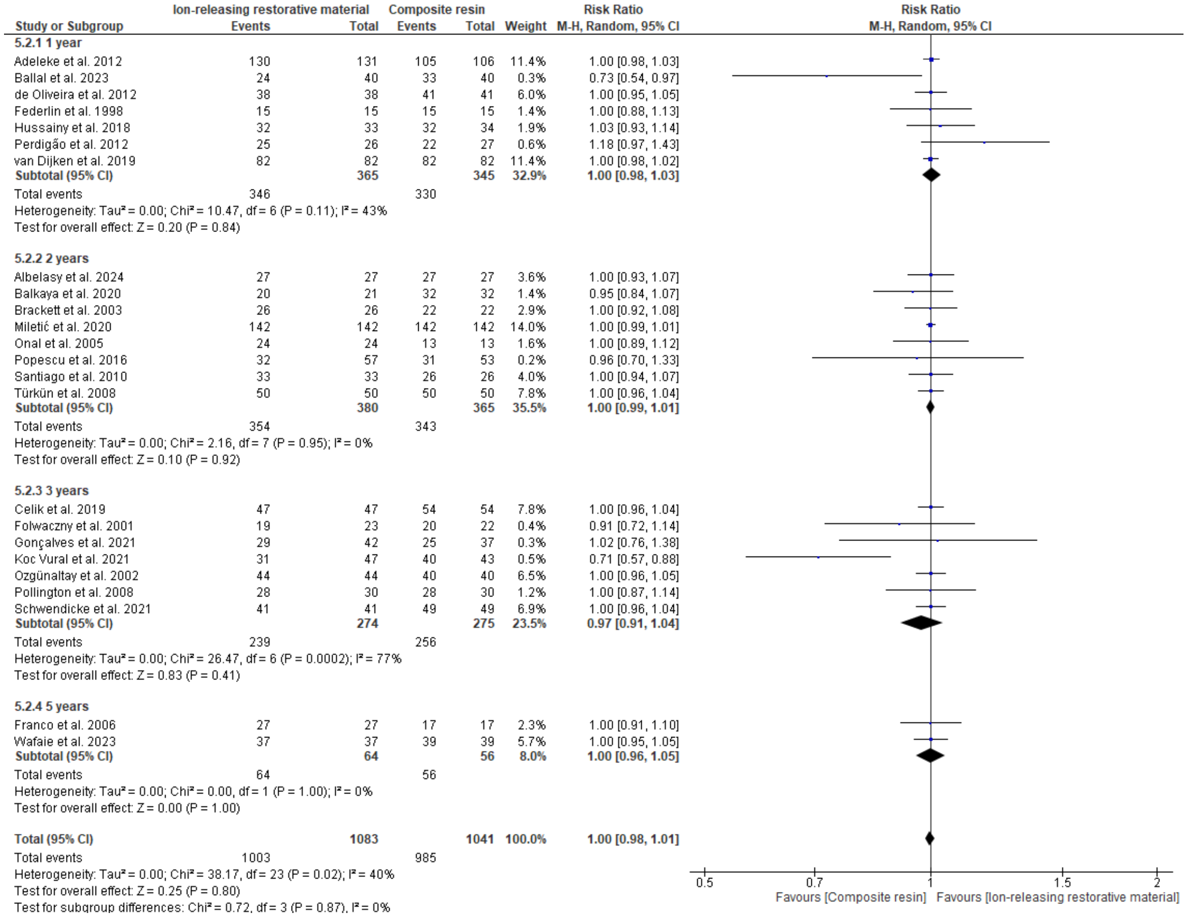

Figure S51. Adequate of marginal adaptation between IRR and CR in dental restorations by follow-up time

Study or Subgroup	Ion-releasing restorative material Events	Total	Composite resin Events	Total	Weight	Risk Ratio M-H, Random, 95% CI
**5.3.1 1 year**						
Adeleke et al. 2012	131	131	105	106	14.7%	1.01 [0.99, 1.04]
de Oliveira et al. 2012	38	38	41	41	3.8%	1.00 [0.95, 1.05]
Hussainy et al. 2018	32	33	32	34	0.9%	1.03 [0.93, 1.14]
Perdigão et al. 2012	26	26	23	27	0.3%	1.17 [0.99, 1.39]
Saghir et al. 2023	23	30	21	30	0.1%	1.10 [0.81, 1.49]
van Dijken et al. 2019	74	82	81	82	1.6%	0.91 [0.85, 0.98]
**Subtotal (95% CI)**		**340**		**320**	**21.5%**	**1.00 [0.96, 1.04]**
Total events	324		303			
Heterogeneity: Tau² = 0.00; Chi² = 9.98, df = 5 (P = 0.08); I² = 50%						
Test for overall effect: Z = 0.05 (P = 0.96)						
**5.3.2 2 years**						
Albelasy et al. 2024	27	27	27	27	1.8%	1.00 [0.93, 1.07]
Balkaya et al. 2020	20	21	32	32	0.6%	0.95 [0.84, 1.07]
Brackett et al. 2003	26	26	22	22	1.4%	1.00 [0.92, 1.08]
Miletić et al. 2020	142	142	142	142	48.6%	1.00 [0.99, 1.01]
Popescu et al. 2016	32	57	30	53	0.1%	0.99 [0.71, 1.38]
Toz-Akalin et al. 2023	28	28	29	29	2.0%	1.00 [0.94, 1.07]
Türkün et al. 2008	50	50	50	50	6.1%	1.00 [0.96, 1.04]
**Subtotal (95% CI)**		**351**		**355**	**60.7%**	**1.00 [0.99, 1.01]**
Total events	325		332			
Heterogeneity: Tau² = 0.00; Chi² = 1.30, df = 6 (P = 0.97); I² = 0%						
Test for overall effect: Z = 0.09 (P = 0.93)						
**5.3.3 3 years**						
Celik et al. 2019	47	47	54	54	6.2%	1.00 [0.96, 1.04]
Koc Vural et al. 2021	47	47	43	43	5.0%	1.00 [0.96, 1.04]
Ozgünaltay et al. 2002	44	44	40	40	4.3%	1.00 [0.96, 1.05]
Pollington et al. 2008	24	30	25	30	0.2%	0.96 [0.76, 1.22]
Schwendicke et al. 2021	40	41	49	49	2.2%	0.97 [0.91, 1.04]
**Subtotal (95% CI)**		**209**		**216**	**17.8%**	**1.00 [0.97, 1.02]**
Total events	202		211			
Heterogeneity: Tau² = 0.00; Chi² = 1.00, df = 4 (P = 0.91); I² = 0%						
Test for overall effect: Z = 0.31 (P = 0.75)						
**Total (95% CI)**		**900**		**891**	**100.0%**	**1.00 [0.99, 1.01]**
Total events	851		846			
Heterogeneity: Tau² = 0.00; Chi² = 12.10, df = 17 (P = 0.79); I² = 0%						
Test for overall effect: Z = 0.02 (P = 0.98)						
Test for subgroup differences: Chi² = 0.06, df = 2 (P = 0.97), I² = 0%						

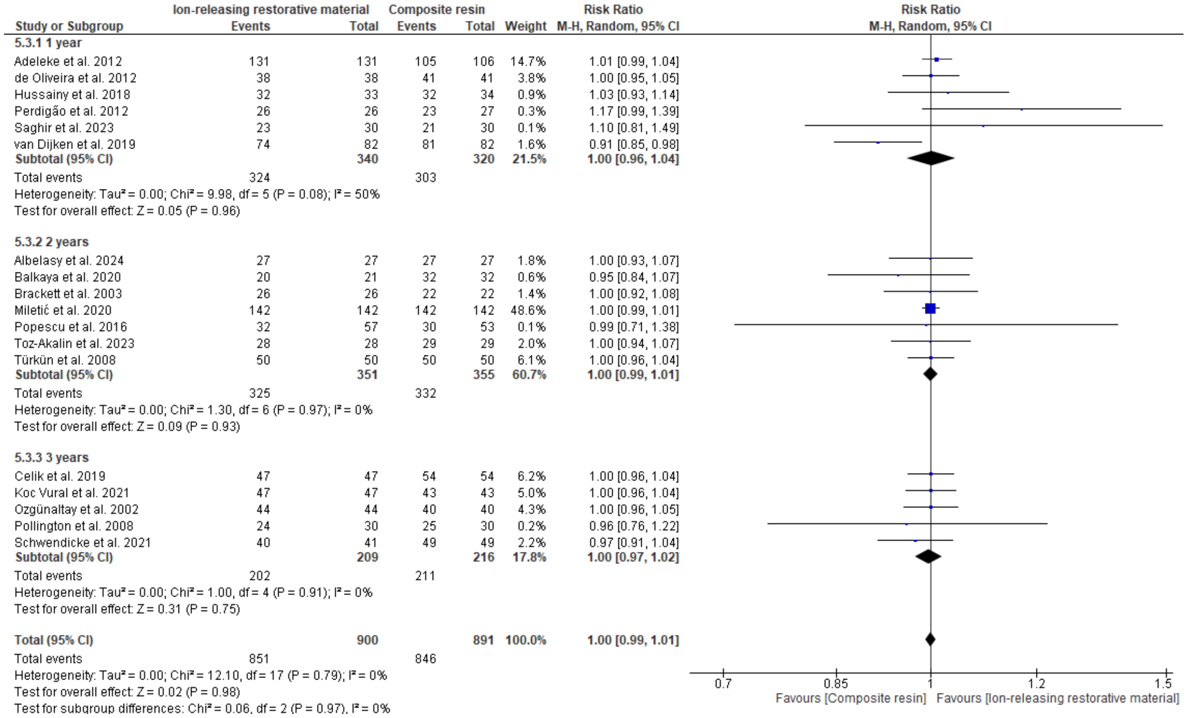

Figure S52. Adequate marginal or tooth integrity between IRR and CR in dental restorations by follow-up time

Study or Subgroup	Ion-releasing restorative material Events	Total	Composite resin Events	Total	Weight	Risk Ratio M-H, Random, 95% CI
**5.4.1 1 year**						
Ballal et al. 2023	23	40	31	40	0.6%	0.74 [0.54, 1.02]
Federlin et al. 1998	15	15	13	15	1.1%	1.15 [0.91, 1.44]
**Subtotal (95% CI)**		**55**		**55**	**1.6%**	**0.93 [0.56, 1.55]**
Total events	38		44			
Heterogeneity: Tau² = 0.11; Chi² = 6.81, df = 1 (P = 0.009); I² = 85%						
Test for overall effect: Z = 0.27 (P = 0.79)						
**5.4.2 2 years**						
Albelasy et al. 2024	26	27	27	27	4.9%	0.96 [0.87, 1.07]
Miletić et al. 2020	143	143	143	143	34.4%	1.00 [0.99, 1.01]
Onal et al. 2005	24	24	13	13	3.9%	1.00 [0.89, 1.12]
Santiago et al. 2010	33	33	26	26	9.8%	1.00 [0.94, 1.07]
Toz-Akalin et al. 2023	28	28	29	29	9.6%	1.00 [0.94, 1.07]
**Subtotal (95% CI)**		**255**		**238**	**62.5%**	**1.00 [0.99, 1.01]**
Total events	254		238			
Heterogeneity: Tau² = 0.00; Chi² = 0.78, df = 4 (P = 0.94); I² = 0%						
Test for overall effect: Z = 0.09 (P = 0.93)						
**5.4.3 3 years**						
Celik et al. 2019	47	47	54	54	19.3%	1.00 [0.96, 1.04]
Folwaczny et al. 2001	17	23	20	22	0.7%	0.81 [0.62, 1.07]
Gonçalves et al. 2021	30	42	25	37	0.6%	1.06 [0.79, 1.42]
**Subtotal (95% CI)**		**112**		**113**	**20.7%**	**0.97 [0.84, 1.12]**
Total events	94		99			
Heterogeneity: Tau² = 0.01; Chi² = 3.44, df = 2 (P = 0.18); I² = 42%						
Test for overall effect: Z = 0.41 (P = 0.68)						
**5.4.4 5 years**						
Franco et al. 2006	23	27	13	17	0.6%	1.11 [0.82, 1.51]
Wafaie et al. 2023	39	39	39	39	14.6%	1.00 [0.95, 1.05]
**Subtotal (95% CI)**		**66**		**56**	**15.2%**	**1.03 [0.85, 1.23]**
Total events	62		52			
Heterogeneity: Tau² = 0.01; Chi² = 1.88, df = 1 (P = 0.17); I² = 47%						
Test for overall effect: Z = 0.28 (P = 0.78)						
**Total (95% CI)**		**488**		**462**	**100.0%**	**1.00 [0.97, 1.02]**
Total events	448		433			
Heterogeneity: Tau² = 0.00; Chi² = 15.43, df = 11 (P = 0.16); I² = 29%						
Test for overall effect: Z = 0.21 (P = 0.83)						
Test for subgroup differences: Chi² = 0.32, df = 3 (P = 0.96), I² = 0%						

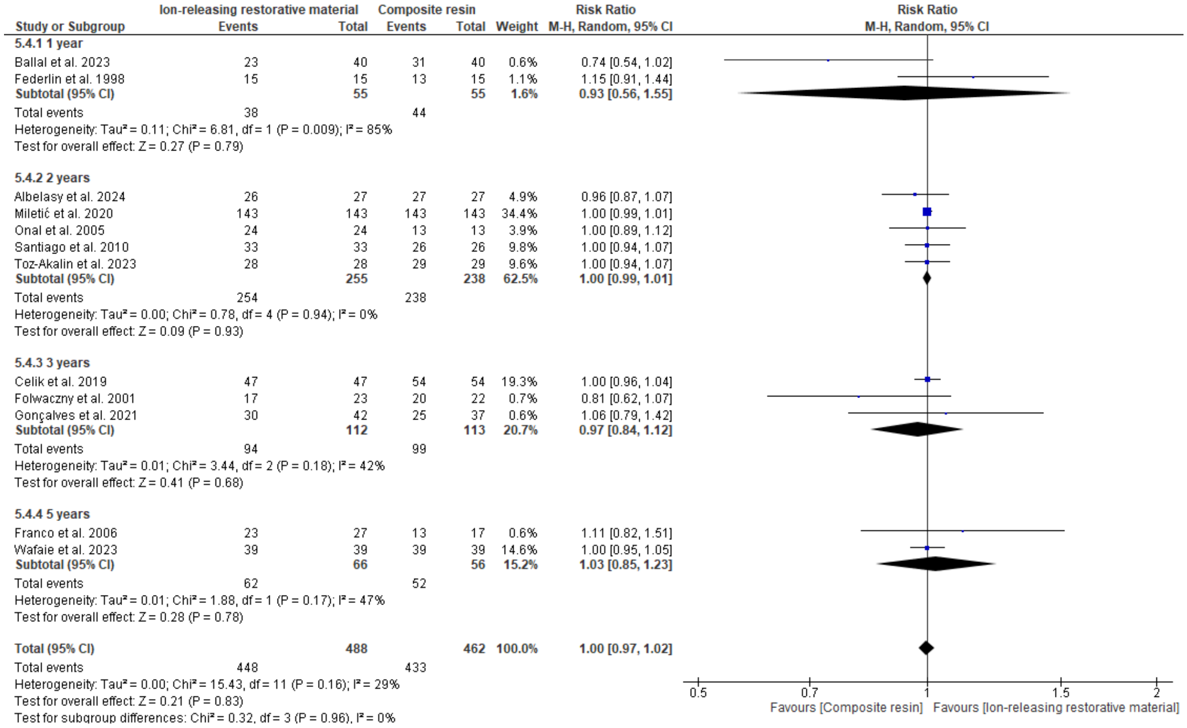

Figure S53. Adequate color or translucency between IRR and CR in dental restorations by follow-up time

Study or Subgroup	Ion-releasing restorative material Events	Total	Composite resin Events	Total	Weight	Risk Ratio M-H, Random, 95% CI
**5.5.1 1 year**						
de Oliveira et al. 2012	38	38	41	41	11.5%	1.00 [0.95, 1.05]
Federlin et al. 1998	15	15	15	15	2.8%	1.00 [0.88, 1.13]
Hussainy et al. 2018	32	33	32	34	3.9%	1.03 [0.93, 1.14]
Perdigão et al. 2012	26	26	22	27	1.3%	1.22 [1.01, 1.48]
van Dijken et al. 2019	77	82	82	82	9.0%	0.94 [0.88, 1.00]
**Subtotal (95% CI)**		**194**		**199**	**28.5%**	**1.00 [0.95, 1.06]**
Total events	188		192			
Heterogeneity: Tau² = 0.00; Chi² = 9.15, df = 4 (P = 0.06); I² = 56%						
Test for overall effect: Z = 0.11 (P = 0.91)						
**5.5.2 2 years**						
Balkaya et al. 2020	15	21	32	32	0.6%	0.72 [0.54, 0.94]
Brackett et al. 2003	26	26	22	22	5.9%	1.00 [0.92, 1.08]
Onal et al. 2005	24	24	13	13	3.2%	1.00 [0.89, 1.12]
Popescu et al. 2016	50	57	44	53	1.9%	1.06 [0.90, 1.23]
Toz-Akalin et al. 2023	28	28	29	29	7.7%	1.00 [0.94, 1.07]
Türkün et al. 2008	50	50	47	50	6.0%	1.06 [0.98, 1.15]
**Subtotal (95% CI)**		**206**		**199**	**25.3%**	**1.01 [0.95, 1.07]**
Total events	193		187			
Heterogeneity: Tau² = 0.00; Chi² = 9.31, df = 5 (P = 0.10); I² = 46%						
Test for overall effect: Z = 0.21 (P = 0.84)						
**5.5.3 3 years**						
Celik et al. 2019	47	47	54	54	14.7%	1.00 [0.96, 1.04]
Folwaczny et al. 2001	23	23	22	22	5.4%	1.00 [0.92, 1.09]
Gonçalves et al. 2021	27	42	29	37	0.6%	0.82 [0.62, 1.09]
Ozgünaltay et al. 2002	44	44	40	40	12.3%	1.00 [0.96, 1.05]
Schwendicke et al. 2021	41	41	49	49	13.1%	1.00 [0.96, 1.04]
**Subtotal (95% CI)**		**197**		**202**	**46.1%**	**1.00 [0.96, 1.03]**
Total events	182		194			
Heterogeneity: Tau² = 0.00; Chi² = 7.37, df = 4 (P = 0.12); I² = 46%						
Test for overall effect: Z = 0.17 (P = 0.87)						
**Total (95% CI)**		**597**		**600**	**100.0%**	**1.00 [0.98, 1.02]**
Total events	563		573			
Heterogeneity: Tau² = 0.00; Chi² = 20.52, df = 15 (P = 0.15); I² = 27%						
Test for overall effect: Z = 0.05 (P = 0.96)						
Test for subgroup differences: Chi² = 0.08, df = 2 (P = 0.96), I² = 0%						

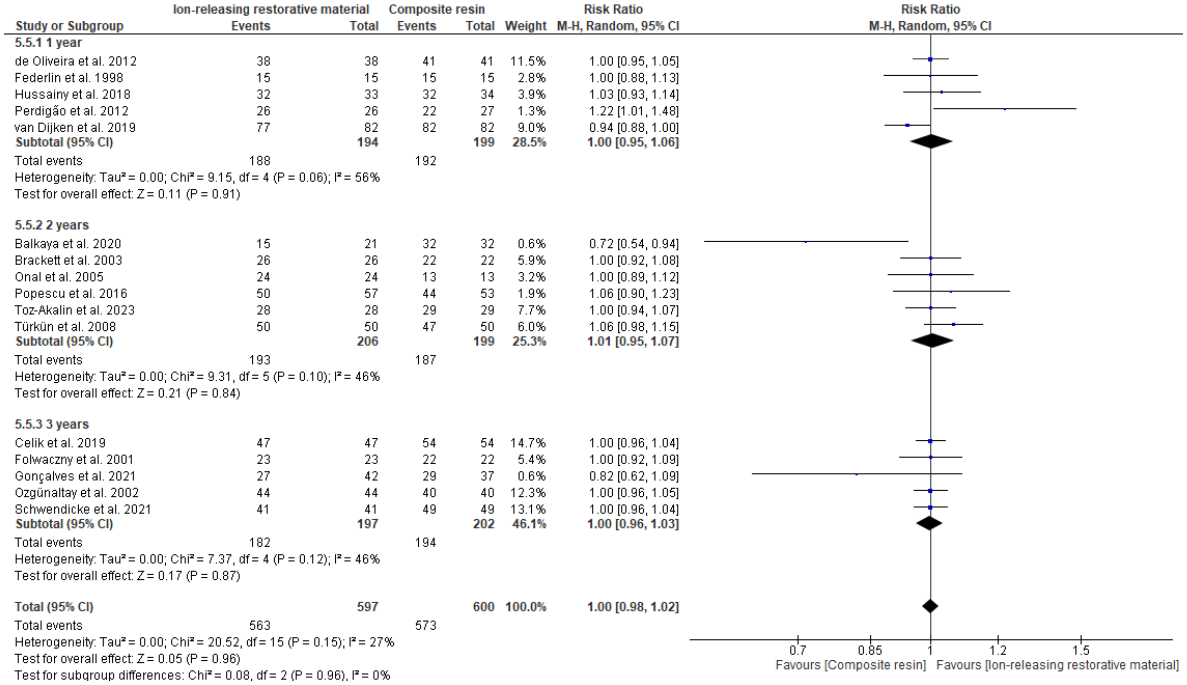

Figure S54. Proper surface texture or luster between IRR and CR in dental restorations by follow-up time

Study or Subgroup	Ion-releasing restorative material Events	Total	Composite resin Events	Total	Weight	Risk Ratio M-H, Random, 95% CI
**5.6.1 1 year**						
Federlin et al. 1998	15	15	15	15	4.4%	1.00 [0.88, 1.13]
Hussainy et al. 2018	32	33	32	34	5.7%	1.03 [0.93, 1.14]
Perdigão et al. 2012	11	26	25	27	0.4%	0.46 [0.29, 0.72]
Saghir et al. 2023	25	30	18	30	0.8%	1.39 [1.00, 1.94]
van Dijken et al. 2019	82	82	82	82	15.1%	1.00 [0.98, 1.02]
**Subtotal (95% CI)**		**186**		**188**	**26.4%**	**0.99 [0.86, 1.14]**
Total events	165		172			
Heterogeneity: Tau² = 0.02; Chi² = 23.17, df = 4 (P = 0.0001); I² = 83%						
Test for overall effect: Z = 0.14 (P = 0.89)						
**5.6.2 2 years**						
Balkaya et al. 2020	19	21	32	32	3.1%	0.90 [0.77, 1.05]
Brackett et al. 2003	26	26	22	22	7.7%	1.00 [0.92, 1.08]
Onal et al. 2005	24	24	13	13	4.9%	1.00 [0.89, 1.12]
Toz-Akalin et al. 2023	28	28	29	29	9.2%	1.00 [0.94, 1.07]
Türkün et al. 2008	50	50	50	50	13.1%	1.00 [0.96, 1.04]
**Subtotal (95% CI)**		**149**		**146**	**38.0%**	**1.00 [0.97, 1.03]**
Total events	147		146			
Heterogeneity: Tau² = 0.00; Chi² = 2.59, df = 4 (P = 0.63); I² = 0%						
Test for overall effect: Z = 0.25 (P = 0.80)						
**5.6.3 3 years**						
Celik et al. 2019	47	47	54	54	13.1%	1.00 [0.96, 1.04]
Folwaczny et al. 2001	23	23	22	22	7.3%	1.00 [0.92, 1.09]
Gonçalves et al. 2021	34	42	35	37	2.8%	0.86 [0.73, 1.01]
Schwendicke et al. 2021	41	41	49	49	12.4%	1.00 [0.96, 1.04]
**Subtotal (95% CI)**		**153**		**162**	**35.6%**	**0.99 [0.94, 1.04]**
Total events	145		160			
Heterogeneity: Tau² = 0.00; Chi² = 8.37, df = 3 (P = 0.04); I² = 64%						
Test for overall effect: Z = 0.48 (P = 0.63)						
**Total (95% CI)**		**488**		**496**	**100.0%**	**0.99 [0.96, 1.02]**
Total events	457		478			
Heterogeneity: Tau² = 0.00; Chi² = 32.55, df = 13 (P = 0.002); I² = 60%						
Test for overall effect: Z = 0.43 (P = 0.67)						
Test for subgroup differences: Chi² = 0.09, df = 2 (P = 0.95), I² = 0%						

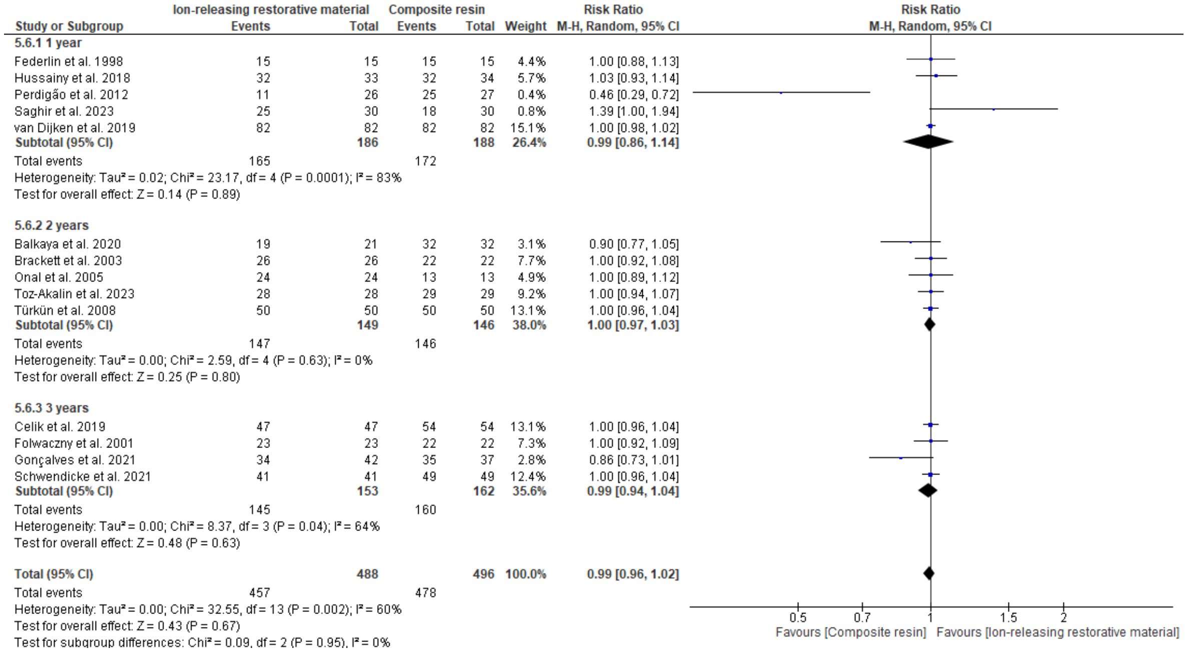

Figure S55. Proper surface staining between IRR and CR in dental restorations by follow-up time

Study or Subgroup	Ion-releasing restorative material Events	Total	Composite resin Events	Total	Weight	Risk Ratio M-H, Random, 95% CI
**5.7.1 2 years**						
Miletić et al. 2020	143	143	143	143	77.9%	1.00 [0.99, 1.01]
Toz-Akalin et al. 2023	28	28	29	29	3.2%	1.00 [0.94, 1.07]
**Subtotal (95% CI)**		**171**		**172**	**81.1%**	**1.00 [0.99, 1.01]**
Total events	171		172			
Heterogeneity: Tau² = 0.00; Chi² = 0.00, df = 1 (P = 1.00); I² = 0%						
Test for overall effect: Z = 0.00 (P = 1.00)						
**5.7.2 3 years**						
Celik et al. 2019	47	47	54	54	9.8%	1.00 [0.96, 1.04]
Gonçalves et al. 2021	40	42	35	37	1.4%	1.01 [0.91, 1.12]
Schwendicke et al. 2021	41	41	49	49	7.7%	1.00 [0.96, 1.04]
**Subtotal (95% CI)**		**130**		**140**	**18.9%**	**1.00 [0.97, 1.03]**
Total events	128		138			
Heterogeneity: Tau² = 0.00; Chi² = 0.03, df = 2 (P = 0.99); I² = 0%						
Test for overall effect: Z = 0.04 (P = 0.97)						
**Total (95% CI)**		**301**		**312**	**100.0%**	**1.00 [0.99, 1.01]**
Total events	299		310			
Heterogeneity: Tau² = 0.00; Chi² = 0.03, df = 4 (P = 1.00); I² = 0%						
Test for overall effect: Z = 0.02 (P = 0.99)						
Test for subgroup differences: Chi² = 0.00, df = 1 (P = 0.97), I² = 0%						

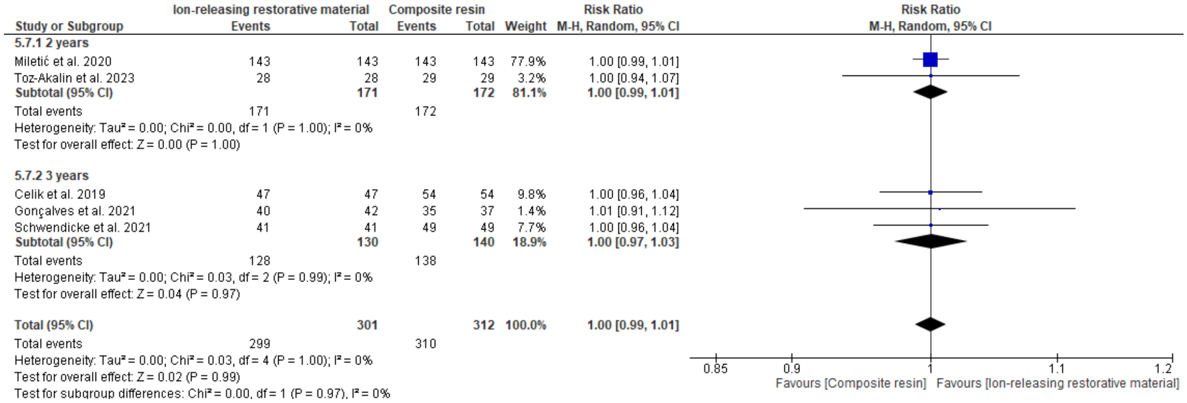

Figure S56. Retention between IRR and CR in dental restorations by follow-up time

Study or Subgroup	Ion-releasing restorative material Events	Total	Composite resin Events	Total	Weight	Risk Ratio M-H, Random, 95% CI
**5.8.1 1 year**						
Adeleke et al. 2012	131	144	106	143	4.6%	1.23 [1.10, 1.37]
Ballal et al. 2023	24	40	33	40	1.4%	0.73 [0.54, 0.97]
de Oliveira et al. 2012	38	40	41	41	5.3%	0.95 [0.87, 1.03]
Hussainy et al. 2018	32	33	32	34	4.7%	1.03 [0.93, 1.14]
Perdigão et al. 2012	26	26	25	27	4.0%	1.08 [0.95, 1.22]
Saghir et al. 2023	28	30	19	30	1.4%	1.47 [1.10, 1.97]
van Dijken et al. 2019	62	82	80	82	4.0%	0.78 [0.68, 0.88]
**Subtotal (95% CI)**		**395**		**397**	**25.6%**	**1.01 [0.88, 1.15]**
Total events	341		336			
Heterogeneity: Tau² = 0.03; Chi² = 43.67, df = 6 (P < 0.00001); I² = 86%						
Test for overall effect: Z = 0.12 (P = 0.90)						
**5.8.2 2 years**						
Albelasy et al. 2024	26	27	27	27	4.8%	0.96 [0.87, 1.07]
Balkaya et al. 2020	15	21	32	32	1.6%	0.72 [0.54, 0.94]
Brackett et al. 2003	26	27	22	27	2.6%	1.18 [0.97, 1.44]
Miletić et al. 2020	143	143	141	143	7.0%	1.01 [0.99, 1.04]
Onal et al. 2005	24	24	13	18	1.4%	1.38 [1.03, 1.85]
Popescu et al. 2016	54	57	53	57	5.0%	1.02 [0.93, 1.12]
Santiago et al. 2010	33	33	26	33	2.7%	1.26 [1.05, 1.52]
Toz-Akalin et al. 2023	28	29	29	29	5.0%	0.97 [0.88, 1.06]
Türkün et al. 2008	48	50	50	50	5.9%	0.96 [0.90, 1.03]
**Subtotal (95% CI)**		**411**		**416**	**36.0%**	**1.01 [0.96, 1.08]**
Total events	397		393			
Heterogeneity: Tau² = 0.00; Chi² = 24.02, df = 8 (P = 0.002); I² = 67%						
Test for overall effect: Z = 0.48 (P = 0.63)						
**5.8.3 3 years**						
Celik et al. 2019	47	49	54	54	5.9%	0.96 [0.90, 1.03]
Folwaczny et al. 2001	23	26	22	23	3.1%	0.92 [0.79, 1.09]
Gonçalves et al. 2021	42	43	37	42	4.2%	1.11 [0.98, 1.25]
Koc Vural et al. 2021	47	51	43	51	3.6%	1.09 [0.95, 1.26]
Ozgünaltay et al. 2002	44	45	40	42	5.5%	1.03 [0.95, 1.11]
Pollington et al. 2008	26	30	26	30	2.5%	1.00 [0.82, 1.22]
Schwendicke et al. 2021	39	41	49	49	5.5%	0.95 [0.88, 1.03]
**Subtotal (95% CI)**		**285**		**291**	**30.3%**	**1.00 [0.95, 1.05]**
Total events	268		271			
Heterogeneity: Tau² = 0.00; Chi² = 9.29, df = 6 (P = 0.16); I² = 35%						
Test for overall effect: Z = 0.01 (P = 0.99)						
**5.8.4 5 years**						
Franco et al. 2006	27	28	27	33	2.9%	1.18 [0.99, 1.41]
Wafaie et al. 2023	37	39	39	39	5.3%	0.95 [0.87, 1.04]
**Subtotal (95% CI)**		**67**		**72**	**8.1%**	**1.05 [0.82, 1.33]**
Total events	64		66			
Heterogeneity: Tau² = 0.03; Chi² = 6.00, df = 1 (P = 0.01); I² = 83%						
Test for overall effect: Z = 0.37 (P = 0.71)						
**Total (95% CI)**		**1158**		**1176**	**100.0%**	**1.01 [0.97, 1.05]**
Total events	1070		1066			
Heterogeneity: Tau² = 0.01; Chi² = 82.69, df = 24 (P < 0.00001); I² = 71%						
Test for overall effect: Z = 0.57 (P = 0.57)						
Test for subgroup differences: Chi² = 0.23, df = 3 (P = 0.97), I² = 0%						

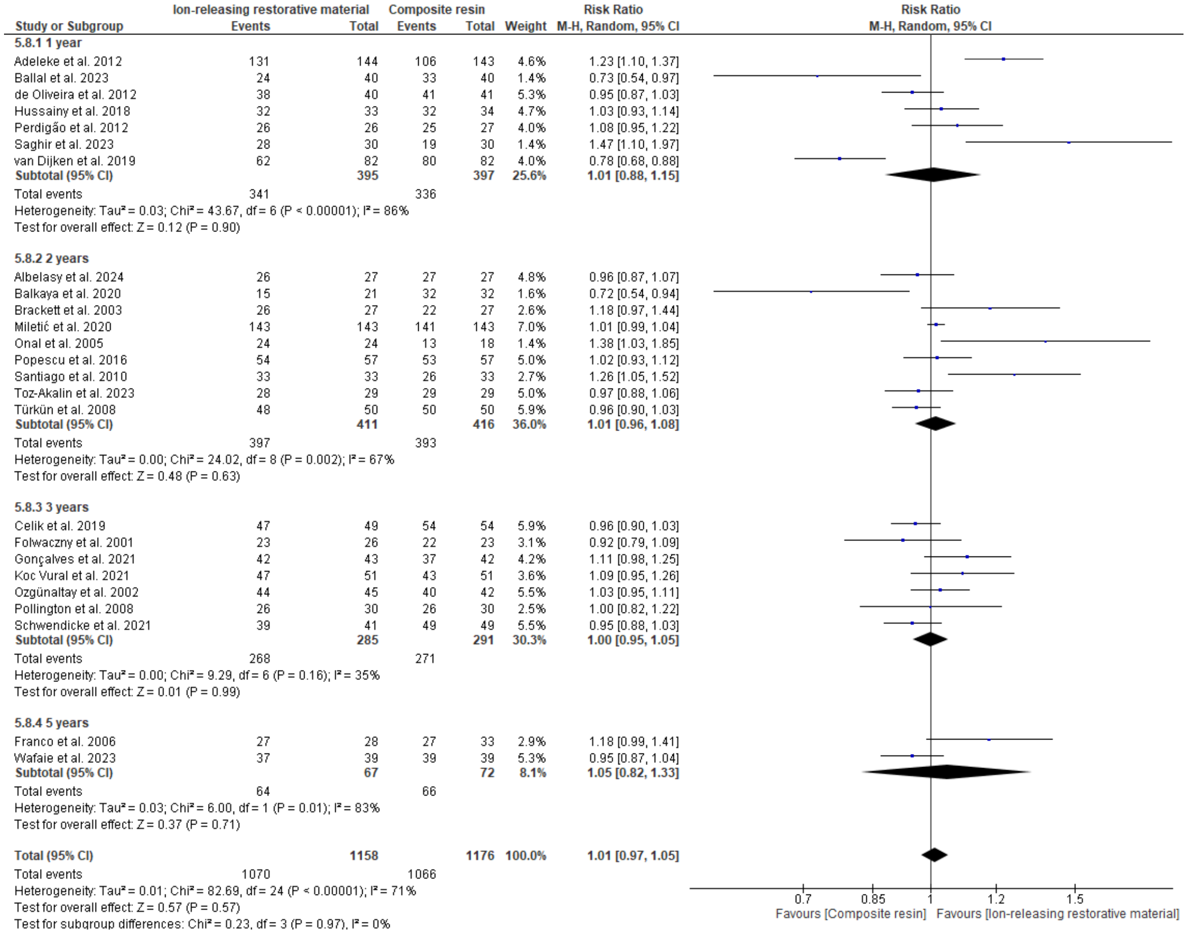

Figure S57. Absence of wear between IRR and CR in dental restorations by follow-up time

Study or Subgroup	Ion-releasing restorative material Events	Ion-releasing restorative material Total	Composite resin Events	Composite resin Total	Weight	Risk Ratio M-H, Random, 95% CI
**5.9.1 1 year**						
Adeleke et al. 2012	131	131	106	106	33.9%	1.00 [0.98, 1.02]
Perdigão et al. 2012	26	26	25	27	0.6%	1.08 [0.95, 1.22]
**Subtotal (95% CI)**		**157**		**133**	**34.5%**	**1.03 [0.92, 1.15]**
Total events	157		131			
Heterogeneity: Tau² = 0.01; Chi² = 3.42, df = 1 (P = 0.06); I² = 71%						
Test for overall effect: Z = 0.46 (P = 0.64)						
**5.9.2 2 years**						
Miletić et al. 2020	143	143	143	143	51.0%	1.00 [0.99, 1.01]
Toz-Akalin et al. 2023	28	28	29	29	2.1%	1.00 [0.94, 1.07]
**Subtotal (95% CI)**		**171**		**172**	**53.1%**	**1.00 [0.99, 1.01]**
Total events	171		172			
Heterogeneity: Tau² = 0.00; Chi² = 0.00, df = 1 (P = 1.00); I² = 0%						
Test for overall effect: Z = 0.00 (P = 1.00)						
**5.9.3 3 years**						
Celik et al. 2019	47	47	54	54	6.4%	1.00 [0.96, 1.04]
Gonçalves et al. 2021	39	42	36	37	1.0%	0.95 [0.86, 1.05]
Schwendicke et al. 2021	41	41	49	49	5.0%	1.00 [0.96, 1.04]
**Subtotal (95% CI)**		**130**		**140**	**12.4%**	**1.00 [0.97, 1.02]**
Total events	127		139			
Heterogeneity: Tau² = 0.00; Chi² = 1.23, df = 2 (P = 0.54); I² = 0%						
Test for overall effect: Z = 0.26 (P = 0.80)						
**Total (95% CI)**		**458**		**445**	**100.0%**	**1.00 [0.99, 1.01]**
Total events	455		442			
Heterogeneity: Tau² = 0.00; Chi² = 2.19, df = 6 (P = 0.90); I² = 0%						
Test for overall effect: Z = 0.00 (P = 1.00)						
Test for subgroup differences: Chi² = 0.28, df = 2 (P = 0.87), I² = 0%						

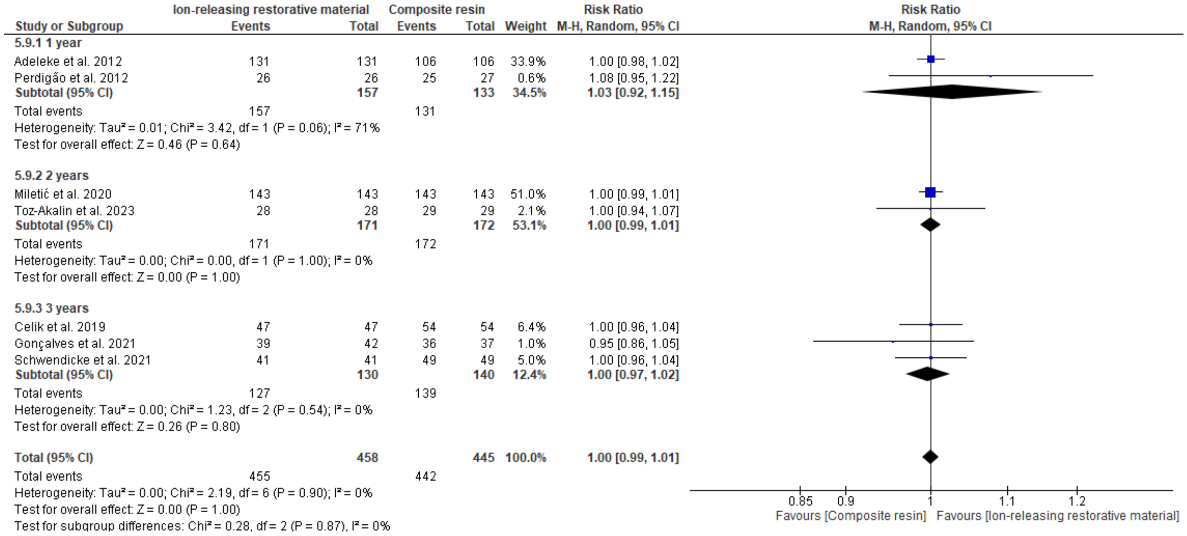

Figure S58. Proper anatomic form between IRR and CR in dental restorations by follow-up time

Study or Subgroup	Ion-releasing restorative material Events	Ion-releasing restorative material Total	Composite resin Events	Composite resin Total	Weight	Risk Ratio M-H, Random, 95% CI
**5.10.1 1 year**						
Ballal et al. 2023	22	40	32	40	0.9%	0.69 [0.50, 0.95]
de Oliveira et al. 2012	38	38	41	41	8.0%	1.00 [0.95, 1.05]
Federlin et al. 1998	15	15	15	15	3.9%	1.00 [0.88, 1.13]
van Dijken et al. 2019	74	82	81	82	6.4%	0.91 [0.85, 0.98]
**Subtotal (95% CI)**		**175**		**178**	**19.2%**	**0.93 [0.81, 1.07]**
Total events	149		169			
Heterogeneity: Tau² = 0.02; Chi² = 23.93, df = 3 (P < 0.0001); I² = 87%						
Test for overall effect: Z = 1.02 (P = 0.31)						
**5.10.2 2 years**						
Balkaya et al. 2020	20	21	32	32	4.1%	0.95 [0.84, 1.07]
Brackett et al. 2003	26	26	22	22	6.1%	1.00 [0.92, 1.08]
Miletić et al. 2020	143	143	143	143	9.7%	1.00 [0.99, 1.01]
Onal et al. 2005	24	24	13	13	4.3%	1.00 [0.89, 1.12]
Popescu et al. 2016	57	57	53	53	8.8%	1.00 [0.97, 1.04]
Santiago et al. 2010	33	33	25	26	5.0%	1.04 [0.94, 1.15]
**Subtotal (95% CI)**		**304**		**289**	**38.0%**	**1.00 [0.99, 1.01]**
Total events	303		288			
Heterogeneity: Tau² = 0.00; Chi² = 1.50, df = 5 (P = 0.91); I² = 0%						
Test for overall effect: Z = 0.01 (P = 0.99)						
**5.10.3 3 years**						
Celik et al. 2019	45	47	54	54	6.6%	0.96 [0.89, 1.03]
Folwaczny et al. 2001	14	23	22	22	0.8%	0.62 [0.44, 0.86]
Gonçalves et al. 2021	40	42	36	37	5.7%	0.98 [0.90, 1.07]
Ozgünaltay et al. 2002	44	44	40	40	8.2%	1.00 [0.96, 1.05]
Pollington et al. 2008	27	30	29	30	3.5%	0.93 [0.81, 1.07]
Schwendicke et al. 2021	41	41	49	49	8.4%	1.00 [0.96, 1.04]
**Subtotal (95% CI)**		**227**		**232**	**33.3%**	**0.96 [0.90, 1.03]**
Total events	211		230			
Heterogeneity: Tau² = 0.00; Chi² = 22.32, df = 5 (P = 0.0005); I² = 78%						
Test for overall effect: Z = 1.13 (P = 0.26)						
**5.10.4 5 years**						
Franco et al. 2006	23	27	15	17	1.6%	0.97 [0.76, 1.22]
Wafaie et al. 2023	37	37	39	39	7.9%	1.00 [0.95, 1.05]
**Subtotal (95% CI)**		**64**		**56**	**9.5%**	**1.00 [0.95, 1.05]**
Total events	60		54			
Heterogeneity: Tau² = 0.00; Chi² = 0.23, df = 1 (P = 0.63); I² = 0%						
Test for overall effect: Z = 0.06 (P = 0.95)						
**Total (95% CI)**		**770**		**755**	**100.0%**	**0.98 [0.95, 1.01]**
Total events	723		741			
Heterogeneity: Tau² = 0.00; Chi² = 74.95, df = 17 (P < 0.00001); I² = 77%						
Test for overall effect: Z = 1.27 (P = 0.20)						
Test for subgroup differences: Chi² = 2.24, df = 3 (P = 0.52), I² = 0%						

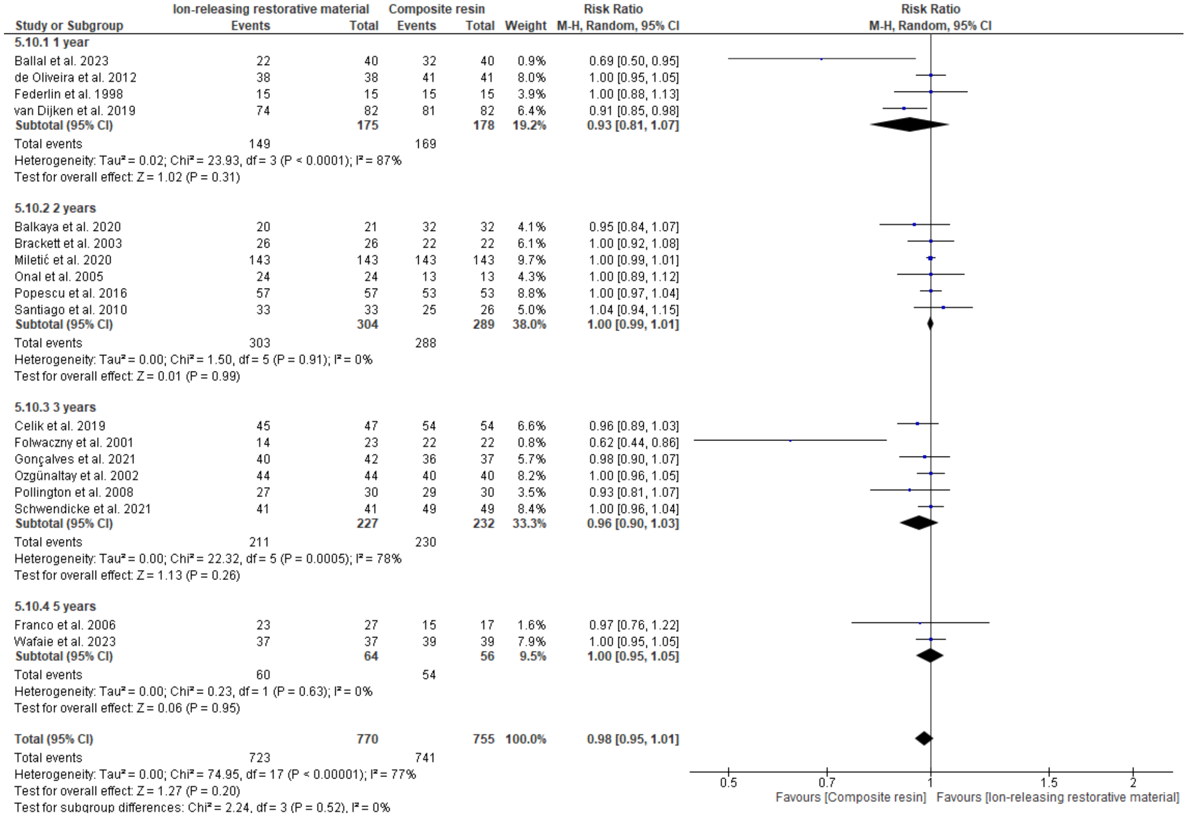

Figure S59. Absence of sensibility between IRR and CR in dental restorations by follow-up time

Study or Subgroup	Ion-releasing restorative material Events	Ion-releasing restorative material Total	Composite resin Events	Composite resin Total	Weight	Risk Ratio M-H, Random, 95% CI
**5.11.1 1 year**						
Adeleke et al. 2012	131	131	106	106	29.6%	1.00 [0.98, 1.02]
Hussainy et al. 2018	32	33	32	34	0.8%	1.03 [0.93, 1.14]
Perdigão et al. 2012	25	26	24	27	0.3%	1.08 [0.93, 1.26]
**Subtotal (95% CI)**		**190**		**167**	**30.7%**	**1.02 [0.95, 1.10]**
Total events	188		162			
Heterogeneity: Tau² = 0.00; Chi² = 4.45, df = 2 (P = 0.11); I² = 55%						
Test for overall effect: Z = 0.56 (P = 0.58)						
**5.11.2 2 years**						
Albelasy et al. 2024	27	27	27	27	1.7%	1.00 [0.93, 1.07]
Balkaya et al. 2020	21	21	32	32	1.4%	1.00 [0.93, 1.08]
Miletić et al. 2020	143	143	143	143	44.4%	1.00 [0.99, 1.01]
Toz-Akalin et al. 2023	28	28	29	29	1.8%	1.00 [0.94, 1.07]
Türkün et al. 2008	50	50	50	50	5.5%	1.00 [0.96, 1.04]
**Subtotal (95% CI)**		**269**		**281**	**54.9%**	**1.00 [0.99, 1.01]**
Total events	269		281			
Heterogeneity: Tau² = 0.00; Chi² = 0.00, df = 4 (P = 1.00); I² = 0%						
Test for overall effect: Z = 0.00 (P = 1.00)						
**5.11.3 3 years**						
Celik et al. 2019	47	47	54	54	5.6%	1.00 [0.96, 1.04]
Koc Vural et al. 2021	47	47	43	43	4.5%	1.00 [0.96, 1.04]
Schwendicke et al. 2021	41	41	49	49	4.4%	1.00 [0.96, 1.04]
**Subtotal (95% CI)**		**135**		**146**	**14.4%**	**1.00 [0.98, 1.02]**
Total events	135		146			
Heterogeneity: Tau² = 0.00; Chi² = 0.00, df = 2 (P = 1.00); I² = 0%						
Test for overall effect: Z = 0.00 (P = 1.00)						
**Total (95% CI)**		**594**		**594**	**100.0%**	**1.00 [0.99, 1.01]**
Total events	592		589			
Heterogeneity: Tau² = 0.00; Chi² = 2.19, df = 10 (P = 0.99); I² = 0%						
Test for overall effect: Z = 0.11 (P = 0.91)						
Test for subgroup differences: Chi² = 0.31, df = 2 (P = 0.86), I² = 0%						

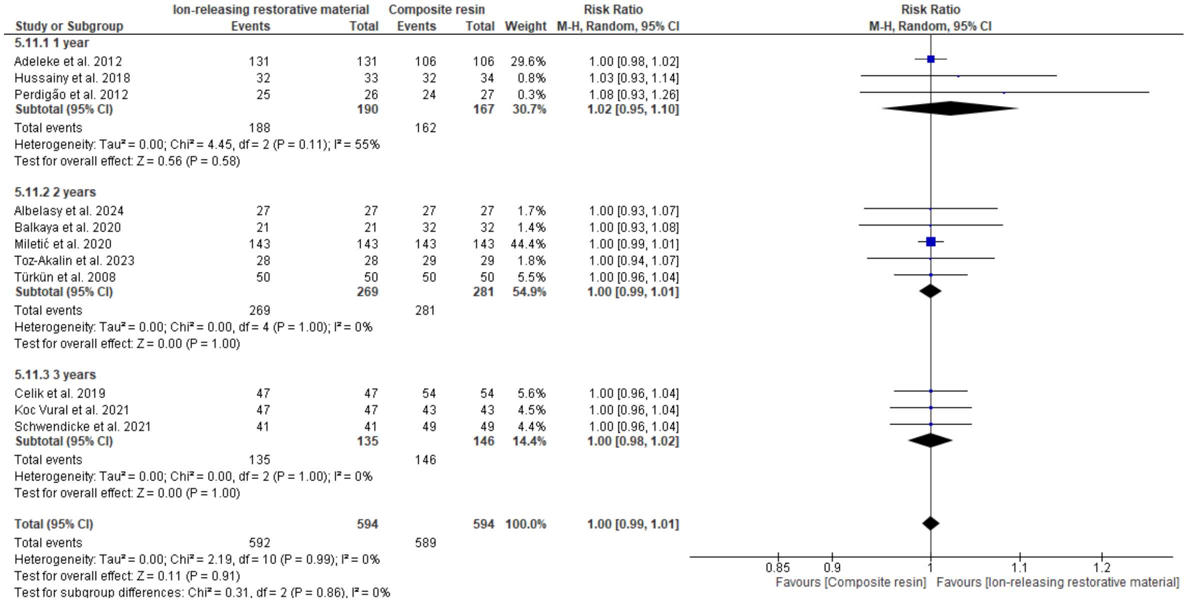

Figure S60. Adequate periodontal tissue between IRR and CR in dental restorations by follow-up time

Study or Subgroup	Ion-releasing restorative material Events	Ion-releasing restorative material Total	Composite resin Events	Composite resin Total	Weight	Risk Ratio M-H, Random, 95% CI
**5.12.1 2 years**						
Miletić et al. 2020	144	144	144	144	73.2%	1.00 [0.99, 1.01]
Türkün et al. 2008	50	50	50	50	15.8%	1.00 [0.96, 1.04]
**Subtotal (95% CI)**		**194**		**194**	**89.0%**	**1.00 [0.99, 1.01]**
Total events	194		194			
Heterogeneity: Tau² = 0.00; Chi² = 0.00, df = 1 (P = 1.00); I² = 0%						
Test for overall effect: Z = 0.00 (P = 1.00)						
**5.12.2 3 years**						
Celik et al. 2019	47	47	52	54	6.1%	1.04 [0.97, 1.11]
Gonçalves et al. 2021	42	42	36	37	4.9%	1.03 [0.96, 1.11]
**Subtotal (95% CI)**		**89**		**91**	**11.0%**	**1.03 [0.98, 1.08]**
Total events	89		88			
Heterogeneity: Tau² = 0.00; Chi² = 0.02, df = 1 (P = 0.88); I² = 0%						
Test for overall effect: Z = 1.33 (P = 0.18)						
**Total (95% CI)**		**283**		**285**	**100.0%**	**1.00 [0.99, 1.02]**
Total events	283		282			
Heterogeneity: Tau² = 0.00; Chi² = 3.34, df = 3 (P = 0.34); I² = 10%						
Test for overall effect: Z = 0.43 (P = 0.66)						
Test for subgroup differences: Chi² = 1.66, df = 1 (P = 0.20), I² = 39.8%						

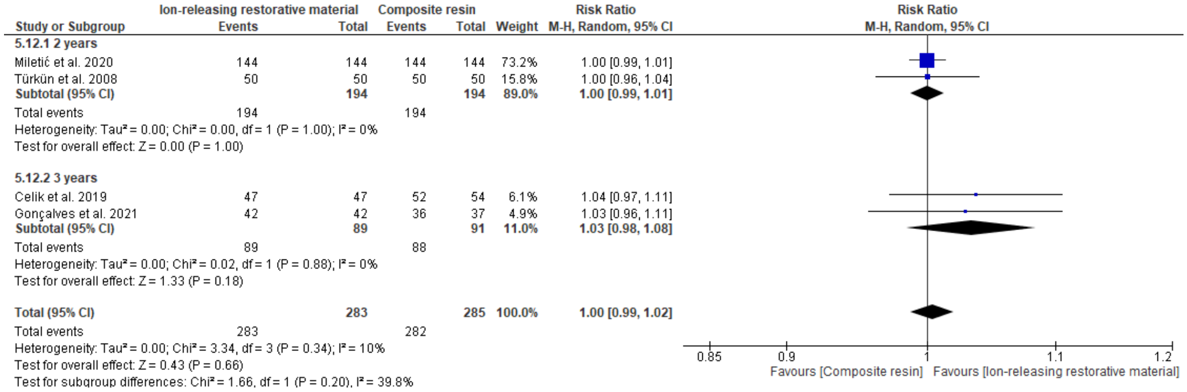

Supplement: Supplementary file 1 [file dentistry-12-00158-s001.zip › dentistry-2972548-supplementary.pdf]
